# DNA Methylation Signature of Childhood Chronic Physical Aggression in T Cells of Both Men and Women

**DOI:** 10.1371/journal.pone.0086822

**Published:** 2014-01-24

**Authors:** Claire Guillemin, Nadine Provençal, Matthew Suderman, Sylvana M. Côté, Frank Vitaro, Michael Hallett, Richard E. Tremblay, Moshe Szyf

**Affiliations:** 1 Department of Pharmacology & Therapeutics, McGill University, Montreal, Quebec, Canada; 2 Research Unit on Children’s Psycho-Social Maladjustment and Ste-Justine Hospital Research Center, University of Montreal, Montreal, Quebec, Canada; 3 Sackler Program for Epigenetics and Psychobiology, McGill University, Montreal, Quebec, Canada; 4 Department of Psychology and Pediatrics, University of Montreal, Montreal, Quebec, Canada; 5 School of Public Health and Population Sciences, University College Dublin, Dublin, Ireland; 6 INSERM U669, Paris, France; 7 McGill Centre for Bioinformatics, McGill University, Montreal, Quebec, Canada; 8 School of Psycho-Education, University of Montreal, Montreal, Quebec, Canada; 9 School of Social and Preventive Medicine, University of Montreal, Montreal, Quebec, Canada; Dartmouth Medical School, United States of America

## Abstract

**Background:**

High frequency of physical aggression is the central feature of severe conduct disorder and is associated with a wide range of social, mental and physical health problems. We have previously tested the hypothesis that differential DNA methylation signatures in peripheral T cells are associated with a chronic aggression trajectory in males. Despite the fact that sex differences appear to play a pivotal role in determining the development, magnitude and frequency of aggression, most of previous studies focused on males, so little is known about female chronic physical aggression. We therefore tested here whether or not there is a signature of physical aggression in female DNA methylation and, if there is, how it relates to the signature observed in males.

**Methodology/Principal Findings:**

Methylation profiles were created using the method of methylated DNA immunoprecipitation (MeDIP) followed by microarray hybridization and statistical and bioinformatic analyses on T cell DNA obtained from adult women who were found to be on a chronic physical aggression trajectory (CPA) between 6 and 12 years of age compared to women who followed a normal physical aggression trajectory. We confirmed the existence of a well-defined, genome-wide signature of DNA methylation associated with chronic physical aggression in the peripheral T cells of adult females that includes many of the genes similarly associated with physical aggression in the same cell types of adult males.

**Conclusions:**

This study in a small number of women presents preliminary evidence for a genome-wide variation in promoter DNA methylation that associates with CPA in women that warrant larger studies for further verification. A significant proportion of these associations were previously observed in men with CPA supporting the hypothesis that the epigenetic signature of early life aggression in females is composed of a component specific to females and another common to both males and females.

## Introduction

The development of physical aggression has been examined within large population-based longitudinal studies from birth to adulthood. Results show that acts of physical aggression begin by the end of the first year after birth for both boys and girls, increase in frequency from 2 to 4 years of age [1]–[4], and then decrease in frequency from school entry to adulthood [5]. However, a minority of children (3–7%) maintain a high frequency of physical aggression from childhood to adolescence [4]–[6]. Although both boys and girls use physical aggression from early childhood, fewer girls manifest physically aggressive behaviors on a frequent basis and girls also tend to reduce their use of physical aggression earlier in life than boys [3], [5], [7]–[9]. These sex differences tend to remain stable throughout childhood and adolescence [9]. Women with atypical high levels of childhood aggression (chronic physical aggression, CPA) tend to fail in school, suffer from depression, are likely to mate with men with similar behaviour problem, become pregnant during adolescence, smoke during pregnancy, and use coercive behavior towards their children [10], [11].

Genetic epidemiological studies suggest that the frequency of childhood physical aggression is in part inherited [12]–[16]. Genetic association studies have also found several polymorphisms in critical genes involved in neurotransmission and hormonal regulation to associate with aggression in humans and in animals [17]. Moreover, genetics and environmental factors have been shown to interact in the expression of impulsive aggression in monkeys [12], [18] and violence in humans [19].

Very little work has been done to identify the mechanisms that might be responsible for these gene-environment associations with physical aggression. We hypothesized that DNA methylation is one such mechanism [4], [20]–[22]. It is now well-established that DNA sequence is complemented by epigenetic information including DNA methylation and histone modifications to program gene expression [23]. Evidence is emerging that in addition to its role in regulating gene expression during differentiation, the DNA methylation pattern is responsive to external environmental exposures including the social environment [22] in animals [24]–[32] and in humans [33]–[37].

Importantly, DNA methylation alterations associated with social exposures are not restricted to the brain but can also be detected in white blood cells (WBC) DNA [32], [33], [35], [36], [38]–[46]. We have recently shown that differential DNA methylation of the serotonin transporter gene promoter (SLC6A4) in T cells and monocytes is associated with *in vivo* measures of human brain serotonin synthesis and childhood physical aggression in men [42]. Moreover, we have shown that young adult males on a chronic physical aggression trajectory between age 6 and 15 years had differential DNA methylated regions located in the genomic loci of cytokines and related transcription factors in T cells and monocytes, compared to males with the same background who did not follow such a high physical aggression trajectory (control group) [47]. In addition, using whole genome mapping method to analyse the DNA methylation profile of men on a CPA trajectory, we have recently found that men with CPA had a T cell DNA methylation signature distinct from men from the control group (Provençal et al., under review in PLoS One).

To test the hypothesis that T cell DNA methylation profiles are associated with childhood physical aggression in females and, if so, to compare them with the association found in male DNA, we analyzed genome-wide promoter methylation profiles in blood T cells of adult female participants. We recruited women who have been on a chronic physical aggression (CPA) trajectory between 6 and 12 years of age and compared them to women with the same background who followed normal physical aggression (NPA) trajectories [5]. Methylation profiles were created using the method of methylated DNA immunoprecipitation (MeDIP) followed by microarray hybridization, and analyses of these profiles revealed regions of significant DNA methylation patterns associated with CPA in women. We then compared these results with those previously observed in men from the same cohort (Provençal et al., under review in PLoS One).

## Materials and Methods

### Ethics Statement

After complete description of the study to the subject, all participants provided written informed consent. The study was carried out in accordance with the Declaration of Helsinki, and was approved by the research ethics committee of the University of Montreal pediatric hospital (St-Justine Hospital).

### Participants

The participants of this study are adult women of European descent, selected from a large sample of boys and girls who were first assessed when they were attending kindergarten in the province of Quebec’s French-speaking public schools in 1986–1987 [5], the Quebec Longitudinal Study of Kindergarten Children (QLSKC). The frequency of physical aggression was assessed for each participant by his school teachers between kindergarten (6 years old) and high school (12 years old). Developmental trajectories of physical aggression were previously identified for the entire sample of the longitudinal study [5]. Two groups of females were drawn from this cohort: 1) The Chronic Physical Aggression group is composed of women who were on a consistently high physical aggression trajectory from age 6 to 12 years (CPA); 2) The control group included women who did not have a history of chronic physical aggression from age 6 to 12 years (NPA). All participants were born in families with a low socioeconomic status and were living within 200 km from our laboratory at the time of recruitment in order to isolate blood cells and extract DNA from fresh blood draw. A total of 5 eligible women (out of 19) belonging to the CPA trajectory agreed to participate and were included in the study. To avoid having an imbalanced control group we included only 16 women in the control group. Due to technical reasons (insufficient DNA extracted from the sample) we had to drop two individuals from the control group. In addition to physical aggression, other behavioral problems, such as hyperactivity, were also rated from age 6 to 15 and violence at 21 years of age (See Methods S1 for details). Characteristics of the 2 groups are presented in Table 1.

**Table 1:** Characteristics of the chronic physical aggression (CPA) group and normal physical aggression (NPA) group.

	Mean ± SD or % (n)								
Variables	NPA				CPA				
Age at blood draw	24.19	±	0.54	(14)	25	±	1	(5)	t(4.76) = 1.738, P = 0.146
Familial adversity score&	0.192	±	0.197	(14)	0.414	±	0.387	(5)	t(19) = 1.738, P = 0.098
Psychiatric record (21 years old)	63%			(10/14)	80%			(4/5)	F exact, 2 tailled: 0.624
Criminal record (21 years old)	31%			(5/14)	20%			(1/5)	F exact, 2 tailled: 1.000
Hyperactivity (6 to 12 years)	0.585	±	0.647	(14)	1.748	±	0.937	(5)	t(19) = 3.161, P = 0.005
Oppositional disorder (6 to 12 years)	0.927	±	0.709	(14)	4.488	±	0.813	(5)	t(19) = 9.495, P = 0.000
Anxiety (6 to 12 years)	2.667	±	1.393	(14)	2.652	±	1.613	(5)	t(19) = −0.19, P = 0.985
Attention deficit (6 to 12 years)	2.196	±	1.567	(14)	2.528	±	1.114	(5)	t(19) = 0.436, P = 0.667

& include mother and father occupational score, familial status (monoparental vs biparental), mother and father at birth of first child and the years of schooling of the mother and father.

(Additional information on the characteristics of the groups can be found in Methods S1). For further validation by Illumina 450K we also included the adult males from the same longitudinal studies (8 CPA and 12 controls) that were previously used in the MeDIP-microarrays analysis (Provençal et al., under review in PLoS One).

### DNA Methylation Analysis by MeDIP and Micoarrays Hybridization

Genomic DNA was extracted from T cells purified from blood draw by immunomagnetic positive selection using CD3 antibody as previously described [32], [42], [47]. Methylated DNA immunoprecipitation was performed as previously described [31], [32], [38] using anti-5methylCytosine antibody from Calbiochem. The specificity of the immunoprecipitation was assessed by regular PCR on two endogenous control genes, H19 (methylated control) and β-actin (unmethylated control) and two plasmids that were added to the DNA prior to sonication, GFP (unmethylated control) and Luciferase (*in vitro* methylated control) using the following primers: H19: forward (5′-GAGCCGCACCAGATCTTCAG -3′), reverse (5′-TTGGTGGAACACACTGTGATCA-3′); β-actin: forward (5′-CCAACGCCAAAACTCTCCC-3′), reverse (5′-AGCCATAAAAGGCAACTTTCG-3′); GFP: forward (CAAGGGCGAGGAGCTGTT), reverse (CGGCCATGATATAGACGTTG); Luciferase: forward (5′-AGAGATACGCCCTGGTTCC-3′), reverse (5′-CCAACACCGGCATAAAGAA-3′). The input and bound fractions were then amplified using Whole Genome Amplification kit (Sigma) and labeled for microarray hybridization with either Cy3-dUTP or Cy5-dUTP (Perkin Elmer) respectively using the CGH labeling kit (Invitrogen) following the manufacturer’s instructions. The labeled input and bound DNA samples were hybridized to a custom designed 180K promoter tiling array (Agilent technologies) that contained probes covering all transcription start sites at intervals from 1000 bp upstream to 200 bp downstream of all genes described in Ensembl (version 54) and within 250 bp of the ∼400 microRNAs from miRBase, all at 100 bp-spacing. The array covered 69,818 transcription start sites corresponding to 20,318 genes. All the steps of hybridization, washing, and scanning were performed following the Agilent protocol for ChIP-on-chip analysis. After microarray scanning, probe intensities were extracted from scan images using Agilent’s Feature Extraction 9.5.3 Image Analysis Software.

### MeDIP Microarray Analysis

Two replicate microarrays were hybridized and analysed for each of the 19 samples. Quality control involved visual inspection of microarray scan images, scanning software reports and generation of MvA plots (i.e. plots of log(Cy5/Cy3) vs log(Cy5 x Cy3)) to identify microarrays with dye biases or low signal. Microarrays were normalized using background subtraction followed by quantile normalization of log2-ratios of bound (Cy5) and input (Cy3) channel intensities. Differential methylation between groups of samples was determined at the probe and promoter levels to ensure both statistical significance and biological relevance as previously described [38]. At the probe level, a modified t-statistic was computed for each probe to identify probe intensity differences between CPA and NPA groups using the ‘limma’ package [48] of Bioconductor [49]. Then, promoter-level methylation differences were identified as those promoters significantly enriched with probes having positive or negative t-statistics using the Wilcoxon rank-sum test. A probe and the containing promoter were called *differentially methylated* if the p-value of the probe t-statistic was at most 0.05 (uncorrected for multiple testing), log2-fold difference between the groups was at least 0.25, and the false discovery rates (FDR) of the promoter-level statistic was at most 0.05. The microarray data have been deposited in Gene Expression Omnibus (GEO) at NCBI (www.ncbi.nlm.nih.gov/geo/) under the accession number GSE53193.

A Bayesian deconvolution method was used to estimate methylation levels from the microarray data [51] and, as expected, estimated promoter methylation levels were found to be significantly anti-correlated (p = 2.3e-25) with previously published gene expression levels of the same human cell type (http://biogps.org/) (Figure S1).

All functional analysis was done using Ingenuity Pathway Analysis [50] with the default parameters.

### MeDIP Microarray Validation

Two approaches were used to validate the regions called differentially methylated between CPA and NPA subjects from the MeDIP-microarrays analysis; a gene-specific approach using quantitative real-time PCR on the MeDIP fractions (Q-MeDIP) and a genome-wide approach using Illumina Infinium HumanMethylation450 BeadChip® (Illumina).

Gene-specific real-time PCR validation of microarray was performed on the amplified bound fraction for the same subjects used for microarray experiments (n = 5 CPA and n = 14 NPA). Real-time PCR experiments were performed using a LightCycler and SYBR green reagent (Roche Diagnostics). Primers and detailed conditions are available upon request. The Ct (cycle thresholds) for all bound samples was converted to a relative quantity scale using a standard curve of diluted fragmented human DNA. We normalized the results by calculating the ratio of the amplified specific gene over the amplified methylated control. The methylated control used for normalization was the *in vitro* methylated luciferase plasmid that was added in equal amount to each sample prior to the MeDIP. The Q-MeDIP analyses of the methylated control show no difference between the groups.

Whole-genomic validation of MeDIP microarray data was performed using Illumina 450K arrays. This technique was applied to validate both the male (Provençal et al. under review in PLoS One) and female samples. Pooled samples were used since only limited amount of DNA was available for many of the subjects. Three different pools of genomic DNA were made per group: men CPA (2 or 3 different subjects per pool), men NPA (4 different subjects per pool), women CPA (2 different subjects per pool) and women NPA (4 or 5 different subjects per pool). Genomic DNA was quantified using Picogreen protocol (Quant-iT™ PicoGreen® dsDNA Products, Invitrogen) and read on SpectraMAX GeminiXS Spectrophotometer. Bisulfite conversion was performed with 500 ng of genomic DNA using the EZ-96 DNA Methylation-Gold Kit (Zymo Research) according to the manufacturer’s instructions. The Illumina Methylation 450K kit was used for the microarray experiment, as described by the manufacturer’s protocol, except that 8 µl of bisulfite converted material was used to initiate the amplification step. Arrays were scanned using the Illumina iScan Reader. Data analysis was performed using the Methylation module (version 1.9.0) of the GenomeStudio software (Illumina; version 2011.1). Significant differences in methylation levels between CPA and control groups were obtained using the Illumina custom method with a False Discovery Rate (FDR) <0.05. Beta-values with detection P-value >0.001 were removed from the analysis since they are considered to fall below the minimum intensity and threshold. All the probes that contained a SNP within 10 bp of the CpG site were excluded from the analysis to ensure equal annealing efficiency between the samples. The raw data have been deposited in Gene Expression Omnibus (GEO) at NCBI (www.ncbi.nlm.nih.gov/geo/) under the accession number GSE53193.

### Validation of Illumina 450K Microarrays

Primers were designed to generate amplicons <350 bp long corresponding to the sequence containing differentially methylated CGs identified by the Illumina 450K analysis. Bisulfite conversion of individual male and female samples was performed using 500 ng of genomic DNA using the EZ-96 DNA Methylation-Gold Kit (Zymo Research) according to the manufacturer’s instructions. PCR amplifications were performed with 15 ng of bisulfite DNA using TaKaRa EpiTaq HS polymerase (Cedarlane) on individual samples using the following primers: *ZNF366*: forward (5′-GAAGGTATTTATTTGAGAAAAAGAG-3′), reverse (5′-CCAACTCCTAAAATCTAAATAACTACAAC-3′). To calibrate the assay and control for amplification bias, we amplified the same regions from completely unmethylated (0%) or fully methylated (100%) or mixtures of bisulfite-converted EpiTect Control DNA (Qiagen). For pyrosequencing analysis, 20 µl of the bisulfite-PCR products were processed according to the manufacturer’s standard protocol (Qiagen). Sequencing reactions were performed on a PyroMark Q96 system using the PyroMark Gold Q96 Reagent Kit (Qiagen) according to the manufacturer’s instructions. The percentage methylation at each CpG site was calculated from the raw data using PyroMark Q96-CpG Software (Qiagen).

## Results

### T Cell Promoter Methylation Associated with CPA in Women

**Figure 1:**
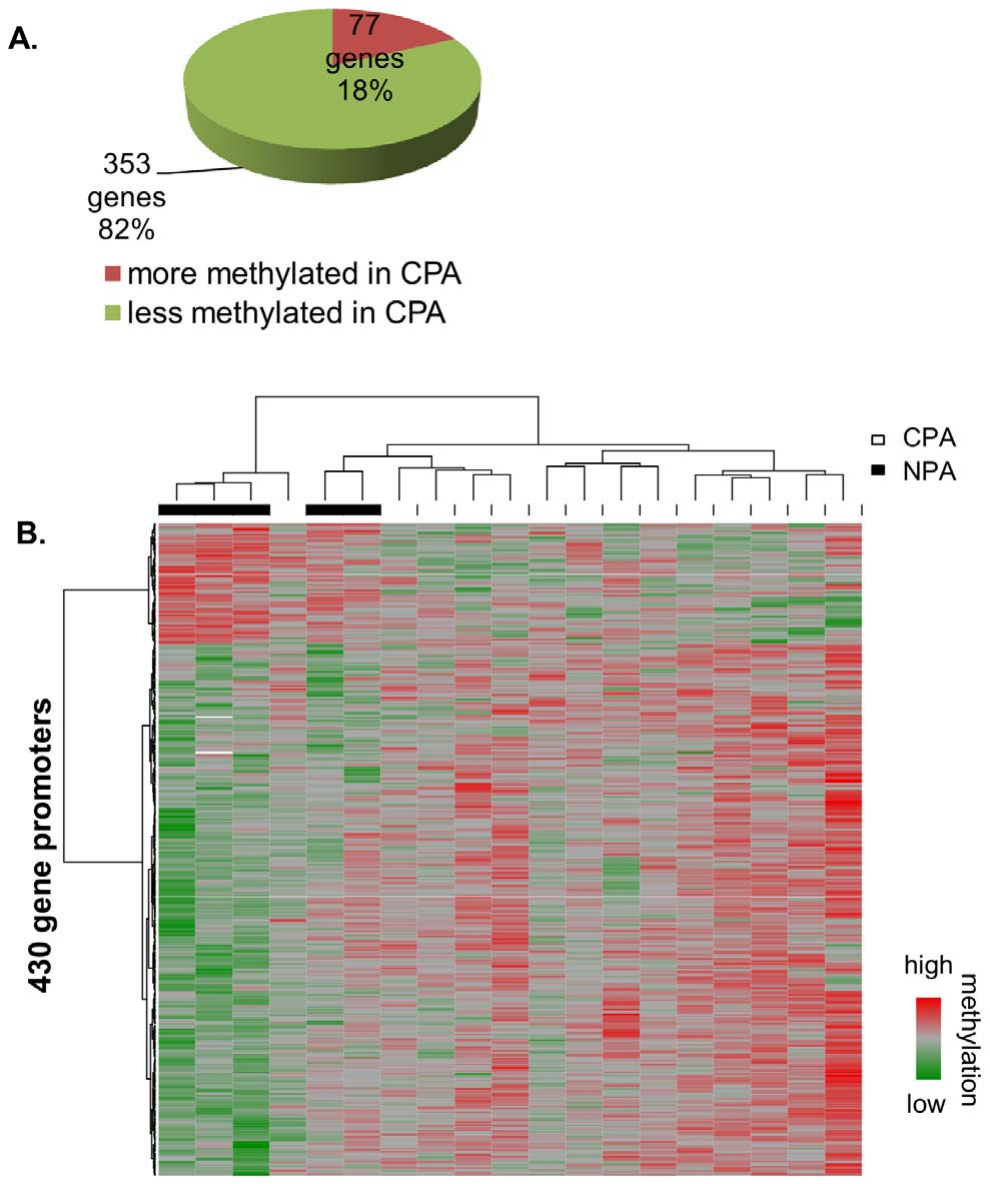
Gene promoters differentially methylated between women CPA (n = 5) and NPA (n = 14) in T cells. **A**. Numbers of promoters with significant methylation increases and decreases in CPA versus NPA (P<0.05; FDR<0.05). **B.** Heatmap depicts normalized intensities of microarray probes contained in promoters that best differentiate between CPA and NPA groups. Probes were selected from gene promoters called differentially methylated with respect to aggression groups (P≤0.05; FDR ≤0.05). One probe was selected for each gene, always the probe with the most extreme t-statistic. Heatmaps are colored so the median values on each row are gray, high values are red and low values are green. Red indicates higher methylation in a row and green indicates lower methylation. Clustering was performed using Ward’s hierarchical clustering algorithm with Pearson correlation distance as the distance metric. Rows correspond to promoters and columns to subjects.

Genome-wide promoter methylation profiles of T cell DNA from women on a CPA trajectory during childhood and adolescence were compared to women on normative physical aggression trajectories. We found 917 probes corresponding to 430 distinct gene promoters differentially methylated between aggression groups (P<0.05 and FDR <0.05, Spreadsheet S1). Of these promoters, 353 were less methylated and 77 were more methylated in the CPA group (Figure 1A). A heatmap of the probes in each of these gene promoters that best differentiate between the groups is shown in Figure 1B. The 25 gene promoters most significantly different between the two groups are listed in Table S1 (P<0.005 and FDR <0.01). Interestingly, the list of differentially methylated genes includes a few that have been previously associated with aggressive behavior in human and animals [17] (Table 2). For example, *Tryptophan Hydroxylase 2* (*TPH2*), *Corticotrophin Releasing Hormone binding protein* (*CRHBP*) and the glucocorticoid receptor (*NR3C1*) were all found to be less methylated in the CPA group.

**Table 2:** Differentially methylated gene promoters between women chronic physical aggression (CPA) and non-aggressive (NPA) groups previously shown to be associated with aggressive behavior.

Gene ID	Gene description	P-value	FDR	Distance from TSS	More methylated in	Association with aggressive behavior
TPH2	Tryptophan hydroxylase 2	0.03	0.02	-117	NPA	Genetic association (SNP) in human [55]
NR3C1	Glucocorticoid receptor	0.05	0.01	84	NPA	Genetic (SNP) association in pig [57]
CRHBP	Corticotropin-releasing factor-binding protein	0.04	0.01	-82	NPA	KO mice show less maternal aggression [83]

We selected 19 of these regions called differentially methylated for validation by Q-MeDIP (Figure S2A) as described in the Methods section. Of these, 10 were found to be differentially methylated by Q-MeDIP and, overall, the fold-change differences identified by Q-MeDIP were highly correlated with fold-change differences identified by microarray (R = 0.849, P<0.001 by Pearson’s correlation, Figure S2B).

### Comparison between Differentially Methylated Promoters Associated with Physical Aggression in Men and Women

**Figure 2:**
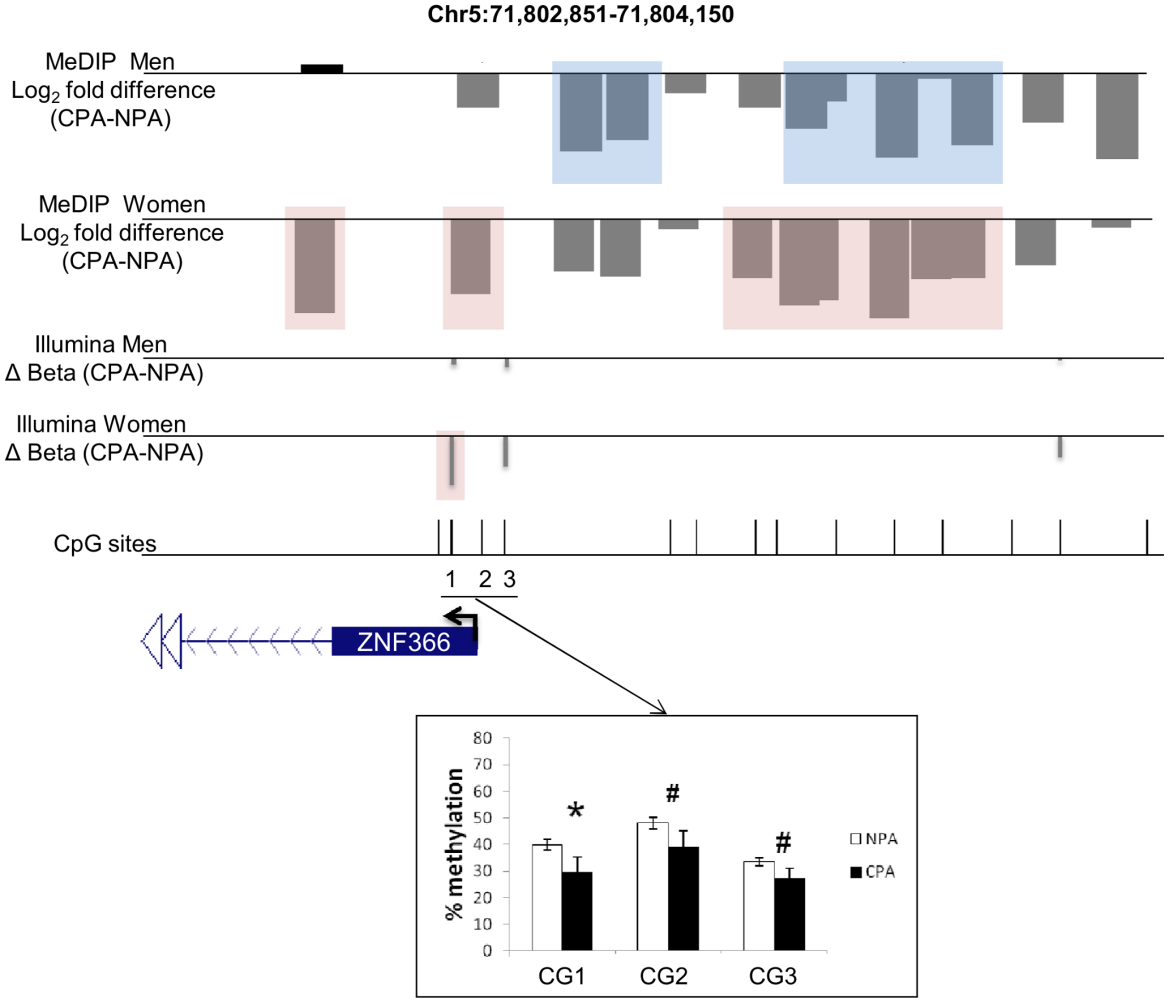
Methylation pattern associated with aggression in women and in men of the *ZNF366* gene promoter region analyzed by MeDIP microarrays and validated using Illumina 450K arrays and pyrosequencing. Expanded views from the UCSC genome browser of *ZNF366* gene promoter region located on chromosomes 5 are depicted. The first and second tracks show the average MeDIP microarray probe log2-fold differences (scale: −0.6 to 0.6 for both tracks) between chronic physical aggressive (CPA) and non-aggressive (NPA) groups for men and women T cells. In black are probes that are more methylated and in gray are those that are less methylated in the CPA group. Highlighted in blue and in pink are regions significantly differentially methylated between the groups in men and in women, respectively. The third and fourth track shows delta Beta values (average Beta CPA- average Beta NPA) obtained for each CpG site analyzed by Illumina 450K arrays in men and in women (scale: −0.2 to 0.2 for both tracks). The last track indicates the location of individual CpG sites using black lines. In this region, three CpG sites were analyzed by pyrosequencing (underlined). The bar graph shows the average methylation levels for each CpG sites in the women CPA and NPA groups as determined by pyrosequencing. Error bars show the SEM and * indicate P<0.05 and # P<0.1.

A previous study, conducted in our laboratory, analyzing genome-wide promoter methylation profiles in the T cells of adult males from the same longitudinal cohort as the one studied here (Provençal et al., under review in PLoS One), found 448 gene promoters to be differentially methylated in men with CPA. Of these, 31 gene promoters belong to the list of gene promoters found differentially methylated here in females (Table 3), a significant overlap (P<0.0001, Fisher’s exact test). Within the promoters of these genes, there are 32 common differentially methylated probes, indicating that a significant portion of the overlap is actually due to identical genomic sites being differentially methylated. One example is the *ZNF366* gene promoter region where the MeDIP microarray analysis identified a region which methylation pattern is associated with aggression in both women and men (Figure 2).

**Table 3:** Differentially methylated gene promoters associated with childhood physical aggression in common between men and women using MeDIP-microarrays.

		Probe coordinates (hg19)		Women			Men		
Gene ID	Gene Name	Women	Men	P-value	FDR	More methylated in	P-value	FDR	More methylated in
**AGTR1**	Type-1 angiotensin II receptor	chr3∶148447513–148447571		0.013	0.037	NPA	0.0004	0.122	NPA
**AMICA1**	Adhesion molecule interacting with CXADR antigen 1	chr11∶118084814–118084872		0.036	0.003	NPA	0.006	0.092	NPA
		chr11∶118084747–118084805		0.045	0.003	NPA	0.0002	0.092	NPA
**ANKRD50**	Ankyrin repeat domain-containing protein 50	chr4∶125632637–125632695		0.024	0.0004	NPA	0.046	0.018	NPA
**ARL14EP**	ADP-ribosylation factor-like 14 effector protein	chr11∶30343875–30343929		0.040	0.005	NPA	0.006	0.184	NPA
**ASPH**	Aspartate beta-hydroxylase	chr8∶62602098–62602156		0.016	0.005	NPA	0.037	0.177	CPA
		chr8∶62602789–62602847		0.040	0.005	NPA	0.042	0.177	CPA
**ATP2B2**	Plasma membrane calcium ATPase isoform 2	chr3∶10491749–10491797		0.037	0.002	CPA	0.045	0.015	CPA
CSHL1	Chorionic somatomammotropin hormone-like 1	chr17∶61988733–61988777	chr17∶61988904–61988948	0.015	0.043	NPA	0.004	0.124	NPA
**DSE**	Dermatan sulfate epimerase	chr6∶116691485–116691543		0.045	0.019	NPA	0.009	0.153	NPA
DTNA	Dystrobrevin alpha	chr18∶32397825–32397884	chr18∶32398251–32398300	0.011	0.001	NPA	0.049	0.159	NPA
ERMN	Ermin	chr2∶158182438-158182497	chr2∶158182195–158182253	0.034	0.004	NPA	0.026	0.018	NPA
**GPR84**	Probable G-protein coupled receptor 84	chr12∶54758367–54758418		0.006	0.002	NPA	0.029	0.190	CPA
		chr12∶54758643–54758690		0.047	0.002	NPA	0.012	0.190	CPA
IL1RN	Interleukin 1 receptor antagonist	chr2∶113885113–113885163	chr2∶113884993–113885041	0.006	0.009	NPA	0.015	0.080	NPA
**LTBP1**	Latent transforming growth factor beta binding protein 1	chr2∶33359024–33359077		0.011	0.007	NPA	0.001	0.061	NPA
**METTL17**	Methyltransferase like 17	chr14∶21457348–21457398		0.026	0.002	NPA	0.001	0.116	NPA
**MGP**	Matrix Gla protein	chr12∶15039226–15039284		0.026	0.0004	NPA	0.044	0.018	NPA
		chr12∶15039414–15039472		0.045	0.0004	NPA	0.044	0.018	NPA
NDUFS2	NADH dehydrogenase [ubiquinone] iron-sulfur protein 2, mitochondrial Precursor	chr1∶161169390–161169435	chr1∶161169441–161169489	0.034	0.039	NPA	0.045	0.149	NPA
NXF5	Nuclear RNA export factor 5	chrX:101145428–101145487	chrX:101113150–101113203	0.004	0.026	NPA	0.013	0.062	NPA
**PCDH8**	Protocadherin 8	chr13∶53423013–53423056		0.008	0.013	NPA	0.036	0.167	CPA
**PCDHB2**	Protocadherin beta 2	chr5∶140474015–140474073		0.007	0.026	NPA	0.006	0.092	NPA
**PCDHB8**	Protocadherin beta 8	chr5∶140557507–140557559		0.028	0.028	NPA	0.047	0.092	NPA
		chr5∶140557504–140557558		0.043	0.028	NPA	0.005	0.092	NPA
PITPNM2	Membrane-associated phosphatidylinositol transfer protein 2	chr12∶123595427–123595472	chr12∶123595504–123595548	0.033	0.002	NPA	0.027	0.076	NPA
PLAC1L	Placenta-specific 1-like	chr11∶59807048–59807107	chr11∶59807734–59807790	0.008	0.035	NPA	0.019	0.186	NPA
PPARG	Peroxisome proliferator-activated receptor gamma	chr3∶12392616–12392675	chr3∶12393071–12393121	0.003	0.00003	NPA	0.012	0.012	NPA
**SCN11A**	Sodium channel protein type 11 subunit alpha	chr3∶38992140–38992192		0.017	0.019	NPA	0.015	0.112	NPA
		chr3∶38992084–38992127		0.009	0.019	NPA	0.037	0.112	NPA
TAS2R42	Taste receptor type 2 member 42	chr12∶11339249–11339308	chr12∶11340282–11340341	0.030	0.019	NPA	0.002	0.116	NPA
**TGIF1**	TGFB-induced factor homeobox 1	chr18∶3447166–3447224		0.021	0.002	NPA	0.001	0.018	NPA
**TMEM225**	Transmembrane protein 225	chr11∶123756482–123756538		0.019	0.012	NPA	0.010	0.159	NPA
		chr11∶123756404–123756452		0.029	0.012	NPA	0.013	0.159	NPA
**UBN1**	Ubinuclein 1	chr16∶4897859–4897902		0.004	0.016	CPA	0.018	0.173	CPA
**UCK2**	Uridine-cytidine kinase 2	chr1∶165796344–165796396		0.0004	0.014	NPA	0.0003	0.149	NPA
		chr1∶165796466–165796524		0.001	0.014	NPA	0.009	0.149	NPA
		chr1∶165796265–165796308		0.004	0.014	NPA	0.007	0.149	NPA
		chr1∶165796456–165796514		0.002	0.014	NPA	0.001	0.149	NPA
USP44	Ubiquitin carboxyl-terminal hydrolase 44	chr12∶95945675–95945734	chr12∶95945124–95945170	0.049	0.014	CPA	0.013	0.062	NPA
**ZNF366**	Zinc finger protein 366	chr5∶71803803–71803855		0.046	0.0004	NPA	0.003	0.018	NPA
		chr5∶71803606–71803650		0.006	0.0004	NPA	0.013	0.018	NPA
		chr5∶71803713–71803760		0.005	0.0004	NPA	0.001	0.018	NPA

### Functional Relevance of the Differentially Methylated Promoters Associated with CPA

To delineate if the list of genes differentially methylated between CPA and NPA are grouped into biological functions and pathways we used Ingenuity Pathway Analysis (IPA) software. In women, IPA identified 74 functional categories significantly enriched with the affected genes including “Cell signaling and interaction”, “Inflammatory response” and “Behavior” (Table S2) as well as specific canonical signalling pathways including CCR5, TEC kinases, G-protein Gαi and IL-10 (Table S3).

Aggression might also affect the methylation status of a specific group of genes by targeting their upstream regulators. Using IPA we were able to identify a set of potential upstream regulators including transcription factors, growth factors and transmembrane receptors. These potential upstream regulators were identified to target a significant number of genes associated with aggression in women (Table S4). The list includes many molecules that play an important role in the regulation of genes involved in immune and inflammatory responses, such as transcription factors FOS, GATA1 and GATA3, hepatocyte growth factor (HGF) and many cytokines (TNF, IFNG, IL1B, IL1A, IL17R, IL10, IL13, IL18).

Almost all of the functional categories significantly enriched with genes associated with physical aggression in women were previously observed in men (Provençal et al., under review in PLoS One) (70 out of 74) with “Behavior” being one of the top 5 categories (Table 4 and Table S5; P<0.0001 Fisher’s exact test), as well as twelve out of the thirty enriched canonical pathways (Table 4 and Table S6; P<0.0001 Fisher’s exact test) and common potential upstream regulators such as TNF and IL-1β involved in inflammatory response (Table 4 and Table S7).

**Table 4:** Functions and pathways enriched with genes whose methylation is associated with aggression in both sexes from IPA analysis (women n = 430 genes and men n = 448 genes).

Functional Categories	Group	P-value	# Genes
Cancer	women	2.47E-09;3.72E-03	221
	men	5.09E-08;2.74E-02	205
Respiratory Disease	women	5.27E-06;3.55E-03	126
	men	5.09E-08;2.04E-02	130
Cellular Growth and Proliferation	women	1.11E-07;3.97E-03	124
	men	3.15E-05;2.08E-02	7
Cellular Development	women	1.11E-07;4.29E-03	79
	men	3.15E-05;2.6E-02	64
Behavior	women	8.62E-04;2.16E-03	13
	men	2.75E-04;2.54E-02	10
**Canonical Pathways**	**Group**	**P-value**	**# Genes**
Granulocyte Adhesion and Diapedesis	women	1. 51E-04	12
	men	5.13E-04	11
Agranulocyte Adhesion and Diapedesis	women	2.51E-04	12
	men	8.71E-02	9
FXR/RXR Activation	women	5.25E-04	8
	men	9.77E-03	6
IL-10 Signaling	women	2.63E-03	6
	men	1.17E-02	5
Role of Cytokines in Mediating Communication between Immune Cells	women	4.07E-03	5
	men	3.80E-03	5
**Upstream Regulators**	**Group**	**P-value of overlap**	**# Genes**
TNF	women	5.49E-06	55
	men	6.61E-03	44
IL1B	women	1.37E-05	33
	men	3.16E-03	27
FOS	women	6.46E-05	23
	men	8.55E-03	18
Prostaglandin E2	women	3.34E-04	15
	men	1.03E-02	12
ADAMTS12	women	1.30E-02	3
	men	1.44E-02	3

### Genomic Organization of Differentially Methylated Promoters Associated with CPA

**Figure 3:**
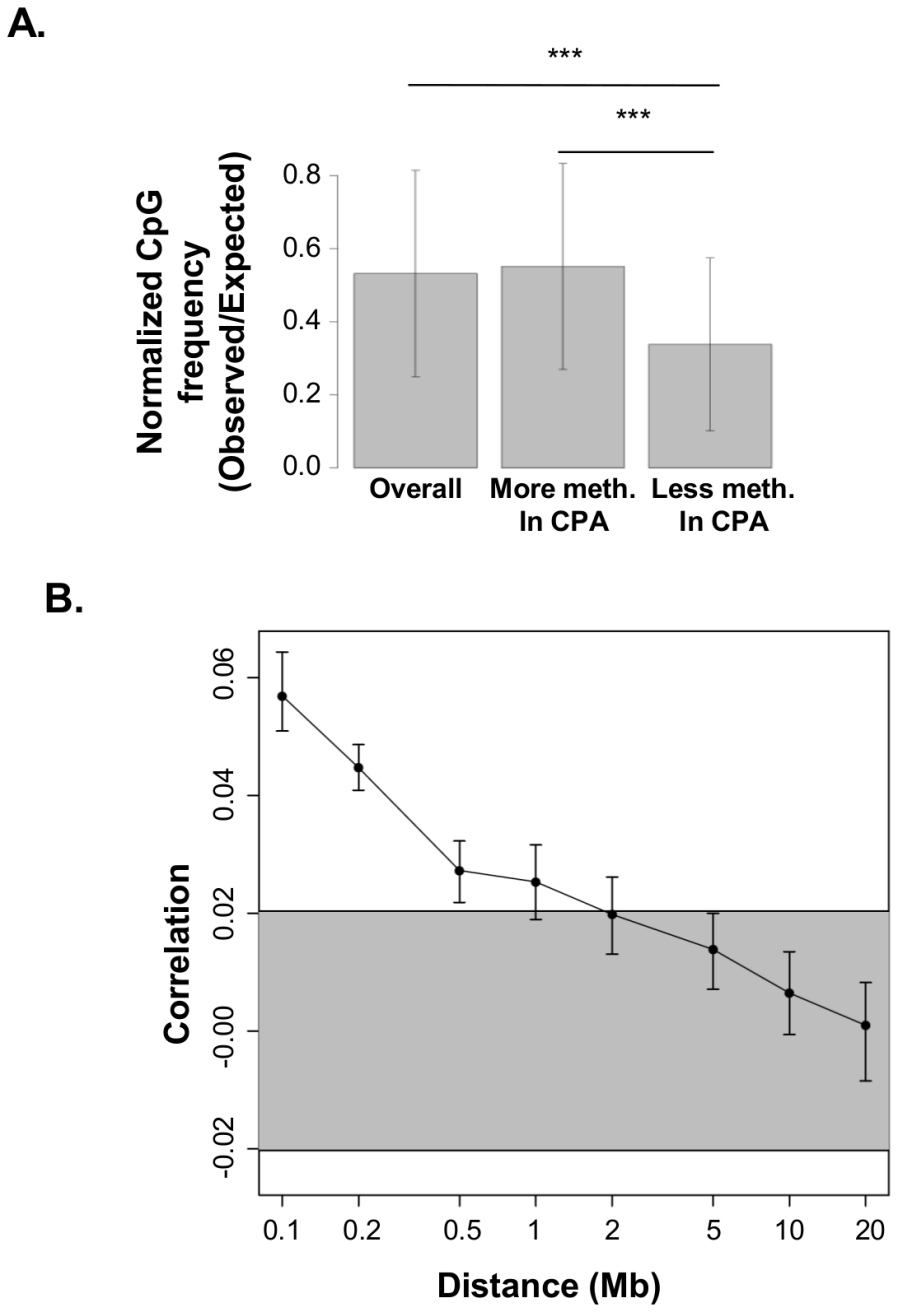
Genomic organization of differentially methylated promoters associated with CPA. A. CpG density of the differentially methylated promoters. Normalized CpG density is the density of CpG sites divided by the expected density calculated by multiplying the density of C sites by the density of G sites. *** indicate P<0.0001. B. The Pearson correlations of DNA methylation differences between CPA and NPA groups at various genomic distances are shown. Error bars denote 95% confidence intervals obtained from 1000 bootstraps composed of randomly selected probe pairs with replacement. The grey highlight shows the expected 95% confidence interval for correlations of probe pairs independent of their distance. Independence was simulated by with 500 random permutations of the probe coordinates. This confidence interval does not overlap with the error bars associated with distances less than 1.5Mb suggesting the existence of systematic dependencies between methylation differences at distances up to 1.5Mb.

In addition to functional organization, the differentially methylated promoters exhibit genomic organization. As previously observed in men (Provençal et al., under review in PLoS One), regions with higher methylation in CPA women contained higher than expected CpG densities and regions with lower methylation in CPA women contained lower than expected CpG densities (Figure 3A). Although CPA associated methylation differences are distributed throughout the genome in both men and women, the differences typically appeared in clusters such that methylation differences at any given genomic location were weakly but significantly predictive of methylation differences up to 1.5Mb away in women (Figure 3B) and 2Mb away in men (Provençal et al., under review in PLoS One).

### Validation of the MeDIP-microarray Data using Illumina Human Methylation 450K Arrays

To further confirm the results obtained using MeDIP-microarrays, we generated and analyzed new methylation profiles using the Infinium Human Methylation 450 BeadChip®. The Illumina 450K approach is an alternative to the MeDIP approach appropriate for validation because it is based on bisulfite conversion rather than antibody affinity. Although it has lower coverage than the MeDIP microarray, it has higher resolution (single-CpG resolution) and reasonable coverage with at least one CpG site in 96% of known CpG islands and 99% of RefSeq gene promoters. Due to small amounts of remaining DNA, samples were pooled to produce three sample pools per aggression group (both male and female), each pool hybridized to an Illumina 450K array and then the arrays analyzed to identify methylation differences between CPA and NPA sample pools. A total of 716 gene promoters were found to contain CpG sites differentially methylated between female CPA and NPA pools (P≤0.05 and FDR <0.05, Spreadsheet S2). Of these, 70 gene promoters were also found differentially methylated using the MeDIP-microarrays (Table S8; P<0.001, Fisher’s exact test), and 79 contain a differentially methylated CpG site in the male Illumina 450K microarrays (P<0.0001, Fisher’s exact test; Table 5). These 79 gene promoters differentially methylated in both men and women according to the Illumina platform include 7 gene promoters differentially methylated in women and 3 gene promoters differentially methylated in men according to our MeDIP-microarray data. One example is the *ZNF366* gene promoter region where both the MeDIP microarray and Illumina 450K analysis identified a region which methylation pattern is associated with aggression in women (Figure 2).

**Table 5:** Differentially methylated gene promoters associated with childhood physical aggression in common between men and women using Illumina 450K arrays.

		Illumina CpG ID		Women		Men	
Closest Gene ID	Gene name	Women	Men	P-value	Δ Beta value (CPA-NPA)	P-value	Δ Beta value (CPA-NPA)
SEPT9	septin 9	cg17922695	cg15044248	P<0.01	0.18	P<0.001	-0.12
ACPT	acid phosphatase	cg06590444		P<0.001	0.15	P<0.001	0.16
ALG10	alpha-1,2-glucosyltransferase	cg23762105		P<0.001	0.16	P<0.001	0.15
AP3M2	adaptor-related protein complex 3, mu 2 subunit	cg18404652	cg20603637	P<0.05	0.15	P<0.001	-0.09
APMAP	adipocyte plasma membrane associated protein	cg20661985	cg06937882	P<0.001	-0.16	P<0.05	0.11
ARPP21	cAMP-regulated phosphoprotein, 21kDa	cg01307174	cg24632247	P<0.05	-0.13	P<0.05	-0.09
ARRDC2	arrestin domain containing 2	cg10614223	cg08021780	P<0.05	0.15	P<0.05	-0.09
ATHL1	ATH1, acid trehalase-like 1	cg08829299		P<0.01	-0.12	P<0.05	-0.11
		cg22280068		P<0.001	0.20	P<0.001	0.27
B3GNT7	UDP-GlcNAc:betaGal beta-1,3-N-acetylglucosaminyltransferase 7	cg06745030	cg26146617	P<0.05	-0.13	P<0.05	-0.11
BCAT1	branched chain amino-acid transaminase 1, cytosolic	cg21500300	cg20399616	P<0.05	-0.12	P<0.001	-0.16
BCL2L14	BCL2-like 14 (apoptosis facilitator)	cg27366072	cg18223430	P<0.05	-0.11	P<0.05	0.13
BGLAP	bone gamma-carboxyglutamate (gla) protein	cg24670453		P<0.001	-0.21	P<0.05	-0.15
BRD2	bromodomain containing 2	cg00326788	cg09547081	P<0.001	-0.23	P<0.05	-0.07
C17orf64	chromosome 17 open reading frame 64	cg12131208	cg09695735	P<0.05	-0.09	P<0.001	-0.12
CAT	catalase	cg01847719		P<0.01	0.11	P<0.001	-0.13
CCR3	chemokine (C-C motif) receptor 3	cg14312439		P<0.05	-0.13	P<0.001	0.11
CDX1	caudal type homeobox 1	cg11117637		P<0.05	-0.08	P<0.05	-0.11
CHN2	chimerin 2	cg12469381		P<0.001	-0.20	P<0.01	-0.17
CMTM1	CKLF-like MARVEL transmembrane domain containing 1	cg05477582		P<0.001	-0.17	P<0.01	-0.10
CPM	carboxypeptidase M	cg02266731	cg21548096	P<0.05	-0.14	P<0.01	-0.07
CRISP2	cysteine-rich secretory protein 2	cg26715042	cg13094036	P<0.05	0.13	P<0.001	-0.10
CYGB	cytoglobin	cg24879415	cg02396496	P<0.05	-0.12	P<0.01	-0.09
EPB49	erythrocyte membrane protein band 4.9 (dematin)	cg09316140		P<0.01	0.12	P<0.05	-0.09
FAM134B	family with sequence similarity 134, member B	cg00401101		P<0.01	-0.17	P<0.01	-0.16
FBXO27	F-box protein 27	cg18624102		P<0.001	-0.21	P<0.01	0.18
FGR	Gardner-Rasheed feline sarcoma viral (v-fgr) oncogene homolog	cg14181576	cg26040332	P<0.01	-0.18	P<0.001	-0.12
FILIP1L	filamin A interacting protein 1-like	cg23528247	cg20276377	P<0.001	-0.19	P<0.001	-0.17
GAS7	growth arrest-specific 7	cg06233815	cg25116216	P<0.001	-0.19	P<0.05	-0.07
GNAS	GNAS complex locus	cg02890368	cg25130962	P<0.05	0.14	P<0.01	-0.11
GRASP	GRP1 (general receptor for phosphoinositides 1)-associated scaffold protein	cg06611426	cg00817367	P<0.01	-0.15	P<0.01	-0.10
GSTM1	glutathione S-transferase mu 1	cg24506221		P<0.001	0.15	P<0.001	-0.15
HCK	hemopoietic cell kinase	cg17326769	cg00992385	P<0.01	-0.12	P<0.001	-0.11
HHLA1	HERV-H LTR-associating 1	cg17635970		P<0.05	-0.13	P<0.05	-0.14
IL32	interleukin 32	cg16730716	cg00239353	P<0.001	0.15	P<0.05	-0.15
ITPKB	inositol-trisphosphate 3-kinase B	cg03257293		P<0.01	0.16	P<0.001	-0.13
KCNE1	potassium voltage-gated channel, Isk-related family, member 1	cg03801286	cg14535332	P<0.05	-0.14	P<0.01	-0.15
KCNH5	potassium voltage-gated channel, subfamily H (eag-related), member 5	cg07092725		P<0.001	-0.15	P<0.05	-0.12
KCTD8	potassium channel tetramerisation domain containing 8	cg17790605	cg14677681	P<0.05	-0.10	P<0.001	-0.11
LDHC	lactate dehydrogenase C	cg07093428		P<0.001	0.13	P<0.05	0.08
LDLRAD4	low density lipoprotein receptor class A domain containing 4	cg25140190	cg21349637	P<0.001	-0.19	P<0.001	-0.13
LMO2	LIM domain only 2 (rhombotin-like 1)	cg13100962	cg07132633	P<0.05	-0.14	P<0.01	-0.07
LOC338817	uncharacterized LOC338817	cg18232235		P<0.05	0.11	P<0.001	0.26
MIR140	microRNA 140	cg04703221	cg01735503	P<0.01	-0.16	P<0.05	-0.12
MIR1914	microRNA 1914	cg01966791	cg07135405	P<0.05	-0.15	P<0.05	-0.15
MIR376C	microRNA 376c	cg19653246		P<0.01	-0.16	P<0.001	-0.23
MMP27	matrix metallopeptidase 27	cg10306192		P<0.001	-0.19	P<0.001	-0.17
MSRB3	methionine sulfoxide reductase B3	cg12488187	cg08636169	P<0.001	-0.16	P<0.05	-0.09
OR10K1	olfactory receptor, family 10, subfamily K, member 1	cg01463139		P<0.05	0.13	P<0.001	0.30
OR2A5	olfactory receptor, family 2, subfamily A, member 5	cg18845598		P<0.001	-0.45	P<0.001	0.43
OR4D1	olfactory receptor, family 4, subfamily D, member 1	cg11189272		P<0.001	0.17	P<0.05	0.10
OR52M1	olfactory receptor, family 52, subfamily M, member 1	cg17040924		P<0.001	0.29	P<0.001	-0.29
OSBPL9	oxysterol binding protein-like 9	cg10701801		P<0.05	-0.13	P<0.01	0.16
PHLDB3	pleckstrin homology-like domain, family B, member 3	cg17121205	cg07504984	P<0.05	-0.11	P<0.001	-0.13
PNMAL1	paraneoplastic Ma antigen family-like 1	cg06851207	cg10788735	P<0.05	0.16	P<0.001	-0.10
PRKAR1B	protein kinase, cAMP-dependent, regulatory, type I, beta	cg17593625	cg20381963	P<0.05	-0.14	P<0.05	-0.08
PSORS1C1	psoriasis susceptibility 1 candidate 1	cg24926791		P<0.01	0.16	P<0.05	0.16
RAB37	RAB37, member RAS oncogene family	cg03469804	cg18493981	P<0.05	-0.14	P<0.001	-0.12
RASSF1	Ras association (RalGDS/AF-6) domain family member 1	cg24049629	cg02930432	P<0.001	-0.20	P<0.01	-0.11
RNF219	ring finger protein 219	cg07926092		P<0.001	-0.21	P<0.001	-0.23
RNF5P1	ring finger protein 5, E3 ubiquitin protein ligase pseudogene 1	cg07482220		P<0.01	0.10	P<0.001	-0.12
RUNX3	runt-related transcription factor 3	cg15712177	cg26421310	P<0.01	-0.09	P<0.001	-0.12
SERPINA9	serpin peptidase inhibitor, clade A (alpha-1 antiproteinase, antitrypsin), member 9	cg13251750		P<0.05	-0.14	P<0.001	-0.31
SLC44A2	solute carrier family 44, member 2	cg26847100	cg05567294	P<0.05	-0.15	P<0.05	-0.10
SLC6A1	solute carrier family 6, member 17	cg16164276	cg13048512	P<0.05	0.09	P<0.01	-0.09
SLC9A7P1	solute carrier family 9, subfamily A (NHE7, cation proton antiporter 7), member 7 pseudogene 1	cg24471210	cg25150519	P<0.05	-0.15	P<0.05	-0.08
SLN	sarcolipin	cg24307368		P<0.05	0.14	P<0.01	0.18
ST8SIA2	ST8 alpha-N-acetyl-neuraminide alpha-2,8-sialyltransferase 2	cg08152839	cg24342409	P<0.001	-0.23	P<0.001	-0.13
SULT1A1	sulfotransferase family, cytosolic, 1A, phenol-preferring, member 1	cg05845592		P<0.01	-0.10	P<0.001	-0.13
SUN1	Sad1 and UNC84 domain containing 1	cg05357209		P<0.001	0.20	P<0.01	-0.17
TICAM2	toll-like receptor adaptor molecule 2	cg15395441	cg01554060	P<0.05	-0.13	P<0.001	-0.11
TMEM169	transmembrane protein 169	cg20968678	cg16925210	P<0.01	0.16	P<0.05	-0.07
TRIT1	tRNA isopentenyltransferase 1	cg06717841		P<0.05	0.11	P<0.05	-0.12
TSPYL5	TSPY-like 5	cg09503853	cg04917181	P<0.05	0.14	P<0.05	-0.10
WDR36	WD repeat domain 36	cg11585022		P<0.001	-0.20	P<0.001	0.20
ZNF385A	zinc finger protein 385A	cg09457245	cg20967139	P<0.05	-0.13	P<0.05	-0.07
ZNF681	zinc finger protein 681	cg25958450	cg01615818	P<0.001	-0.19	P<0.001	-0.14
ZSCAN18	zinc finger and SCAN domain containing 18	cg02348449	cg18428688	P<0.01	-0.16	P<0.001	-0.11
ZXDC	ZXD family zinc finger C	cg16898124		P<0.05	-0.10	P<0.05	0.10

The three CpG sites of this region were analyzed by pyrosequencing. On average, all of the CpG sites have lower methylation in the women CPA group with CG1 showing a significant difference at P<0.05 in all the techniques used: MeDIP microarray, Illumina 450K array and pyrosequencing (Figure 2).

The Illumina 450K profiles also include methylation levels for many CpG sites outside of promoters of which 5898 CpG sites are differentially methylated in women with CPA and 5795 are differentially methylated in men with CPA (P<0.05, no correction for multiple testing, Spreadsheet S3). These two sets overlap on a significant 744 CpG sites (P<0.0001; Fisher’s exact test).

## Discussion

We have previously hypothesized that epigenetic mechanisms mediate aggressive behaviors by showing a signature of aggression in the T cells of adult, human males. In this study, we tested the hypothesis that a similar DNA methylation signature exists in women as well. Our study confirms this hypothesis and furthermore identifies a significant but not perfect overlap between the male and female signatures. Indeed, the methylation of 31 gene promoters was shown to associate with physical aggression in both sexes (Table 3). Interestingly, a significant portion of the overlap is due to identical genomic sites being differentially methylated in a gender-independent fashion. Adding to this list, 744 CpG sites were found differentially methylated in men and women with CPA using Illumina 450K arrays. The fact that such an overlap is observed between men and women data in spite of the fact that the methylome analyses were performed separately and independently makes it highly unlikely that the methylome signatures identified here could be derived randomly. The almost perfect overlap between functional categories represented by both the male and female signatures provides further evidence that these signatures are associated with female and male chronic physical aggression (Table 4). The existence of sex-specific and sex-independent components of the signatures is consistent with what we know about sex differences and similarities in human physical aggression, and future studies will examine the relationships of these components with gender-specific and independent aggressive behaviors.

MeDIP microarray and Illumina 450K array methods captured different population of the differentially methylated regions although both methods validated the difference between the groups. These methods measure DNA methylation differently and have different representation of CpG sites in their array. The main difference between the methods is that the MeDIP microarray method measures average differences across 250 bp and their proximal regions while Illumina 450K arrays measure CpG sites independently. One example of the different results obtained from the two techniques is illustrated in Figure 2. *ZNF366* gene promoter was found differentially methylated by both techniques in both sexes but at different CpG sites. Indeed, this region contain CpG sites called differentially methylated by both techniques (Figure 2 on the left highlighted in pink) as well as regions called differentially methylated by MeDIP microarray in both sexes (Figure 2, on the right highlighted in blue and in pink) but where no CpG sites were analyzed by Illumina 450K arrays. It is therefore not surprising that these methods reveal different sensitivities to differences in DNA methylation in different promoters and therefore capture different although somewhat overlapping domains of the differentially methylated landscape associated to chronic aggression.

Three genes of the 430-gene female signature were already known to associate with aggression in animals and in humans (Table 2). Indeed, recent studies that aimed to decipher the biological mechanisms involved in the development and maintenance of chronic aggression have consistently found a key role for the serotonin (5-hydroxytryptamine, 5-HT) pathway, suggesting lower serotoninergic activity associated with aggression/impulsivity [51]–[54]. Tryptophan Hydroxylase is the rate-limiting enzyme in the synthesis of serotonin. In humans, Perez-Rodriguez et al. [55] tested several polymorphisms of *TPH2* and found an extended haplotype associated with enhanced aggressiveness, suicidal behavior, and susceptibility to borderline personality disorder. We found that the promoter of *TPH2* is less methylated in CPA women. We also found *CRHBP*, regulator of cortisol levels, and the glucocorticoid receptor (*NR3C1, GR*) to be less methylated in the CPA women. The glucocorticoid receptor, whose epigenetic changes, were associated with early-life stress (reduced maternal care in rats [31], [56], childhood abuse in human brain [37]), plays also a key role in HPA axis related stress reactivity and aggressive behavior in pigs [57]. The stress response and the HPA axis have also been associated with aggression [51], [58]. Both hyper- and hypo-active HPA axis are associated with aggression in humans [59]–[62]. It is perhaps not surprising that these genes are differentially methylated only in women where the HPA negative feedback control is known to be more sensitive than in males [63]. The same is true for rodents where it has been shown that female rodents have higher HPA axis activity under basal and stress-induced conditions than males [64].

As previously observed in men (Provençal et al., under review in PLoS One), several of the genes in the female aggression signature play roles in cytokine function and inflammatory response (Table 4 and Table S1–3). Recent studies have shown that cytokines are associated with various behavioral disorders such as anxiety, depression, suicide, childhood mood disorder and post-traumatic stress disorder (PTSD) [40], [65]–[75]. It was also suggested that cytokines might play a role in the neurobiology of aggression since they are expressed in brain regions already known to be involved in aggression and behavior [76]–[78]. For example, *IL1RN* have been shown to be involved in defensive aggression through its activation by *IL-1 beta* [79], [80]. Our results show that *IL1RN* belongs to both the male and female aggression signatures. Moreover, cytokines expression in blood and gene methylation in T cells was recently found to associate with men CPA in the same subjects as the one studied here [47]. Further work is needed to investigate the role of peripheral cytokines in aggression but taken together these data suggest that cytokine regulation could play an important role in human behavior and mental health.

Three of the genes in both the male and female signatures of aggression are members of the protocadherin gene family (*PCDH8, PCDHB2, PCDHB8*). Interestingly, we have recently shown that the protocadherin gene family is differentially methylated in hippocampi from rats exposed to low maternal care and humans subjected to child abuse [31], [81]. It is noteworthy that although these genes are clearly involved in brain function, we also observed changes in DNA methylation in T cells associated with aggressive behavior. Similar results were obtained when we recently compared rhesus monkey DNA methylation changes in prefrontal cortex and T cells in response to differences in maternal rearing [32]. This analysis revealed both tissue specific alterations as well as common differentially methylated regions in T cells and prefrontal cortex. Two previous reports suggested that brain function-specific genes were differentially methylated in peripheral blood cells in association with physical aggression [42], [47]. Further work is needed to understand why seemingly brain-specific genes would be differentially methylated in blood DNA in association with aggression and other environmental factors.

There are three important limitations to this study. First, although we recruited subjects from a large longitudinal study, we managed to assess only a small number of women with chronic physical aggression because they represent a very small percentage of the population and are difficult to recruit. Thus, replications are needed from similar longitudinal studies to confirm our results. Second, we analyzed DNA methylation profiles only in adulthood because T cells were not collected at earlier time points during the longitudinal study. New longitudinal studies are needed to help distinguish whether the observed DNA methylation profiles are present before the start of the chronic physical aggression trajectories or are an outcome of these behaviors. Third, there was no psychometric-physical evaluation at the time of blood draw. The acute psychological and/or physical status might confound our findings. Future longitudinal studies that include concurrent blood draws and psychometric-physical evaluations are required to address this question. In addition, childhood abuse is also known to increase the risk of aggression in adolescents and adults [82] and was also found to associate with DNA methylation differences [37]. Therefore, it is possible that child abuse acts as a third factor in explaining the reported association between aggression and DNA methylation. This however might be reflecting the simple fact that these behaviors are molecularly and functionally linked within the same biological pathways.

Nevertheless, this study is consistent with a DNA methylation signature of chronic aggression that is maintained into adulthood and provides justification for future longitudinal and intervention studies using T cell methylomes to investigate the causal and temporal relationships between social experiences and long-term behavioral phenotypes in humans.

## Supporting Information

Figure S1 **Distributions of promoter methylation levels by published expression level.** Genes expression levels were obtained from publicly available T cells expression profiles (Su, Wiltshire et al. 2004) that we used to partition genes by expression percentiles (0–5, 5–10, …, 95–100). To obtain gene promoter methylation levels, we computed the average normalized intensity of each probe across all samples and replicates, and then applied the Bayesian deconvolution algorithm mentioned above to the resulting averages (Down, Rakyan et al. 2008). Shown are the distributions of methylation levels for each expression percentile. The distributions show that genes with low or no expression (represented in green) tend to have highly methylated promoters, whereas genes with high expression (represented in red) tend to have lower promoter methylation.
(TIF)

Figure S2 **Validation by Q-MeDIP of differentially methylated sequences between CPA (n = 5) and NPA (n = 14) groups. A.** Fold differences between CPA and NPA groups obtain by Q-MeDIP (grey fill) and microarray (dark fill) analyses are shown for 19 genes predicted to be more methylated (n = 2) and less methylated (n = 17) in the CPA individuals by the microarray analysis. In bold are the genes in common between men and women MeDIP-arrays. * and # indicated the *P* value obtained by comparing the groups (# *P*≤0.1; * *P*<0.05; ** *P<*0.001; *** *P*<0.0001). **B.** Correlation of the fold differences between CPA and NPA obtain by Q-MeDIP and by MeDIP-array for 19 genes predicted to be differentially methylated between CPA and NPA groups by the microarray analysis.
(TIF)

Table S1 Top 25 gene promoters differentially methylated between women CPA (n = 5) and NPA (n = 14) groups from the MeDIP-microarray analysis (P<0.005 and FDR <0.01). In bold are genes also found to be differentially methylated in men MeDIP-microarray analysis. Underlined are genes validated by Illumina 450K arrays.
(DOCX)

Table S2 Biological functions enriched with genes whose methylation is associated with women aggression from IPA analysis (n = 430 genes).
(DOCX)

Table S3 Canonical pathways enriched with genes whose methylation is associated with women aggression from IPA analysis (n = 430 genes). All of the p values were calculated using a right tailed Fisher’s exact test and corrected for multiple comparison with the Benjamini-Hochberg method. Significance threshold were p = 0.05.
(DOCX)

Table S4 Upstream regulators showing a significant overlap with genes whose methylation is associated with aggression from IPA analysis (n = 430 genes). Upstream regulators differentially methylated between chronic and normal aggression are shown in bold. Significance threshold were P = 0.05.
(DOCX)

Table S5 Biological functions enriched with genes whose methylation is associated with aggression in both sexes from IPA analysis (women n = 430 genes and men n = 448 genes).
(DOCX)

Table S6 Canonical pathway enriched with genes whose methylation is associated with aggression in both sexes from IPA analysis (women n = 430 genes and men n = 448 genes).
(DOCX)

Table S7 Upstream regulators showing a significant overlap with genes whose methylation is associated with aggression in both sexes from IPA analysis (women n = 430 genes and men n = 448 genes).
(DOCX)

Table S8 Gene promoters with significant changes in methylation between women CPA and NPA groups from both MeDIP-microarrays and Illumina 450k arrays analysis.
(DOCX)

Methods S1 (DOCX)

Spreadsheet S1 **Full table of probes differentially methylated between women CPA and women NPA by MeDIP-microarray.**
(XLSX)

Spreadsheet S2 **Full table of the 716 gene promoter CGs differentially methylated between women CPA and women NPA by Illumina 450K (CPA-NPA).**
(XLSX)

Spreadsheet S3 **Full table of all the CG sites differentially methylated between CPA and NPA for each sex by Illumina 450K (CPA-NPA).** Sheet 1 corresponds to women CPA-NPA results. Sheet 2 is men CPA-NPA results.
(XLSX)
