# Supplementary figures and images for: DNA Methylation Signature of Childhood Chronic Physical Aggression in T Cells of Both Men and Women
Source: PLoS One. 2014 Jan 24;9(1):e86822. doi: 10.1371/journal.pone.0086822 (PMC3901708; doi:10.1371/journal.pone.0086822)

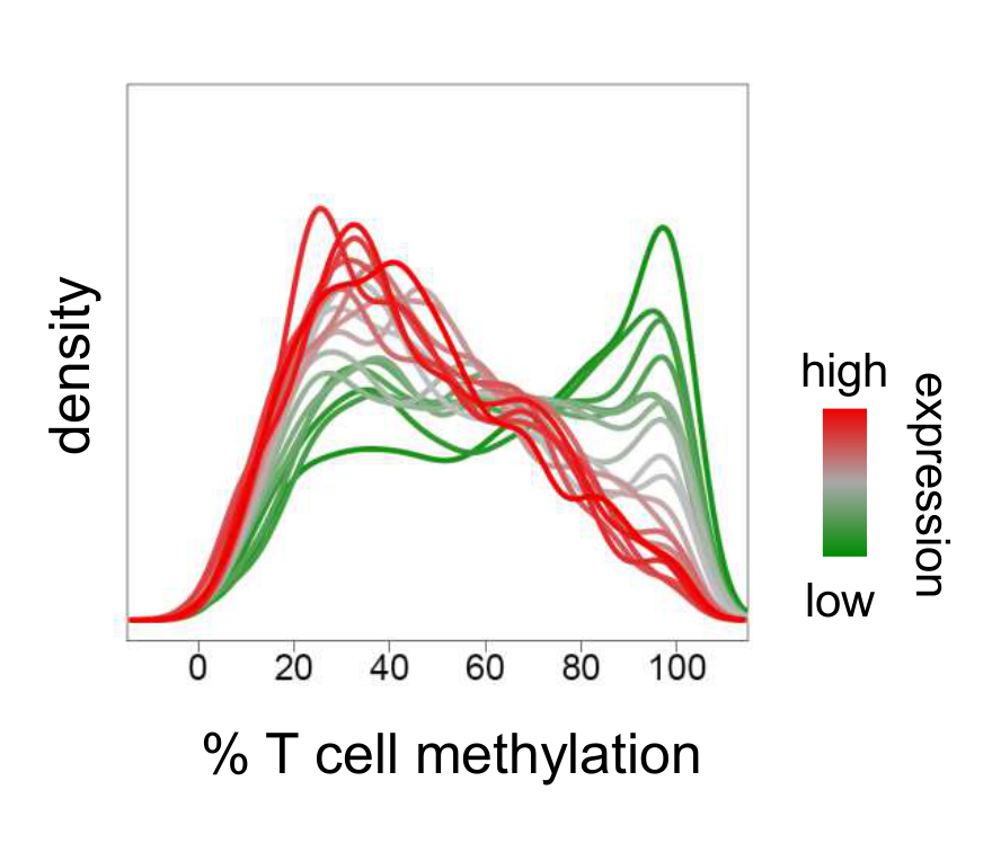

Supplement: Figure S1 — Distributions of promoter methylation levels by published expression level. Genes expression levels were obtained from publicly available T cells expression profiles (Su, Wiltshire et al. 2004) that we used to partition genes by expression percentiles (0–5, 5–10, …, 95–100). To obtain gene promoter methylation levels, we computed the average normalized intensity of each probe across all samples and replicates, and then applied the Bayesian deconvolution algorithm mentioned above to the resulting averages (Down, Rakyan et al. 2008). Shown are the distributions of methylation levels for each expression percentile. The distributions show that genes with low or no expression (represented in green) tend to have highly methylated promoters, whereas genes with high expression (represented in red) tend to have lower promoter methylation. (TIF) [file pone.0086822.s001.tif]
