# Supplementary material for: DNA Methylation Signature of Childhood Chronic Physical Aggression in T Cells of Both Men and Women
Source: PLoS One. 2014 Jan 24;9(1):e86822. doi: 10.1371/journal.pone.0086822 (PMC3901708; doi:10.1371/journal.pone.0086822)
Supplement: Table S1 — Top 25 gene promoters differentially methylated between women CPA (n = 5) and NPA (n = 14) groups from the MeDIP-microarray analysis (P<0.005 and FDR <0.01). In bold are genes also found to be differentially methylated in men MeDIP-microarray analysis. Underlined are genes validated by Illumina 450K arrays. (DOCX) [file pone.0086822.s003.docx]

**Supplementary Table S1. Top 25 gene promoters differentially methylated between women CPA (n=5) and NPA (n=14) groups from the MeDIP-microarray analysis (P < 0.005 and FDR < 0.01).** In bold are genes also found to be differentially methylated in men MeDIP-microarray analysis. Underlined are genes validated by Illumina 450K arrays.

| **Gene name** | **P value** | **FDR** | **More methylated in** | **Full name** | **Gene details** |
| --- | --- | --- | --- | --- | --- |
| ANKRD22 | 0.001 | 0.007 | NPA | ankyrin repeat domain 22 |  |
| ATP11C | 0.002 | 0.0007 | NPA | ATPase, class VI, type 11C | ATPase |
| CCL3 | 0.001 | 0.0003 | NPA | chemokine (C-C motif) ligand 3 | Small inducible cytokine with inflammatory and chemokinetic properties |
| CYP27B1 | 0.001 | 0.004 | NPA | cytochrome P450, family 27, subfamily B, polypeptide 1 | Mitochondrial enzyme important for calcium homeostasis and vitamin D level |
| FCAMR | 0.002 | 0.0001 | NPA | Fc receptor, IgA, IgM, high affinity | Receptor for the Fc fragment of IgA and IgM |
| FGA | 0.00002 | 0.003 | NPA | Fibrinogen Alpha Chain | Component of fibrinogen |
| FXYD2 | 0.0005 | 0.009 | NPA | FXYD domain containing ion transport regulator 2 | Sodium/potassium-transporting ATPase, member of the FXYD family of transmembrane protein |
| GLIPR1L1 | 0.0004 | 0.003 | NPA | GLI pathogenesis-related 1 like 1 | Function is yet unknown |
| GNG4 | 0.0002 | 0.001 | NPA | guanine nucleotide binding protein (G protein), gamma 4 | Membrane bound GTPase |
| GPR21 | 0.0004 | 0.004 | NPA | G protein-coupled receptor 21 | Orphan receptor |
| **GPR84** | 0.001 | 0.003 | NPA | G protein-coupled receptor 84 | Inflammation-related G-protein coupled receptor |
| IL1F10 | 0.001 | 0.009 | NPA | interleukin 1 family, member 10 (theta) | Regulate adapted and innate immune responses |
| LMO2 | 0.002 | 0.002 | NPA | LIM domain only 2 (rhombotin-like 1) | Regulate red blood cell development |
| **MGP** | 0.001 | 0.0004 | NPA | matrix Gla protein | Inhibitor of bone formation |
| MUCL1 | 0.001 | 0.002 | NPA | mucin-like 1 | function is yet unknown |
| PBXIP1 | 0.002 | 0.006 | NPA | pre-B-cell leukemia homeobox interacting protein 1 | Regulator of pre-B-cell leukemia transcription factors (BPXs) function. influence estrogen receptors-alpha signaling |
| POU2AF1 | 0.001 | 0.002 | CPA | POU class 2 associating factor 1 | Transcriptional coactivator essential for the response of B-cells to antigens |
| PPP1R1C | 0.0002 | 0.007 | NPA | protein phosphatase 1, regulatory (inhibitor) subunit 1C | Major serine/threonine phosphatase. Promotes cell growth and cell cycle progress at the G1/S transition |
| PRDM1 | 0.0002 | 0.004 | CPA | PR domain containing 1, with ZNF domain | Drives the maturation of B-lymphocytes into Ig secreting cells. repressor of beta-interferon gene expression |
| RHOH | 0.002 | 0.0005 | CPA | ras homolog family member H | Ras superfamily of small GTPases transcribed only in hemopoietic cells. |
| SH3BGRL3 | 0.003 | 0.009 | NPA | SH3 domain binding glutamic acid-rich protein like 3 | Could act as a modulator of glutaredoxin biological activity |
| SMIM3 | 0.001 | 0.006 | NPA | small integral membrane protein 3 | Small integral membrane protein 3 |
| TMPRSS5 | 0.001 | 0.003 | NPA | transmembrane protease, serine 5 | Sserine protease family |
| TNFAIP6 | 0.0003 | 0.004 | NPA | tumor necrosis factor, alpha-induced protein 6 | Induced by proinflammatory cytokines such as TNFα and IL-1. |
| **ZNF366** | 0.003 | 0.0004 | NPA | zinc finger protein 366 | Transcriptional repression activity. estrogen receptor corepressor that acts through CtBP and histone deacetylases |
