# Supplementary material for: DNA Methylation Signature of Childhood Chronic Physical Aggression in T Cells of Both Men and Women
Source: PLoS One. 2014 Jan 24;9(1):e86822. doi: 10.1371/journal.pone.0086822 (PMC3901708; doi:10.1371/journal.pone.0086822)
Supplement: Table S2 — Biological functions enriched with genes whose methylation is associated with women aggression from IPA analysis (n = 430 genes). (DOCX) [file pone.0086822.s004.docx]

**Supplementary Table S2. Biological functions enriched with genes whose methylation is associated with women aggression from IPA analysis (n=430 genes).**

| **Category** | **p-Value** | **p-value max** | **# Molecules** |
| --- | --- | --- | --- |
| Amino Acid Metabolism | 3.69E-04 | 3.17E-03 | 2 |
| Antimicrobial Response | 8.06E-05 | 3.55E-03 | 5 |
| Behavior | 8.62E-04 |  | 5 |
| Behavior | 2.16E-03 |  | 13 |
| Cancer | 2.47E-09 |  | 221 |
| Cancer | 8.68E-09 |  | 150 |
| Cancer | 7.67E-08 |  | 190 |
| Cancer | 1.05E-07 |  | 188 |
| Cancer | 1.24E-07 |  | 191 |
| Cancer | 5.27E-06 |  | 126 |
| Cancer | 1.04E-05 |  | 122 |
| Cancer | 2.44E-05 |  | 114 |
| Cancer | 3.13E-04 |  | 20 |
| Cancer | 6.22E-04 |  | 4 |
| Cancer | 1.08E-03 |  | 13 |
| Cancer | 2.75E-03 |  | 9 |
| Cancer | 3.72E-03 |  | 16 |
| Carbohydrate Metabolism | 6.97E-04 |  | 7 |
| Carbohydrate Metabolism | 1.82E-03 |  | 8 |
| Carbohydrate Metabolism | 1.91E-03 |  | 4 |
| Carbohydrate Metabolism | 2.16E-03 |  | 2 |
| Carbohydrate Metabolism | 2.20E-03 |  | 3 |
| Carbohydrate Metabolism | 2.40E-03 |  | 10 |
| Carbohydrate Metabolism | 4.37E-03 |  | 9 |
| Cardiovascular Disease | 2.63E-04 |  | 4 |
| Cardiovascular Disease | 8.07E-04 |  | 34 |
| Cardiovascular Disease | 1.09E-03 |  | 2 |
| Cardiovascular Disease | 1.09E-03 |  | 2 |
| Cardiovascular Disease | 1.67E-03 |  | 5 |
| Cardiovascular Disease | 2.16E-03 |  | 2 |
| Cardiovascular Disease | 2.16E-03 |  | 2 |
| Cardiovascular Disease | 3.55E-03 |  | 4 |
| Cardiovascular Disease | 4.04E-03 |  | 9 |
| Cardiovascular System Development and Function | 2.10E-04 |  | 11 |
| Cardiovascular System Development and Function | 4.09E-04 |  | 16 |
| Cardiovascular System Development and Function | 5.08E-04 |  | 15 |
| Cardiovascular System Development and Function | 5.77E-04 |  | 6 |
| Cardiovascular System Development and Function | 6.57E-04 |  | 30 |
| Cell Cycle | 3.55E-03 |  | 2 |
| Cell Death and Survival | 8.87E-06 |  | 4 |
| Cell Death and Survival | 1.19E-05 |  | 17 |
| Cell Death and Survival | 2.52E-05 |  | 96 |
| Cell Death and Survival | 2.67E-05 |  | 8 |
| Cell Death and Survival | 5.76E-05 |  | 118 |
| Cell Death and Survival | 6.86E-05 |  | 3 |
| Cell Death and Survival | 9.07E-05 |  | 11 |
| Cell Death and Survival | 9.72E-05 |  | 9 |
| Cell Death and Survival | 1.23E-04 |  | 14 |
| Cell Death and Survival | 1.27E-04 |  | 15 |
| Cell Death and Survival | 1.35E-04 |  | 3 |
| Cell Death and Survival | 1.62E-04 |  | 14 |
| Cell Death and Survival | 1.64E-04 |  | 7 |
| Cell Death and Survival | 1.64E-04 |  | 7 |
| Cell Death and Survival | 2.04E-04 |  | 4 |
| Cell Death and Survival | 2.38E-04 |  | 6 |
| Cell Death and Survival | 3.68E-04 |  | 8 |
| Cell Death and Survival | 3.69E-04 |  | 2 |
| Cell Death and Survival | 3.69E-04 |  | 2 |
| Cell Death and Survival | 6.22E-04 |  | 4 |
| Cell Death and Survival | 6.37E-04 |  | 6 |
| Cell Death and Survival | 8.63E-04 |  | 13 |
| Cell Death and Survival | 1.09E-03 |  | 2 |
| Cell Death and Survival | 1.09E-03 |  | 2 |
| Cell Death and Survival | 1.09E-03 |  | 2 |
| Cell Death and Survival | 1.09E-03 |  | 2 |
| Cell Death and Survival | 1.09E-03 |  | 2 |
| Cell Death and Survival | 1.24E-03 |  | 4 |
| Cell Death and Survival | 1.35E-03 |  | 5 |
| Cell Death and Survival | 1.37E-03 |  | 3 |
| Cell Death and Survival | 1.89E-03 |  | 30 |
| Cell Death and Survival | 1.91E-03 |  | 4 |
| Cell Death and Survival | 2.04E-03 |  | 29 |
| Cell Death and Survival | 2.16E-03 |  | 2 |
| Cell Death and Survival | 2.16E-03 |  | 2 |
| Cell Death and Survival | 2.28E-03 |  | 23 |
| Cell Death and Survival | 2.30E-03 |  | 11 |
| Cell Death and Survival | 2.49E-03 |  | 4 |
| Cell Death and Survival | 2.53E-03 |  | 7 |
| Cell Death and Survival | 2.61E-03 |  | 6 |
| Cell Death and Survival | 2.91E-03 |  | 90 |
| Cell Death and Survival | 3.55E-03 |  | 2 |
| Cell Death and Survival | 3.55E-03 |  | 2 |
| Cell Death and Survival | 3.93E-03 |  | 3 |
| Cell Death and Survival | 4.06E-03 |  | 5 |
| Cell Death and Survival | 4.16E-03 |  | 6 |
| Cell Death and Survival | 4.35E-03 |  | 21 |
| Cell Morphology | 1.26E-04 |  | 20 |
| Cell Morphology | 1.88E-04 |  | 11 |
| Cell Morphology | 3.26E-04 |  | 22 |
| Cell Morphology | 8.53E-04 |  | 10 |
| Cell Morphology | 1.95E-03 |  | 6 |
| Cell Morphology | 2.16E-03 |  | 2 |
| Cell Morphology | 2.77E-03 |  | 18 |
| Cell Morphology | 3.55E-03 |  | 2 |
| Cell Morphology | 3.55E-03 |  | 2 |
| Cell Morphology | 3.89E-03 |  | 12 |
| Cell Morphology | 3.90E-03 |  | 6 |
| Cell Morphology | 3.93E-03 |  | 3 |
| Cell Morphology | 3.97E-03 |  | 7 |
| Cell Signaling | 2.55E-06 |  | 30 |
| Cell Signaling | 8.19E-05 |  | 22 |
| Cell Signaling | 3.73E-04 |  | 18 |
| Cell Signaling | 6.65E-04 |  | 9 |
| Cell Signaling | 2.00E-03 |  | 12 |
| Cell-mediated Immune Response | 4.50E-06 |  | 4 |
| Cell-mediated Immune Response | 5.20E-05 |  | 27 |
| Cell-mediated Immune Response | 2.26E-04 |  | 25 |
| Cell-mediated Immune Response | 3.69E-04 |  | 2 |
| Cell-mediated Immune Response | 4.15E-04 |  | 4 |
| Cell-mediated Immune Response | 5.24E-04 |  | 9 |
| Cell-mediated Immune Response | 5.68E-04 |  | 19 |
| Cell-mediated Immune Response | 7.91E-04 |  | 8 |
| Cell-mediated Immune Response | 2.07E-03 |  | 12 |
| Cell-mediated Immune Response | 2.61E-03 |  | 13 |
| Cell-mediated Immune Response | 3.90E-03 |  | 6 |
| Cell-mediated Immune Response | 4.29E-03 |  | 10 |
| Cell-To-Cell Signaling and Interaction | 1.20E-12 |  | 32 |
| Cell-To-Cell Signaling and Interaction | 8.40E-12 |  | 28 |
| Cell-To-Cell Signaling and Interaction | 4.77E-11 |  | 23 |
| Cell-To-Cell Signaling and Interaction | 1.37E-10 |  | 46 |
| Cell-To-Cell Signaling and Interaction | 3.95E-10 |  | 49 |
| Cell-To-Cell Signaling and Interaction | 4.45E-10 |  | 59 |
| Cell-To-Cell Signaling and Interaction | 1.46E-09 |  | 22 |
| Cell-To-Cell Signaling and Interaction | 6.64E-09 |  | 31 |
| Cell-To-Cell Signaling and Interaction | 1.37E-08 |  | 16 |
| Cell-To-Cell Signaling and Interaction | 1.63E-08 |  | 15 |
| Cell-To-Cell Signaling and Interaction | 3.02E-08 |  | 19 |
| Cell-To-Cell Signaling and Interaction | 3.35E-08 |  | 13 |
| Cell-To-Cell Signaling and Interaction | 4.24E-08 |  | 15 |
| Cell-To-Cell Signaling and Interaction | 4.42E-08 |  | 16 |
| Cell-To-Cell Signaling and Interaction | 7.69E-08 |  | 15 |
| Cell-To-Cell Signaling and Interaction | 2.86E-07 |  | 14 |
| Cell-To-Cell Signaling and Interaction | 3.73E-07 |  | 17 |
| Cell-To-Cell Signaling and Interaction | 3.94E-07 |  | 26 |
| Cell-To-Cell Signaling and Interaction | 4.91E-07 |  | 12 |
| Cell-To-Cell Signaling and Interaction | 5.27E-07 |  | 16 |
| Cell-To-Cell Signaling and Interaction | 1.14E-06 |  | 32 |
| Cell-To-Cell Signaling and Interaction | 5.09E-06 |  | 29 |
| Cell-To-Cell Signaling and Interaction | 5.58E-06 |  | 18 |
| Cell-To-Cell Signaling and Interaction | 5.92E-06 |  | 16 |
| Cell-To-Cell Signaling and Interaction | 6.23E-06 |  | 11 |
| Cell-To-Cell Signaling and Interaction | 6.42E-06 |  | 19 |
| Cell-To-Cell Signaling and Interaction | 7.43E-06 |  | 12 |
| Cell-To-Cell Signaling and Interaction | 1.94E-05 |  | 17 |
| Cell-To-Cell Signaling and Interaction | 3.70E-05 |  | 16 |
| Cell-To-Cell Signaling and Interaction | 3.89E-05 |  | 12 |
| Cell-To-Cell Signaling and Interaction | 4.03E-05 |  | 11 |
| Cell-To-Cell Signaling and Interaction | 4.10E-05 |  | 26 |
| Cell-To-Cell Signaling and Interaction | 1.23E-04 |  | 14 |
| Cell-To-Cell Signaling and Interaction | 1.87E-04 |  | 12 |
| Cell-To-Cell Signaling and Interaction | 1.94E-04 |  | 9 |
| Cell-To-Cell Signaling and Interaction | 2.04E-04 |  | 4 |
| Cell-To-Cell Signaling and Interaction | 2.11E-04 |  | 73 |
| Cell-To-Cell Signaling and Interaction | 2.38E-04 |  | 6 |
| Cell-To-Cell Signaling and Interaction | 2.40E-04 |  | 9 |
| Cell-To-Cell Signaling and Interaction | 2.54E-04 |  | 12 |
| Cell-To-Cell Signaling and Interaction | 2.68E-04 |  | 6 |
| Cell-To-Cell Signaling and Interaction | 3.78E-04 |  | 6 |
| Cell-To-Cell Signaling and Interaction | 4.87E-04 |  | 17 |
| Cell-To-Cell Signaling and Interaction | 5.02E-04 |  | 16 |
| Cell-To-Cell Signaling and Interaction | 5.11E-04 |  | 4 |
| Cell-To-Cell Signaling and Interaction | 5.91E-04 |  | 18 |
| Cell-To-Cell Signaling and Interaction | 5.92E-04 |  | 5 |
| Cell-To-Cell Signaling and Interaction | 5.94E-04 |  | 7 |
| Cell-To-Cell Signaling and Interaction | 7.53E-04 |  | 7 |
| Cell-To-Cell Signaling and Interaction | 7.72E-04 |  | 6 |
| Cell-To-Cell Signaling and Interaction | 1.04E-03 |  | 3 |
| Cell-To-Cell Signaling and Interaction | 1.06E-03 |  | 4 |
| Cell-To-Cell Signaling and Interaction | 1.09E-03 |  | 2 |
| Cell-To-Cell Signaling and Interaction | 1.24E-03 |  | 4 |
| Cell-To-Cell Signaling and Interaction | 1.28E-03 |  | 9 |
| Cell-To-Cell Signaling and Interaction | 1.29E-03 |  | 13 |
| Cell-To-Cell Signaling and Interaction | 1.34E-03 |  | 65 |
| Cell-To-Cell Signaling and Interaction | 1.37E-03 |  | 3 |
| Cell-To-Cell Signaling and Interaction | 1.50E-03 |  | 5 |
| Cell-To-Cell Signaling and Interaction | 1.75E-03 |  | 7 |
| Cell-To-Cell Signaling and Interaction | 1.78E-03 |  | 18 |
| Cell-To-Cell Signaling and Interaction | 2.16E-03 |  | 2 |
| Cell-To-Cell Signaling and Interaction | 2.44E-03 |  | 5 |
| Cell-To-Cell Signaling and Interaction | 3.30E-03 |  | 10 |
| Cell-To-Cell Signaling and Interaction | 3.55E-03 |  | 2 |
| Cellular Assembly and Organization | 3.69E-04 |  | 2 |
| Cellular Assembly and Organization | 1.09E-03 |  | 2 |
| Cellular Assembly and Organization | 3.55E-03 |  | 2 |
| Cellular Compromise | 1.91E-05 |  | 7 |
| Cellular Compromise | 5.32E-05 |  | 15 |
| Cellular Compromise | 6.96E-05 |  | 9 |
| Cellular Compromise | 1.46E-04 |  | 5 |
| Cellular Compromise | 3.78E-04 |  | 6 |
| Cellular Compromise | 7.34E-04 |  | 10 |
| Cellular Compromise | 7.66E-04 |  | 3 |
| Cellular Compromise | 7.72E-04 |  | 6 |
| Cellular Compromise | 9.28E-04 |  | 6 |
| Cellular Compromise | 1.50E-03 |  | 5 |
| Cellular Compromise | 2.16E-03 |  | 2 |
| Cellular Compromise | 3.55E-03 |  | 4 |
| Cellular Compromise | 3.55E-03 |  | 2 |
| Cellular Compromise | 4.06E-03 |  | 5 |
| Cellular Development | 1.11E-07 |  | 45 |
| Cellular Development | 1.11E-07 |  | 47 |
| Cellular Development | 4.33E-06 |  | 39 |
| Cellular Development | 4.50E-06 |  | 4 |
| Cellular Development | 4.83E-06 |  | 79 |
| Cellular Development | 7.42E-06 |  | 38 |
| Cellular Development | 8.16E-06 |  | 32 |
| Cellular Development | 1.17E-05 |  | 34 |
| Cellular Development | 1.73E-05 |  | 29 |
| Cellular Development | 3.60E-05 |  | 29 |
| Cellular Development | 1.45E-04 |  | 30 |
| Cellular Development | 1.49E-04 |  | 35 |
| Cellular Development | 1.65E-04 |  | 31 |
| Cellular Development | 2.26E-04 |  | 25 |
| Cellular Development | 2.75E-04 |  | 17 |
| Cellular Development | 3.69E-04 |  | 2 |
| Cellular Development | 4.08E-04 |  | 15 |
| Cellular Development | 4.14E-04 |  | 24 |
| Cellular Development | 4.15E-04 |  | 4 |
| Cellular Development | 5.18E-04 |  | 5 |
| Cellular Development | 5.68E-04 |  | 19 |
| Cellular Development | 7.01E-04 |  | 14 |
| Cellular Development | 7.66E-04 |  | 3 |
| Cellular Development | 9.85E-04 |  | 9 |
| Cellular Development | 1.06E-03 |  | 4 |
| Cellular Development | 1.69E-03 |  | 11 |
| Cellular Development | 2.16E-03 |  | 13 |
| Cellular Development | 2.18E-03 |  | 14 |
| Cellular Development | 2.20E-03 |  | 3 |
| Cellular Development | 2.71E-03 |  | 3 |
| Cellular Development | 2.75E-03 |  | 9 |
| Cellular Development | 3.45E-03 |  | 19 |
| Cellular Development | 3.55E-03 |  | 2 |
| Cellular Development | 3.96E-03 |  | 4 |
| Cellular Development | 3.97E-03 |  | 7 |
| Cellular Development | 4.29E-03 |  | 10 |
| Cellular Function and Maintenance | 1.37E-08 |  | 16 |
| Cellular Function and Maintenance | 4.24E-08 |  | 15 |
| Cellular Function and Maintenance | 1.00E-07 |  | 13 |
| Cellular Function and Maintenance | 2.11E-07 |  | 15 |
| Cellular Function and Maintenance | 2.86E-07 |  | 14 |
| Cellular Function and Maintenance | 4.91E-07 |  | 12 |
| Cellular Function and Maintenance | 1.51E-06 |  | 34 |
| Cellular Function and Maintenance | 1.67E-06 |  | 66 |
| Cellular Function and Maintenance | 4.43E-06 |  | 23 |
| Cellular Function and Maintenance | 4.50E-06 |  | 4 |
| Cellular Function and Maintenance | 6.42E-06 |  | 19 |
| Cellular Function and Maintenance | 7.09E-06 |  | 33 |
| Cellular Function and Maintenance | 2.01E-05 |  | 30 |
| Cellular Function and Maintenance | 4.90E-05 |  | 28 |
| Cellular Function and Maintenance | 5.20E-05 |  | 27 |
| Cellular Function and Maintenance | 6.29E-05 |  | 21 |
| Cellular Function and Maintenance | 1.07E-04 |  | 21 |
| Cellular Function and Maintenance | 1.18E-04 |  | 20 |
| Cellular Function and Maintenance | 1.21E-04 |  | 5 |
| Cellular Function and Maintenance | 1.55E-04 |  | 4 |
| Cellular Function and Maintenance | 2.04E-04 |  | 4 |
| Cellular Function and Maintenance | 2.26E-04 |  | 25 |
| Cellular Function and Maintenance | 2.30E-04 |  | 8 |
| Cellular Function and Maintenance | 3.69E-04 |  | 2 |
| Cellular Function and Maintenance | 3.69E-04 |  | 2 |
| Cellular Function and Maintenance | 3.73E-04 |  | 18 |
| Cellular Function and Maintenance | 3.78E-04 |  | 6 |
| Cellular Function and Maintenance | 3.84E-04 |  | 10 |
| Cellular Function and Maintenance | 4.15E-04 |  | 4 |
| Cellular Function and Maintenance | 4.69E-04 |  | 6 |
| Cellular Function and Maintenance | 5.68E-04 |  | 19 |
| Cellular Function and Maintenance | 6.22E-04 |  | 4 |
| Cellular Function and Maintenance | 6.65E-04 |  | 9 |
| Cellular Function and Maintenance | 7.72E-04 |  | 6 |
| Cellular Function and Maintenance | 8.62E-04 |  | 5 |
| Cellular Function and Maintenance | 9.01E-04 |  | 14 |
| Cellular Function and Maintenance | 1.01E-03 |  | 15 |
| Cellular Function and Maintenance | 1.09E-03 |  | 2 |
| Cellular Function and Maintenance | 1.21E-03 |  | 6 |
| Cellular Function and Maintenance | 1.50E-03 |  | 5 |
| Cellular Function and Maintenance | 2.03E-03 |  | 5 |
| Cellular Function and Maintenance | 2.20E-03 |  | 3 |
| Cellular Function and Maintenance | 2.21E-03 |  | 10 |
| Cellular Function and Maintenance | 2.44E-03 |  | 5 |
| Cellular Function and Maintenance | 2.61E-03 |  | 6 |
| Cellular Function and Maintenance | 2.81E-03 |  | 4 |
| Cellular Function and Maintenance | 3.55E-03 |  | 2 |
| Cellular Function and Maintenance | 3.55E-03 |  | 2 |
| Cellular Function and Maintenance | 3.70E-03 |  | 10 |
| Cellular Function and Maintenance | 3.90E-03 |  | 6 |
| Cellular Function and Maintenance | 4.29E-03 |  | 10 |
| Cellular Growth and Proliferation | 1.11E-07 |  | 45 |
| Cellular Growth and Proliferation | 1.11E-07 |  | 47 |
| Cellular Growth and Proliferation | 4.33E-06 |  | 39 |
| Cellular Growth and Proliferation | 7.42E-06 |  | 38 |
| Cellular Growth and Proliferation | 2.81E-05 |  | 124 |
| Cellular Growth and Proliferation | 1.12E-04 |  | 15 |
| Cellular Growth and Proliferation | 1.45E-04 |  | 30 |
| Cellular Growth and Proliferation | 2.75E-04 |  | 17 |
| Cellular Growth and Proliferation | 3.56E-04 |  | 7 |
| Cellular Growth and Proliferation | 3.69E-04 |  | 2 |
| Cellular Growth and Proliferation | 4.08E-04 |  | 15 |
| Cellular Growth and Proliferation | 7.01E-04 |  | 14 |
| Cellular Growth and Proliferation | 1.25E-03 |  | 22 |
| Cellular Growth and Proliferation | 1.44E-03 |  | 23 |
| Cellular Growth and Proliferation | 1.67E-03 |  | 4 |
| Cellular Growth and Proliferation | 1.75E-03 |  | 7 |
| Cellular Growth and Proliferation | 1.95E-03 |  | 6 |
| Cellular Growth and Proliferation | 2.19E-03 |  | 4 |
| Cellular Growth and Proliferation | 2.75E-03 |  | 9 |
| Cellular Growth and Proliferation | 2.77E-03 |  | 14 |
| Cellular Growth and Proliferation | 3.28E-03 |  | 3 |
| Cellular Growth and Proliferation | 3.30E-03 |  | 10 |
| Cellular Growth and Proliferation | 3.55E-03 |  | 2 |
| Cellular Growth and Proliferation | 3.93E-03 |  | 3 |
| Cellular Growth and Proliferation | 3.96E-03 |  | 4 |
| Cellular Growth and Proliferation | 3.97E-03 |  | 7 |
| Cellular Movement | 1.08E-11 |  | 53 |
| Cellular Movement | 2.83E-11 |  | 42 |
| Cellular Movement | 1.93E-10 |  | 40 |
| Cellular Movement | 6.11E-10 |  | 27 |
| Cellular Movement | 6.46E-10 |  | 54 |
| Cellular Movement | 1.43E-09 |  | 26 |
| Cellular Movement | 2.47E-09 |  | 85 |
| Cellular Movement | 3.18E-09 |  | 30 |
| Cellular Movement | 3.64E-09 |  | 29 |
| Cellular Movement | 3.71E-09 |  | 30 |
| Cellular Movement | 8.08E-09 |  | 20 |
| Cellular Movement | 1.08E-08 |  | 90 |
| Cellular Movement | 5.17E-08 |  | 34 |
| Cellular Movement | 5.46E-08 |  | 24 |
| Cellular Movement | 7.89E-08 |  | 35 |
| Cellular Movement | 1.00E-07 |  | 13 |
| Cellular Movement | 3.91E-07 |  | 16 |
| Cellular Movement | 5.31E-07 |  | 15 |
| Cellular Movement | 5.67E-07 |  | 16 |
| Cellular Movement | 2.88E-06 |  | 16 |
| Cellular Movement | 3.19E-06 |  | 23 |
| Cellular Movement | 3.87E-06 |  | 17 |
| Cellular Movement | 1.28E-05 |  | 27 |
| Cellular Movement | 1.50E-05 |  | 9 |
| Cellular Movement | 2.39E-05 |  | 5 |
| Cellular Movement | 2.40E-05 |  | 23 |
| Cellular Movement | 4.12E-05 |  | 10 |
| Cellular Movement | 4.41E-05 |  | 18 |
| Cellular Movement | 1.03E-04 |  | 12 |
| Cellular Movement | 1.07E-04 |  | 6 |
| Cellular Movement | 1.15E-04 |  | 12 |
| Cellular Movement | 1.16E-04 |  | 4 |
| Cellular Movement | 1.16E-04 |  | 4 |
| Cellular Movement | 1.21E-04 |  | 5 |
| Cellular Movement | 1.23E-04 |  | 14 |
| Cellular Movement | 1.99E-04 |  | 11 |
| Cellular Movement | 2.46E-04 |  | 5 |
| Cellular Movement | 2.54E-04 |  | 12 |
| Cellular Movement | 2.68E-04 |  | 6 |
| Cellular Movement | 3.08E-04 |  | 11 |
| Cellular Movement | 3.68E-04 |  | 3 |
| Cellular Movement | 3.69E-04 |  | 2 |
| Cellular Movement | 4.37E-04 |  | 13 |
| Cellular Movement | 4.87E-04 |  | 17 |
| Cellular Movement | 5.11E-04 |  | 4 |
| Cellular Movement | 5.24E-04 |  | 9 |
| Cellular Movement | 5.44E-04 |  | 3 |
| Cellular Movement | 5.91E-04 |  | 18 |
| Cellular Movement | 5.92E-04 |  | 5 |
| Cellular Movement | 7.02E-04 |  | 6 |
| Cellular Movement | 7.91E-04 |  | 8 |
| Cellular Movement | 1.17E-03 |  | 7 |
| Cellular Movement | 1.24E-03 |  | 4 |
| Cellular Movement | 1.29E-03 |  | 13 |
| Cellular Movement | 1.37E-03 |  | 3 |
| Cellular Movement | 1.40E-03 |  | 14 |
| Cellular Movement | 1.49E-03 |  | 9 |
| Cellular Movement | 1.61E-03 |  | 18 |
| Cellular Movement | 1.67E-03 |  | 4 |
| Cellular Movement | 1.79E-03 |  | 10 |
| Cellular Movement | 1.95E-03 |  | 6 |
| Cellular Movement | 2.03E-03 |  | 5 |
| Cellular Movement | 2.07E-03 |  | 12 |
| Cellular Movement | 2.11E-03 |  | 7 |
| Cellular Movement | 2.53E-03 |  | 7 |
| Cellular Movement | 2.61E-03 |  | 13 |
| Cellular Movement | 3.49E-03 |  | 18 |
| Cellular Movement | 3.54E-03 |  | 16 |
| Cellular Movement | 3.55E-03 |  | 2 |
| Cellular Movement | 3.55E-03 |  | 2 |
| Cellular Movement | 3.56E-03 |  | 7 |
| Connective Tissue Development and Function | 7.11E-05 |  | 8 |
| Connective Tissue Development and Function | 3.65E-04 |  | 14 |
| Connective Tissue Development and Function | 3.68E-04 |  | 3 |
| Connective Tissue Development and Function | 6.22E-04 |  | 4 |
| Connective Tissue Development and Function | 7.66E-04 |  | 3 |
| Connective Tissue Development and Function | 9.93E-04 |  | 18 |
| Connective Tissue Development and Function | 1.06E-03 |  | 4 |
| Connective Tissue Development and Function | 1.55E-03 |  | 6 |
| Connective Tissue Development and Function | 1.69E-03 |  | 11 |
| Connective Tissue Development and Function | 1.69E-03 |  | 21 |
| Connective Tissue Development and Function | 2.10E-03 |  | 6 |
| Connective Tissue Development and Function | 2.52E-03 |  | 9 |
| Connective Tissue Development and Function | 2.61E-03 |  | 10 |
| Connective Tissue Development and Function | 2.71E-03 |  | 3 |
| Connective Tissue Development and Function | 3.72E-03 |  | 9 |
| Connective Tissue Disorders | 1.42E-08 |  | 58 |
| Connective Tissue Disorders | 1.15E-06 |  | 15 |
| Connective Tissue Disorders | 2.50E-06 |  | 14 |
| Connective Tissue Disorders | 4.16E-06 |  | 12 |
| Connective Tissue Disorders | 5.99E-06 |  | 47 |
| Connective Tissue Disorders | 1.58E-04 |  | 11 |
| Connective Tissue Disorders | 2.70E-04 |  | 8 |
| Connective Tissue Disorders | 3.69E-04 |  | 2 |
| Connective Tissue Disorders | 3.69E-04 |  | 2 |
| Connective Tissue Disorders | 6.49E-04 |  | 8 |
| Connective Tissue Disorders | 1.04E-03 |  | 31 |
| Connective Tissue Disorders | 2.16E-03 |  | 2 |
| Connective Tissue Disorders | 2.16E-03 |  | 2 |
| Dermatological Diseases and Conditions | 9.67E-06 |  | 32 |
| Dermatological Diseases and Conditions | 4.49E-05 |  | 24 |
| Dermatological Diseases and Conditions | 2.70E-04 |  | 8 |
| Dermatological Diseases and Conditions | 3.94E-04 |  | 16 |
| Dermatological Diseases and Conditions | 7.05E-04 |  | 9 |
| Dermatological Diseases and Conditions | 1.09E-03 |  | 2 |
| Dermatological Diseases and Conditions | 2.14E-03 |  | 8 |
| Dermatological Diseases and Conditions | 2.16E-03 |  | 2 |
| Dermatological Diseases and Conditions | 2.20E-03 |  | 3 |
| Dermatological Diseases and Conditions | 2.23E-03 |  | 5 |
| Dermatological Diseases and Conditions | 3.19E-03 |  | 7 |
| Developmental Disorder | 1.63E-04 |  | 6 |
| Developmental Disorder | 3.69E-04 |  | 2 |
| Developmental Disorder | 1.09E-03 |  | 2 |
| Developmental Disorder | 1.09E-03 |  | 2 |
| Developmental Disorder | 1.37E-03 |  | 3 |
| Developmental Disorder | 1.67E-03 |  | 4 |
| Developmental Disorder | 2.16E-03 |  | 2 |
| Developmental Disorder | 3.55E-03 |  | 2 |
| Digestive System Development and Function | 3.37E-04 |  | 5 |
| Digestive System Development and Function | 1.75E-03 |  | 3 |
| Digestive System Development and Function | 1.76E-03 |  | 14 |
| Digestive System Development and Function | 2.16E-03 |  | 13 |
| Digestive System Development and Function | 2.16E-03 |  | 2 |
| Digestive System Development and Function | 3.14E-03 |  | 9 |
| Embryonic Development | 4.50E-06 |  | 4 |
| Embryonic Development | 7.11E-05 |  | 8 |
| Embryonic Development | 3.69E-04 |  | 2 |
| Embryonic Development | 3.69E-04 |  | 2 |
| Embryonic Development | 4.15E-04 |  | 4 |
| Embryonic Development | 6.22E-04 |  | 4 |
| Embryonic Development | 7.66E-04 |  | 3 |
| Embryonic Development | 9.93E-04 |  | 18 |
| Embryonic Development | 1.06E-03 |  | 4 |
| Embryonic Development | 1.37E-03 |  | 3 |
| Embryonic Development | 1.55E-03 |  | 6 |
| Embryonic Development | 1.69E-03 |  | 11 |
| Embryonic Development | 1.69E-03 |  | 21 |
| Embryonic Development | 1.84E-03 |  | 5 |
| Embryonic Development | 2.03E-03 |  | 5 |
| Embryonic Development | 2.16E-03 |  | 2 |
| Embryonic Development | 2.16E-03 |  | 2 |
| Embryonic Development | 2.52E-03 |  | 9 |
| Embryonic Development | 2.71E-03 |  | 3 |
| Embryonic Development | 2.81E-03 |  | 17 |
| Endocrine System Development and Function | 3.28E-03 |  | 3 |
| Endocrine System Development and Function | 3.28E-03 |  | 3 |
| Endocrine System Disorders | 3.69E-04 |  | 2 |
| Endocrine System Disorders | 4.22E-04 |  | 6 |
| Endocrine System Disorders | 1.09E-03 |  | 2 |
| Endocrine System Disorders | 3.55E-03 |  | 2 |
| Free Radical Scavenging | 9.61E-04 |  | 21 |
| Gastrointestinal Disease | 1.38E-04 |  | 8 |
| Gastrointestinal Disease | 2.38E-04 |  | 6 |
| Gastrointestinal Disease | 2.51E-04 |  | 14 |
| Gastrointestinal Disease | 3.37E-04 |  | 5 |
| Gastrointestinal Disease | 6.85E-04 |  | 18 |
| Gastrointestinal Disease | 7.63E-04 |  | 5 |
| Gastrointestinal Disease | 1.09E-03 |  | 7 |
| Gastrointestinal Disease | 1.69E-03 |  | 11 |
| Gastrointestinal Disease | 1.75E-03 |  | 3 |
| Gastrointestinal Disease | 1.76E-03 |  | 14 |
| Gastrointestinal Disease | 1.90E-03 |  | 11 |
| Gastrointestinal Disease | 2.16E-03 |  | 2 |
| Gastrointestinal Disease | 2.81E-03 |  | 4 |
| Gastrointestinal Disease | 3.55E-03 |  | 2 |
| Gene Expression | 3.55E-03 |  | 2 |
| Hair and Skin Development and Function | 2.16E-03 |  | 2 |
| Hematological Disease | 3.46E-08 |  | 15 |
| Hematological Disease | 2.70E-04 |  | 10 |
| Hematological Disease | 4.63E-04 |  | 7 |
| Hematological Disease | 6.73E-04 |  | 5 |
| Hematological Disease | 1.09E-03 |  | 2 |
| Hematological Disease | 1.22E-03 |  | 9 |
| Hematological Disease | 1.54E-03 |  | 7 |
| Hematological Disease | 2.16E-03 |  | 2 |
| Hematological Disease | 2.49E-03 |  | 4 |
| Hematological Disease | 4.04E-03 |  | 9 |
| Hematological System Development and Function | 1.20E-12 |  | 32 |
| Hematological System Development and Function | 8.40E-12 |  | 28 |
| Hematological System Development and Function | 1.08E-11 |  | 53 |
| Hematological System Development and Function | 2.83E-11 |  | 42 |
| Hematological System Development and Function | 1.37E-10 |  | 46 |
| Hematological System Development and Function | 1.93E-10 |  | 40 |
| Hematological System Development and Function | 3.18E-10 |  | 60 |
| Hematological System Development and Function | 3.95E-10 |  | 49 |
| Hematological System Development and Function | 6.11E-10 |  | 27 |
| Hematological System Development and Function | 1.00E-09 |  | 54 |
| Hematological System Development and Function | 1.43E-09 |  | 26 |
| Hematological System Development and Function | 3.18E-09 |  | 30 |
| Hematological System Development and Function | 3.64E-09 |  | 29 |
| Hematological System Development and Function | 3.71E-09 |  | 30 |
| Hematological System Development and Function | 8.08E-09 |  | 20 |
| Hematological System Development and Function | 1.63E-08 |  | 15 |
| Hematological System Development and Function | 3.35E-08 |  | 13 |
| Hematological System Development and Function | 4.24E-08 |  | 15 |
| Hematological System Development and Function | 5.46E-08 |  | 24 |
| Hematological System Development and Function | 1.00E-07 |  | 13 |
| Hematological System Development and Function | 1.00E-07 |  | 13 |
| Hematological System Development and Function | 1.11E-07 |  | 45 |
| Hematological System Development and Function | 2.96E-07 |  | 40 |
| Hematological System Development and Function | 3.91E-07 |  | 16 |
| Hematological System Development and Function | 3.94E-07 |  | 26 |
| Hematological System Development and Function | 4.91E-07 |  | 12 |
| Hematological System Development and Function | 5.31E-07 |  | 15 |
| Hematological System Development and Function | 5.67E-07 |  | 16 |
| Hematological System Development and Function | 2.88E-06 |  | 16 |
| Hematological System Development and Function | 3.19E-06 |  | 23 |
| Hematological System Development and Function | 3.87E-06 |  | 17 |
| Hematological System Development and Function | 4.33E-06 |  | 39 |
| Hematological System Development and Function | 4.43E-06 |  | 23 |
| Hematological System Development and Function | 4.50E-06 |  | 4 |
| Hematological System Development and Function | 5.09E-06 |  | 29 |
| Hematological System Development and Function | 5.92E-06 |  | 16 |
| Hematological System Development and Function | 7.42E-06 |  | 38 |
| Hematological System Development and Function | 7.43E-06 |  | 12 |
| Hematological System Development and Function | 8.16E-06 |  | 32 |
| Hematological System Development and Function | 1.17E-05 |  | 34 |
| Hematological System Development and Function | 1.28E-05 |  | 27 |
| Hematological System Development and Function | 1.50E-05 |  | 9 |
| Hematological System Development and Function | 1.73E-05 |  | 29 |
| Hematological System Development and Function | 1.94E-05 |  | 17 |
| Hematological System Development and Function | 2.39E-05 |  | 5 |
| Hematological System Development and Function | 2.40E-05 |  | 23 |
| Hematological System Development and Function | 3.60E-05 |  | 29 |
| Hematological System Development and Function | 3.89E-05 |  | 12 |
| Hematological System Development and Function | 4.03E-05 |  | 11 |
| Hematological System Development and Function | 4.10E-05 |  | 26 |
| Hematological System Development and Function | 4.12E-05 |  | 10 |
| Hematological System Development and Function | 4.41E-05 |  | 18 |
| Hematological System Development and Function | 4.77E-05 |  | 28 |
| Hematological System Development and Function | 5.20E-05 |  | 27 |
| Hematological System Development and Function | 7.58E-05 |  | 20 |
| Hematological System Development and Function | 1.03E-04 |  | 12 |
| Hematological System Development and Function | 1.07E-04 |  | 6 |
| Hematological System Development and Function | 1.15E-04 |  | 12 |
| Hematological System Development and Function | 1.16E-04 |  | 4 |
| Hematological System Development and Function | 1.16E-04 |  | 4 |
| Hematological System Development and Function | 1.21E-04 |  | 5 |
| Hematological System Development and Function | 1.23E-04 |  | 14 |
| Hematological System Development and Function | 1.43E-04 |  | 22 |
| Hematological System Development and Function | 1.45E-04 |  | 30 |
| Hematological System Development and Function | 1.49E-04 |  | 35 |
| Hematological System Development and Function | 1.61E-04 |  | 18 |
| Hematological System Development and Function | 1.65E-04 |  | 31 |
| Hematological System Development and Function | 1.87E-04 |  | 12 |
| Hematological System Development and Function | 1.88E-04 |  | 11 |
| Hematological System Development and Function | 1.94E-04 |  | 9 |
| Hematological System Development and Function | 1.99E-04 |  | 11 |
| Hematological System Development and Function | 2.26E-04 |  | 25 |
| Hematological System Development and Function | 2.30E-04 |  | 8 |
| Hematological System Development and Function | 2.38E-04 |  | 6 |
| Hematological System Development and Function | 2.40E-04 |  | 9 |
| Hematological System Development and Function | 2.54E-04 |  | 12 |
| Hematological System Development and Function | 2.59E-04 |  | 19 |
| Hematological System Development and Function | 2.68E-04 |  | 6 |
| Hematological System Development and Function | 2.70E-04 |  | 7 |
| Hematological System Development and Function | 2.75E-04 |  | 17 |
| Hematological System Development and Function | 2.82E-04 |  | 23 |
| Hematological System Development and Function | 3.08E-04 |  | 11 |
| Hematological System Development and Function | 3.68E-04 |  | 3 |
| Hematological System Development and Function | 3.69E-04 |  | 2 |
| Hematological System Development and Function | 3.69E-04 |  | 2 |
| Hematological System Development and Function | 4.08E-04 |  | 15 |
| Hematological System Development and Function | 4.08E-04 |  | 15 |
| Hematological System Development and Function | 4.14E-04 |  | 24 |
| Hematological System Development and Function | 4.15E-04 |  | 4 |
| Hematological System Development and Function | 4.37E-04 |  | 13 |
| Hematological System Development and Function | 4.69E-04 |  | 6 |
| Hematological System Development and Function | 4.87E-04 |  | 17 |
| Hematological System Development and Function | 5.11E-04 |  | 4 |
| Hematological System Development and Function | 5.18E-04 |  | 5 |
| Hematological System Development and Function | 5.24E-04 |  | 9 |
| Hematological System Development and Function | 5.44E-04 |  | 3 |
| Hematological System Development and Function | 5.68E-04 |  | 19 |
| Hematological System Development and Function | 5.92E-04 |  | 5 |
| Hematological System Development and Function | 5.94E-04 |  | 7 |
| Hematological System Development and Function | 6.22E-04 |  | 4 |
| Hematological System Development and Function | 6.28E-04 |  | 15 |
| Hematological System Development and Function | 7.01E-04 |  | 14 |
| Hematological System Development and Function | 7.02E-04 |  | 6 |
| Hematological System Development and Function | 7.53E-04 |  | 7 |
| Hematological System Development and Function | 7.91E-04 |  | 8 |
| Hematological System Development and Function | 9.85E-04 |  | 9 |
| Hematological System Development and Function | 1.01E-03 |  | 15 |
| Hematological System Development and Function | 1.04E-03 |  | 3 |
| Hematological System Development and Function | 1.06E-03 |  | 4 |
| Hematological System Development and Function | 1.06E-03 |  | 4 |
| Hematological System Development and Function | 1.09E-03 |  | 2 |
| Hematological System Development and Function | 1.09E-03 |  | 2 |
| Hematological System Development and Function | 1.17E-03 |  | 7 |
| Hematological System Development and Function | 1.19E-03 |  | 14 |
| Hematological System Development and Function | 1.24E-03 |  | 4 |
| Hematological System Development and Function | 1.28E-03 |  | 9 |
| Hematological System Development and Function | 1.29E-03 |  | 13 |
| Hematological System Development and Function | 1.37E-03 |  | 3 |
| Hematological System Development and Function | 1.40E-03 |  | 14 |
| Hematological System Development and Function | 1.49E-03 |  | 9 |
| Hematological System Development and Function | 1.57E-03 |  | 9 |
| Hematological System Development and Function | 1.61E-03 |  | 18 |
| Hematological System Development and Function | 1.67E-03 |  | 4 |
| Hematological System Development and Function | 1.67E-03 |  | 4 |
| Hematological System Development and Function | 1.75E-03 |  | 7 |
| Hematological System Development and Function | 1.75E-03 |  | 7 |
| Hematological System Development and Function | 1.78E-03 |  | 18 |
| Hematological System Development and Function | 1.79E-03 |  | 10 |
| Hematological System Development and Function | 2.03E-03 |  | 5 |
| Hematological System Development and Function | 2.03E-03 |  | 5 |
| Hematological System Development and Function | 2.07E-03 |  | 12 |
| Hematological System Development and Function | 2.12E-03 |  | 10 |
| Hematological System Development and Function | 2.19E-03 |  | 4 |
| Hematological System Development and Function | 2.20E-03 |  | 3 |
| Hematological System Development and Function | 2.44E-03 |  | 5 |
| Hematological System Development and Function | 2.44E-03 |  | 5 |
| Hematological System Development and Function | 2.53E-03 |  | 7 |
| Hematological System Development and Function | 2.53E-03 |  | 7 |
| Hematological System Development and Function | 2.61E-03 |  | 13 |
| Hematological System Development and Function | 2.71E-03 |  | 3 |
| Hematological System Development and Function | 3.28E-03 |  | 3 |
| Hematological System Development and Function | 3.30E-03 |  | 10 |
| Hematological System Development and Function | 3.49E-03 |  | 18 |
| Hematological System Development and Function | 3.54E-03 |  | 16 |
| Hematological System Development and Function | 3.55E-03 |  | 2 |
| Hematological System Development and Function | 3.55E-03 |  | 2 |
| Hematological System Development and Function | 3.55E-03 |  | 2 |
| Hematological System Development and Function | 3.55E-03 |  | 2 |
| Hematological System Development and Function | 3.56E-03 |  | 7 |
| Hematological System Development and Function | 3.67E-03 |  | 11 |
| Hematological System Development and Function | 3.70E-03 |  | 10 |
| Hematological System Development and Function | 3.93E-03 |  | 3 |
| Hematological System Development and Function | 3.93E-03 |  | 3 |
| Hematological System Development and Function | 3.96E-03 |  | 4 |
| Hematological System Development and Function | 3.97E-03 |  | 7 |
| Hematological System Development and Function | 4.02E-03 |  | 12 |
| Hematological System Development and Function | 4.29E-03 |  | 10 |
| Hematopoiesis | 4.50E-06 |  | 4 |
| Hematopoiesis | 8.16E-06 |  | 32 |
| Hematopoiesis | 1.17E-05 |  | 34 |
| Hematopoiesis | 1.73E-05 |  | 29 |
| Hematopoiesis | 3.60E-05 |  | 29 |
| Hematopoiesis | 1.65E-04 |  | 31 |
| Hematopoiesis | 2.26E-04 |  | 25 |
| Hematopoiesis | 2.82E-04 |  | 23 |
| Hematopoiesis | 3.69E-04 |  | 2 |
| Hematopoiesis | 3.69E-04 |  | 2 |
| Hematopoiesis | 4.08E-04 |  | 15 |
| Hematopoiesis | 4.14E-04 |  | 24 |
| Hematopoiesis | 4.15E-04 |  | 4 |
| Hematopoiesis | 5.18E-04 |  | 5 |
| Hematopoiesis | 5.68E-04 |  | 19 |
| Hematopoiesis | 7.01E-04 |  | 14 |
| Hematopoiesis | 8.53E-04 |  | 10 |
| Hematopoiesis | 9.85E-04 |  | 9 |
| Hematopoiesis | 1.06E-03 |  | 4 |
| Hematopoiesis | 1.75E-03 |  | 7 |
| Hematopoiesis | 1.95E-03 |  | 6 |
| Hematopoiesis | 2.16E-03 |  | 13 |
| Hematopoiesis | 2.19E-03 |  | 4 |
| Hematopoiesis | 2.71E-03 |  | 3 |
| Hematopoiesis | 3.93E-03 |  | 3 |
| Hematopoiesis | 3.96E-03 |  | 4 |
| Hematopoiesis | 4.29E-03 |  | 10 |
| Hepatic System Development and Function | 3.37E-04 |  | 5 |
| Hepatic System Development and Function | 1.75E-03 |  | 3 |
| Hepatic System Development and Function | 1.76E-03 |  | 14 |
| Hepatic System Disease | 2.38E-04 |  | 6 |
| Hepatic System Disease | 3.37E-04 |  | 5 |
| Hepatic System Disease | 1.75E-03 |  | 3 |
| Hepatic System Disease | 1.76E-03 |  | 14 |
| Hepatic System Disease | 1.90E-03 |  | 11 |
| Hepatic System Disease | 2.16E-03 |  | 2 |
| Hepatic System Disease | 2.81E-03 |  | 4 |
| Hereditary Disorder | 1.63E-04 |  | 6 |
| Hereditary Disorder | 1.09E-03 |  | 2 |
| Hereditary Disorder | 1.37E-03 |  | 3 |
| Hereditary Disorder | 1.67E-03 |  | 4 |
| Hereditary Disorder | 2.16E-03 |  | 2 |
| Hereditary Disorder | 2.71E-03 |  | 3 |
| Hereditary Disorder | 3.55E-03 |  | 2 |
| Humoral Immune Response | 1.10E-07 |  | 24 |
| Humoral Immune Response | 2.22E-06 |  | 21 |
| Humoral Immune Response | 5.80E-05 |  | 8 |
| Humoral Immune Response | 7.58E-05 |  | 20 |
| Humoral Immune Response | 2.38E-04 |  | 6 |
| Humoral Immune Response | 2.75E-04 |  | 17 |
| Humoral Immune Response | 2.97E-04 |  | 14 |
| Humoral Immune Response | 5.92E-04 |  | 5 |
| Humoral Immune Response | 7.91E-04 |  | 8 |
| Humoral Immune Response | 1.28E-03 |  | 9 |
| Humoral Immune Response | 1.67E-03 |  | 4 |
| Humoral Immune Response | 2.03E-03 |  | 5 |
| Humoral Immune Response | 3.55E-03 |  | 2 |
| Humoral Immune Response | 3.55E-03 |  | 2 |
| Humoral Immune Response | 3.66E-03 |  | 6 |
| Hypersensitivity Response | 7.66E-04 |  | 3 |
| Hypersensitivity Response | 1.04E-03 |  | 3 |
| Immune Cell Trafficking | 1.20E-12 |  | 32 |
| Immune Cell Trafficking | 8.40E-12 |  | 28 |
| Immune Cell Trafficking | 1.08E-11 |  | 53 |
| Immune Cell Trafficking | 2.83E-11 |  | 42 |
| Immune Cell Trafficking | 1.37E-10 |  | 46 |
| Immune Cell Trafficking | 1.93E-10 |  | 40 |
| Immune Cell Trafficking | 6.11E-10 |  | 27 |
| Immune Cell Trafficking | 6.46E-10 |  | 54 |
| Immune Cell Trafficking | 1.43E-09 |  | 26 |
| Immune Cell Trafficking | 3.18E-09 |  | 30 |
| Immune Cell Trafficking | 3.64E-09 |  | 29 |
| Immune Cell Trafficking | 3.71E-09 |  | 30 |
| Immune Cell Trafficking | 8.08E-09 |  | 20 |
| Immune Cell Trafficking | 1.63E-08 |  | 15 |
| Immune Cell Trafficking | 3.35E-08 |  | 13 |
| Immune Cell Trafficking | 5.46E-08 |  | 24 |
| Immune Cell Trafficking | 1.00E-07 |  | 13 |
| Immune Cell Trafficking | 3.91E-07 |  | 16 |
| Immune Cell Trafficking | 3.94E-07 |  | 26 |
| Immune Cell Trafficking | 5.31E-07 |  | 15 |
| Immune Cell Trafficking | 5.67E-07 |  | 16 |
| Immune Cell Trafficking | 2.88E-06 |  | 16 |
| Immune Cell Trafficking | 3.19E-06 |  | 23 |
| Immune Cell Trafficking | 3.87E-06 |  | 17 |
| Immune Cell Trafficking | 5.09E-06 |  | 29 |
| Immune Cell Trafficking | 1.28E-05 |  | 27 |
| Immune Cell Trafficking | 1.50E-05 |  | 9 |
| Immune Cell Trafficking | 1.94E-05 |  | 17 |
| Immune Cell Trafficking | 2.39E-05 |  | 5 |
| Immune Cell Trafficking | 2.40E-05 |  | 23 |
| Immune Cell Trafficking | 3.89E-05 |  | 12 |
| Immune Cell Trafficking | 4.03E-05 |  | 11 |
| Immune Cell Trafficking | 4.10E-05 |  | 26 |
| Immune Cell Trafficking | 4.12E-05 |  | 10 |
| Immune Cell Trafficking | 4.41E-05 |  | 18 |
| Immune Cell Trafficking | 1.03E-04 |  | 12 |
| Immune Cell Trafficking | 1.07E-04 |  | 6 |
| Immune Cell Trafficking | 1.15E-04 |  | 12 |
| Immune Cell Trafficking | 1.16E-04 |  | 4 |
| Immune Cell Trafficking | 1.16E-04 |  | 4 |
| Immune Cell Trafficking | 1.21E-04 |  | 5 |
| Immune Cell Trafficking | 1.23E-04 |  | 14 |
| Immune Cell Trafficking | 1.87E-04 |  | 12 |
| Immune Cell Trafficking | 1.94E-04 |  | 9 |
| Immune Cell Trafficking | 1.99E-04 |  | 11 |
| Immune Cell Trafficking | 2.38E-04 |  | 6 |
| Immune Cell Trafficking | 2.40E-04 |  | 9 |
| Immune Cell Trafficking | 2.54E-04 |  | 12 |
| Immune Cell Trafficking | 2.68E-04 |  | 6 |
| Immune Cell Trafficking | 2.70E-04 |  | 7 |
| Immune Cell Trafficking | 3.08E-04 |  | 11 |
| Immune Cell Trafficking | 3.68E-04 |  | 3 |
| Immune Cell Trafficking | 3.69E-04 |  | 2 |
| Immune Cell Trafficking | 4.37E-04 |  | 13 |
| Immune Cell Trafficking | 4.87E-04 |  | 17 |
| Immune Cell Trafficking | 5.11E-04 |  | 4 |
| Immune Cell Trafficking | 5.24E-04 |  | 9 |
| Immune Cell Trafficking | 5.92E-04 |  | 5 |
| Immune Cell Trafficking | 5.94E-04 |  | 7 |
| Immune Cell Trafficking | 7.02E-04 |  | 6 |
| Immune Cell Trafficking | 7.53E-04 |  | 7 |
| Immune Cell Trafficking | 7.91E-04 |  | 8 |
| Immune Cell Trafficking | 1.06E-03 |  | 4 |
| Immune Cell Trafficking | 1.17E-03 |  | 7 |
| Immune Cell Trafficking | 1.19E-03 |  | 14 |
| Immune Cell Trafficking | 1.28E-03 |  | 9 |
| Immune Cell Trafficking | 1.29E-03 |  | 13 |
| Immune Cell Trafficking | 1.37E-03 |  | 3 |
| Immune Cell Trafficking | 1.40E-03 |  | 14 |
| Immune Cell Trafficking | 1.49E-03 |  | 9 |
| Immune Cell Trafficking | 1.57E-03 |  | 9 |
| Immune Cell Trafficking | 1.61E-03 |  | 18 |
| Immune Cell Trafficking | 1.78E-03 |  | 18 |
| Immune Cell Trafficking | 1.79E-03 |  | 10 |
| Immune Cell Trafficking | 2.03E-03 |  | 5 |
| Immune Cell Trafficking | 2.07E-03 |  | 12 |
| Immune Cell Trafficking | 2.12E-03 |  | 10 |
| Immune Cell Trafficking | 2.44E-03 |  | 5 |
| Immune Cell Trafficking | 2.53E-03 |  | 7 |
| Immune Cell Trafficking | 2.61E-03 |  | 13 |
| Immune Cell Trafficking | 3.49E-03 |  | 18 |
| Immune Cell Trafficking | 3.54E-03 |  | 16 |
| Immune Cell Trafficking | 3.55E-03 |  | 2 |
| Immune Cell Trafficking | 3.56E-03 |  | 7 |
| Immunological Disease | 3.38E-07 |  | 49 |
| Immunological Disease | 1.15E-06 |  | 15 |
| Immunological Disease | 2.50E-06 |  | 14 |
| Immunological Disease | 4.16E-06 |  | 12 |
| Immunological Disease | 6.86E-05 |  | 3 |
| Immunological Disease | 2.04E-04 |  | 23 |
| Immunological Disease | 2.70E-04 |  | 8 |
| Immunological Disease | 2.70E-04 |  | 10 |
| Immunological Disease | 3.91E-04 |  | 5 |
| Immunological Disease | 3.94E-04 |  | 16 |
| Immunological Disease | 5.49E-04 |  | 21 |
| Immunological Disease | 1.04E-03 |  | 31 |
| Immunological Disease | 1.31E-03 |  | 17 |
| Immunological Disease | 2.16E-03 |  | 2 |
| Immunological Disease | 2.49E-03 |  | 4 |
| Immunological Disease | 3.19E-03 |  | 7 |
| Immunological Disease | 3.36E-03 |  | 8 |
| Infectious Disease | 2.96E-08 |  | 30 |
| Infectious Disease | 3.21E-05 |  | 21 |
| Infectious Disease | 4.08E-05 |  | 9 |
| Infectious Disease | 1.24E-04 |  | 11 |
| Infectious Disease | 1.35E-04 |  | 3 |
| Infectious Disease | 2.48E-04 |  | 11 |
| Infectious Disease | 2.70E-04 |  | 8 |
| Infectious Disease | 6.65E-04 |  | 9 |
| Infectious Disease | 6.73E-04 |  | 5 |
| Infectious Disease | 7.16E-04 |  | 11 |
| Infectious Disease | 1.06E-03 |  | 4 |
| Infectious Disease | 1.54E-03 |  | 7 |
| Infectious Disease | 2.23E-03 |  | 5 |
| Infectious Disease | 2.25E-03 |  | 8 |
| Infectious Disease | 2.76E-03 |  | 8 |
| Infectious Disease | 3.55E-03 |  | 2 |
| Inflammatory Disease | 1.42E-08 |  | 58 |
| Inflammatory Disease | 1.15E-06 |  | 15 |
| Inflammatory Disease | 2.50E-06 |  | 14 |
| Inflammatory Disease | 4.16E-06 |  | 12 |
| Inflammatory Disease | 5.99E-06 |  | 47 |
| Inflammatory Disease | 4.49E-05 |  | 24 |
| Inflammatory Disease | 1.38E-04 |  | 8 |
| Inflammatory Disease | 1.58E-04 |  | 11 |
| Inflammatory Disease | 2.51E-04 |  | 14 |
| Inflammatory Disease | 2.70E-04 |  | 8 |
| Inflammatory Disease | 3.37E-04 |  | 5 |
| Inflammatory Disease | 3.69E-04 |  | 2 |
| Inflammatory Disease | 3.94E-04 |  | 16 |
| Inflammatory Disease | 6.49E-04 |  | 8 |
| Inflammatory Disease | 6.85E-04 |  | 18 |
| Inflammatory Disease | 7.63E-04 |  | 5 |
| Inflammatory Disease | 1.04E-03 |  | 31 |
| Inflammatory Disease | 1.69E-03 |  | 11 |
| Inflammatory Disease | 1.75E-03 |  | 3 |
| Inflammatory Disease | 1.76E-03 |  | 14 |
| Inflammatory Disease | 3.19E-03 |  | 7 |
| Inflammatory Disease | 3.55E-03 |  | 2 |
| Inflammatory Response | 1.20E-12 |  | 32 |
| Inflammatory Response | 4.56E-12 |  | 51 |
| Inflammatory Response | 8.40E-12 |  | 28 |
| Inflammatory Response | 2.83E-11 |  | 42 |
| Inflammatory Response | 1.37E-10 |  | 46 |
| Inflammatory Response | 6.11E-10 |  | 27 |
| Inflammatory Response | 1.43E-09 |  | 26 |
| Inflammatory Response | 1.46E-09 |  | 22 |
| Inflammatory Response | 3.64E-09 |  | 29 |
| Inflammatory Response | 8.08E-09 |  | 20 |
| Inflammatory Response | 1.37E-08 |  | 16 |
| Inflammatory Response | 1.63E-08 |  | 15 |
| Inflammatory Response | 3.02E-08 |  | 19 |
| Inflammatory Response | 3.35E-08 |  | 13 |
| Inflammatory Response | 4.03E-08 |  | 26 |
| Inflammatory Response | 4.24E-08 |  | 15 |
| Inflammatory Response | 4.42E-08 |  | 16 |
| Inflammatory Response | 5.46E-08 |  | 24 |
| Inflammatory Response | 7.69E-08 |  | 15 |
| Inflammatory Response | 1.00E-07 |  | 13 |
| Inflammatory Response | 1.00E-07 |  | 13 |
| Inflammatory Response | 2.86E-07 |  | 14 |
| Inflammatory Response | 3.91E-07 |  | 16 |
| Inflammatory Response | 4.91E-07 |  | 12 |
| Inflammatory Response | 5.27E-07 |  | 16 |
| Inflammatory Response | 5.31E-07 |  | 15 |
| Inflammatory Response | 5.67E-07 |  | 16 |
| Inflammatory Response | 2.88E-06 |  | 16 |
| Inflammatory Response | 5.09E-06 |  | 29 |
| Inflammatory Response | 6.42E-06 |  | 19 |
| Inflammatory Response | 7.43E-06 |  | 12 |
| Inflammatory Response | 1.50E-05 |  | 9 |
| Inflammatory Response | 1.91E-05 |  | 7 |
| Inflammatory Response | 1.94E-05 |  | 17 |
| Inflammatory Response | 2.39E-05 |  | 5 |
| Inflammatory Response | 3.89E-05 |  | 12 |
| Inflammatory Response | 4.03E-05 |  | 11 |
| Inflammatory Response | 4.10E-05 |  | 26 |
| Inflammatory Response | 4.12E-05 |  | 10 |
| Inflammatory Response | 4.41E-05 |  | 18 |
| Inflammatory Response | 4.49E-05 |  | 24 |
| Inflammatory Response | 5.32E-05 |  | 15 |
| Inflammatory Response | 5.44E-05 |  | 50 |
| Inflammatory Response | 1.15E-04 |  | 12 |
| Inflammatory Response | 1.16E-04 |  | 4 |
| Inflammatory Response | 1.21E-04 |  | 5 |
| Inflammatory Response | 1.38E-04 |  | 8 |
| Inflammatory Response | 1.43E-04 |  | 22 |
| Inflammatory Response | 1.46E-04 |  | 5 |
| Inflammatory Response | 1.87E-04 |  | 12 |
| Inflammatory Response | 2.04E-04 |  | 4 |
| Inflammatory Response | 2.38E-04 |  | 6 |
| Inflammatory Response | 2.38E-04 |  | 6 |
| Inflammatory Response | 2.40E-04 |  | 9 |
| Inflammatory Response | 2.51E-04 |  | 14 |
| Inflammatory Response | 2.54E-04 |  | 12 |
| Inflammatory Response | 2.68E-04 |  | 6 |
| Inflammatory Response | 2.70E-04 |  | 7 |
| Inflammatory Response | 3.08E-04 |  | 11 |
| Inflammatory Response | 3.37E-04 |  | 5 |
| Inflammatory Response | 3.68E-04 |  | 3 |
| Inflammatory Response | 3.69E-04 |  | 2 |
| Inflammatory Response | 3.78E-04 |  | 6 |
| Inflammatory Response | 3.94E-04 |  | 16 |
| Inflammatory Response | 4.73E-04 |  | 15 |
| Inflammatory Response | 5.11E-04 |  | 4 |
| Inflammatory Response | 5.92E-04 |  | 5 |
| Inflammatory Response | 5.94E-04 |  | 7 |
| Inflammatory Response | 6.22E-04 |  | 4 |
| Inflammatory Response | 7.34E-04 |  | 10 |
| Inflammatory Response | 7.53E-04 |  | 7 |
| Inflammatory Response | 7.63E-04 |  | 5 |
| Inflammatory Response | 7.66E-04 |  | 3 |
| Inflammatory Response | 7.91E-04 |  | 8 |
| Inflammatory Response | 1.06E-03 |  | 4 |
| Inflammatory Response | 1.09E-03 |  | 2 |
| Inflammatory Response | 1.19E-03 |  | 14 |
| Inflammatory Response | 1.24E-03 |  | 4 |
| Inflammatory Response | 1.28E-03 |  | 9 |
| Inflammatory Response | 1.29E-03 |  | 13 |
| Inflammatory Response | 1.37E-03 |  | 3 |
| Inflammatory Response | 1.40E-03 |  | 14 |
| Inflammatory Response | 1.49E-03 |  | 9 |
| Inflammatory Response | 1.57E-03 |  | 9 |
| Inflammatory Response | 1.75E-03 |  | 3 |
| Inflammatory Response | 1.75E-03 |  | 7 |
| Inflammatory Response | 1.76E-03 |  | 14 |
| Inflammatory Response | 1.78E-03 |  | 18 |
| Inflammatory Response | 2.12E-03 |  | 10 |
| Inflammatory Response | 2.16E-03 |  | 2 |
| Inflammatory Response | 2.20E-03 |  | 3 |
| Inflammatory Response | 2.44E-03 |  | 5 |
| Inflammatory Response | 3.55E-03 |  | 2 |
| Inflammatory Response | 3.55E-03 |  | 2 |
| Inflammatory Response | 3.55E-03 |  | 2 |
| Inflammatory Response | 3.56E-03 |  | 7 |
| Inflammatory Response | 3.93E-03 |  | 3 |
| Inflammatory Response | 4.06E-03 |  | 5 |
| Lipid Metabolism | 3.61E-04 |  | 11 |
| Lipid Metabolism | 3.69E-04 |  | 2 |
| Lipid Metabolism | 6.76E-04 |  | 14 |
| Lipid Metabolism | 1.16E-03 |  | 13 |
| Lipid Metabolism | 1.37E-03 |  | 3 |
| Lipid Metabolism | 1.82E-03 |  | 8 |
| Lipid Metabolism | 1.87E-03 |  | 10 |
| Lipid Metabolism | 1.91E-03 |  | 4 |
| Lipid Metabolism | 2.39E-03 |  | 32 |
| Lipid Metabolism | 3.28E-03 |  | 3 |
| Lymphoid Tissue Structure and Development | 1.62E-06 |  | 29 |
| Lymphoid Tissue Structure and Development | 4.50E-06 |  | 4 |
| Lymphoid Tissue Structure and Development | 8.16E-06 |  | 32 |
| Lymphoid Tissue Structure and Development | 3.60E-05 |  | 29 |
| Lymphoid Tissue Structure and Development | 1.12E-04 |  | 15 |
| Lymphoid Tissue Structure and Development | 2.26E-04 |  | 25 |
| Lymphoid Tissue Structure and Development | 3.69E-04 |  | 2 |
| Lymphoid Tissue Structure and Development | 4.15E-04 |  | 4 |
| Lymphoid Tissue Structure and Development | 5.68E-04 |  | 19 |
| Lymphoid Tissue Structure and Development | 5.92E-04 |  | 5 |
| Lymphoid Tissue Structure and Development | 6.52E-04 |  | 20 |
| Lymphoid Tissue Structure and Development | 9.13E-04 |  | 18 |
| Lymphoid Tissue Structure and Development | 1.01E-03 |  | 15 |
| Lymphoid Tissue Structure and Development | 1.06E-03 |  | 4 |
| Lymphoid Tissue Structure and Development | 2.03E-03 |  | 5 |
| Lymphoid Tissue Structure and Development | 2.49E-03 |  | 4 |
| Lymphoid Tissue Structure and Development | 2.71E-03 |  | 3 |
| Lymphoid Tissue Structure and Development | 2.81E-03 |  | 17 |
| Lymphoid Tissue Structure and Development | 3.55E-03 |  | 2 |
| Lymphoid Tissue Structure and Development | 3.90E-03 |  | 6 |
| Lymphoid Tissue Structure and Development | 3.93E-03 |  | 3 |
| Lymphoid Tissue Structure and Development | 3.96E-03 |  | 4 |
| Lymphoid Tissue Structure and Development | 4.29E-03 |  | 10 |
| Metabolic Disease | 1.63E-04 |  | 6 |
| Metabolic Disease | 1.37E-03 |  | 3 |
| Metabolic Disease | 2.16E-03 |  | 2 |
| Metabolic Disease | 2.71E-03 |  | 3 |
| Metabolic Disease | 3.55E-03 |  | 2 |
| Molecular Transport | 1.64E-06 |  | 32 |
| Molecular Transport | 2.55E-06 |  | 30 |
| Molecular Transport | 8.19E-05 |  | 22 |
| Molecular Transport | 1.07E-04 |  | 21 |
| Molecular Transport | 1.18E-04 |  | 20 |
| Molecular Transport | 1.55E-04 |  | 4 |
| Molecular Transport | 3.16E-04 |  | 8 |
| Molecular Transport | 3.69E-04 |  | 2 |
| Molecular Transport | 3.73E-04 |  | 18 |
| Molecular Transport | 8.99E-04 |  | 8 |
| Molecular Transport | 1.02E-03 |  | 18 |
| Molecular Transport | 1.37E-03 |  | 3 |
| Molecular Transport | 1.50E-03 |  | 16 |
| Molecular Transport | 1.78E-03 |  | 23 |
| Molecular Transport | 1.87E-03 |  | 10 |
| Molecular Transport | 1.99E-03 |  | 19 |
| Molecular Transport | 2.81E-03 |  | 4 |
| Molecular Transport | 3.17E-03 |  | 4 |
| Molecular Transport | 3.28E-03 |  | 3 |
| Molecular Transport | 3.28E-03 |  | 3 |
| Molecular Transport | 3.55E-03 |  | 2 |
| Molecular Transport | 3.55E-03 |  | 2 |
| Molecular Transport | 3.55E-03 |  | 2 |
| Molecular Transport | 3.72E-03 |  | 9 |
| Nervous System Development and Function | 2.20E-03 |  | 3 |
| Neurological Disease | 1.09E-03 |  | 2 |
| Neurological Disease | 1.09E-03 |  | 2 |
| Neurological Disease | 1.67E-03 |  | 5 |
| Neurological Disease | 2.71E-03 |  | 3 |
| Neurological Disease | 3.55E-03 |  | 2 |
| Nutritional Disease | 2.16E-03 |  | 2 |
| Ophthalmic Disease | 1.09E-03 |  | 2 |
| Ophthalmic Disease | 3.96E-03 |  | 4 |
| Organ Development | 4.50E-06 |  | 4 |
| Organ Development | 7.11E-05 |  | 8 |
| Organ Development | 3.37E-04 |  | 5 |
| Organ Development | 3.69E-04 |  | 2 |
| Organ Development | 3.69E-04 |  | 2 |
| Organ Development | 4.15E-04 |  | 4 |
| Organ Development | 6.22E-04 |  | 4 |
| Organ Development | 7.66E-04 |  | 3 |
| Organ Development | 9.93E-04 |  | 18 |
| Organ Development | 1.06E-03 |  | 4 |
| Organ Development | 1.55E-03 |  | 6 |
| Organ Development | 1.69E-03 |  | 11 |
| Organ Development | 1.69E-03 |  | 21 |
| Organ Development | 1.75E-03 |  | 3 |
| Organ Development | 1.76E-03 |  | 14 |
| Organ Development | 1.84E-03 |  | 5 |
| Organ Development | 2.03E-03 |  | 5 |
| Organ Development | 2.16E-03 |  | 2 |
| Organ Development | 2.16E-03 |  | 2 |
| Organ Development | 2.52E-03 |  | 9 |
| Organ Development | 2.71E-03 |  | 3 |
| Organ Development | 2.81E-03 |  | 17 |
| Organ Morphology | 7.11E-05 |  | 8 |
| Organ Morphology | 3.69E-04 |  | 2 |
| Organ Morphology | 6.22E-04 |  | 4 |
| Organ Morphology | 6.52E-04 |  | 20 |
| Organ Morphology | 9.13E-04 |  | 18 |
| Organ Morphology | 9.93E-04 |  | 18 |
| Organ Morphology | 1.04E-03 |  | 3 |
| Organ Morphology | 1.55E-03 |  | 6 |
| Organ Morphology | 1.69E-03 |  | 21 |
| Organ Morphology | 2.16E-03 |  | 2 |
| Organ Morphology | 2.16E-03 |  | 2 |
| Organ Morphology | 2.49E-03 |  | 4 |
| Organ Morphology | 3.55E-03 |  | 2 |
| Organ Morphology | 3.93E-03 |  | 3 |
| Organismal Development | 4.50E-06 |  | 4 |
| Organismal Development | 7.11E-05 |  | 8 |
| Organismal Development | 2.10E-04 |  | 11 |
| Organismal Development | 3.69E-04 |  | 2 |
| Organismal Development | 3.69E-04 |  | 2 |
| Organismal Development | 3.95E-04 |  | 37 |
| Organismal Development | 4.09E-04 |  | 16 |
| Organismal Development | 4.15E-04 |  | 4 |
| Organismal Development | 5.08E-04 |  | 15 |
| Organismal Development | 5.62E-04 |  | 12 |
| Organismal Development | 5.77E-04 |  | 6 |
| Organismal Development | 6.22E-04 |  | 4 |
| Organismal Development | 7.66E-04 |  | 3 |
| Organismal Development | 9.93E-04 |  | 18 |
| Organismal Development | 1.06E-03 |  | 4 |
| Organismal Development | 1.55E-03 |  | 6 |
| Organismal Development | 1.69E-03 |  | 11 |
| Organismal Development | 1.69E-03 |  | 21 |
| Organismal Development | 1.84E-03 |  | 5 |
| Organismal Development | 2.03E-03 |  | 5 |
| Organismal Development | 2.16E-03 |  | 2 |
| Organismal Development | 2.16E-03 |  | 2 |
| Organismal Development | 2.52E-03 |  | 9 |
| Organismal Development | 2.71E-03 |  | 3 |
| Organismal Development | 2.81E-03 |  | 17 |
| Organismal Injury and Abnormalities | 6.86E-05 |  | 3 |
| Organismal Injury and Abnormalities | 1.24E-04 |  | 11 |
| Organismal Injury and Abnormalities | 1.68E-04 |  | 12 |
| Organismal Injury and Abnormalities | 4.81E-04 |  | 10 |
| Organismal Injury and Abnormalities | 1.09E-03 |  | 2 |
| Organismal Injury and Abnormalities | 1.09E-03 |  | 2 |
| Organismal Injury and Abnormalities | 1.43E-03 |  | 6 |
| Organismal Injury and Abnormalities | 1.54E-03 |  | 7 |
| Organismal Injury and Abnormalities | 1.90E-03 |  | 11 |
| Organismal Injury and Abnormalities | 2.16E-03 |  | 2 |
| Organismal Injury and Abnormalities | 2.50E-03 |  | 8 |
| Organismal Injury and Abnormalities | 2.81E-03 |  | 4 |
| Organismal Injury and Abnormalities | 3.55E-03 |  | 2 |
| Organismal Injury and Abnormalities | 3.89E-03 |  | 14 |
| Organismal Survival | 5.36E-04 |  | 10 |
| Organismal Survival | 6.07E-04 |  | 30 |
| Post-Translational Modification | 2.00E-03 |  | 12 |
| Protein Synthesis | 1.10E-07 |  | 24 |
| Protein Synthesis | 2.22E-06 |  | 21 |
| Protein Synthesis | 5.80E-05 |  | 8 |
| Protein Synthesis | 2.97E-04 |  | 14 |
| Protein Synthesis | 7.91E-04 |  | 8 |
| Protein Synthesis | 3.28E-03 |  | 3 |
| Protein Synthesis | 3.66E-03 |  | 6 |
| Psychological Disorders | 3.55E-03 |  | 2 |
| Renal and Urological Disease | 1.37E-03 |  | 3 |
| Renal and Urological Disease | 1.42E-03 |  | 9 |
| Renal and Urological Disease | 3.55E-03 |  | 2 |
| Renal and Urological System Development and Function | 3.55E-03 |  | 2 |
| Reproductive System Development and Function | 1.21E-03 |  | 6 |
| Reproductive System Disease | 3.13E-04 |  | 20 |
| Reproductive System Disease | 4.22E-04 |  | 6 |
| Reproductive System Disease | 1.09E-03 |  | 2 |
| Reproductive System Disease | 3.55E-03 |  | 2 |
| Respiratory Disease | 5.27E-06 |  | 126 |
| Respiratory Disease | 1.04E-05 |  | 122 |
| Respiratory Disease | 2.44E-05 |  | 114 |
| Respiratory Disease | 4.81E-04 |  | 10 |
| Respiratory Disease | 6.65E-04 |  | 9 |
| Respiratory Disease | 2.25E-03 |  | 8 |
| Respiratory Disease | 3.55E-03 |  | 2 |
| Respiratory System Development and Function | 8.93E-04 |  | 4 |
| Respiratory System Development and Function | 3.55E-03 |  | 2 |
| Skeletal and Muscular Disorders | 1.42E-08 |  | 58 |
| Skeletal and Muscular Disorders | 1.15E-06 |  | 15 |
| Skeletal and Muscular Disorders | 2.50E-06 |  | 14 |
| Skeletal and Muscular Disorders | 4.16E-06 |  | 12 |
| Skeletal and Muscular Disorders | 5.99E-06 |  | 47 |
| Skeletal and Muscular Disorders | 1.58E-04 |  | 11 |
| Skeletal and Muscular Disorders | 2.70E-04 |  | 8 |
| Skeletal and Muscular Disorders | 3.69E-04 |  | 2 |
| Skeletal and Muscular Disorders | 3.69E-04 |  | 2 |
| Skeletal and Muscular Disorders | 6.49E-04 |  | 8 |
| Skeletal and Muscular Disorders | 1.04E-03 |  | 31 |
| Skeletal and Muscular Disorders | 1.67E-03 |  | 4 |
| Skeletal and Muscular Disorders | 2.16E-03 |  | 2 |
| Skeletal and Muscular Disorders | 3.55E-03 |  | 2 |
| Skeletal and Muscular System Development and Function | 7.11E-05 |  | 8 |
| Skeletal and Muscular System Development and Function | 3.69E-04 |  | 2 |
| Skeletal and Muscular System Development and Function | 3.69E-04 |  | 2 |
| Skeletal and Muscular System Development and Function | 5.62E-04 |  | 12 |
| Skeletal and Muscular System Development and Function | 6.22E-04 |  | 4 |
| Skeletal and Muscular System Development and Function | 7.66E-04 |  | 3 |
| Skeletal and Muscular System Development and Function | 9.93E-04 |  | 18 |
| Skeletal and Muscular System Development and Function | 1.06E-03 |  | 4 |
| Skeletal and Muscular System Development and Function | 1.55E-03 |  | 6 |
| Skeletal and Muscular System Development and Function | 1.69E-03 |  | 11 |
| Skeletal and Muscular System Development and Function | 1.69E-03 |  | 21 |
| Skeletal and Muscular System Development and Function | 2.10E-03 |  | 6 |
| Skeletal and Muscular System Development and Function | 2.52E-03 |  | 9 |
| Skeletal and Muscular System Development and Function | 2.61E-03 |  | 10 |
| Skeletal and Muscular System Development and Function | 2.71E-03 |  | 3 |
| Small Molecule Biochemistry | 3.61E-04 |  | 11 |
| Small Molecule Biochemistry | 3.69E-04 |  | 2 |
| Small Molecule Biochemistry | 3.69E-04 |  | 2 |
| Small Molecule Biochemistry | 3.84E-04 |  | 10 |
| Small Molecule Biochemistry | 6.65E-04 |  | 9 |
| Small Molecule Biochemistry | 6.76E-04 |  | 14 |
| Small Molecule Biochemistry | 6.97E-04 |  | 7 |
| Small Molecule Biochemistry | 1.16E-03 |  | 13 |
| Small Molecule Biochemistry | 1.21E-03 |  | 6 |
| Small Molecule Biochemistry | 1.37E-03 |  | 3 |
| Small Molecule Biochemistry | 1.82E-03 |  | 8 |
| Small Molecule Biochemistry | 1.87E-03 |  | 10 |
| Small Molecule Biochemistry | 1.91E-03 |  | 4 |
| Small Molecule Biochemistry | 2.20E-03 |  | 3 |
| Small Molecule Biochemistry | 2.21E-03 |  | 10 |
| Small Molecule Biochemistry | 2.39E-03 |  | 32 |
| Small Molecule Biochemistry | 2.81E-03 |  | 4 |
| Small Molecule Biochemistry | 3.17E-03 |  | 4 |
| Small Molecule Biochemistry | 3.28E-03 |  | 3 |
| Small Molecule Biochemistry | 3.28E-03 |  | 3 |
| Tissue Development | 6.64E-09 |  | 31 |
| Tissue Development | 3.94E-07 |  | 26 |
| Tissue Development | 4.50E-06 |  | 4 |
| Tissue Development | 3.89E-05 |  | 12 |
| Tissue Development | 7.11E-05 |  | 8 |
| Tissue Development | 1.94E-04 |  | 9 |
| Tissue Development | 2.38E-04 |  | 6 |
| Tissue Development | 2.70E-04 |  | 7 |
| Tissue Development | 3.69E-04 |  | 2 |
| Tissue Development | 3.69E-04 |  | 2 |
| Tissue Development | 3.69E-04 |  | 2 |
| Tissue Development | 4.15E-04 |  | 4 |
| Tissue Development | 5.94E-04 |  | 7 |
| Tissue Development | 6.22E-04 |  | 4 |
| Tissue Development | 6.28E-04 |  | 15 |
| Tissue Development | 7.66E-04 |  | 3 |
| Tissue Development | 9.93E-04 |  | 18 |
| Tissue Development | 1.06E-03 |  | 4 |
| Tissue Development | 1.06E-03 |  | 4 |
| Tissue Development | 1.19E-03 |  | 14 |
| Tissue Development | 1.55E-03 |  | 6 |
| Tissue Development | 1.57E-03 |  | 9 |
| Tissue Development | 1.67E-03 |  | 4 |
| Tissue Development | 1.69E-03 |  | 11 |
| Tissue Development | 1.69E-03 |  | 21 |
| Tissue Development | 1.84E-03 |  | 5 |
| Tissue Development | 2.03E-03 |  | 5 |
| Tissue Development | 2.12E-03 |  | 10 |
| Tissue Development | 2.16E-03 |  | 2 |
| Tissue Development | 2.16E-03 |  | 2 |
| Tissue Development | 2.20E-03 |  | 3 |
| Tissue Development | 2.52E-03 |  | 9 |
| Tissue Development | 2.71E-03 |  | 3 |
| Tissue Development | 4.11E-03 |  | 16 |
| Tissue Morphology | 3.18E-10 |  | 60 |
| Tissue Morphology | 1.00E-09 |  | 54 |
| Tissue Morphology | 1.85E-07 |  | 76 |
| Tissue Morphology | 2.96E-07 |  | 40 |
| Tissue Morphology | 4.77E-05 |  | 28 |
| Tissue Morphology | 7.58E-05 |  | 20 |
| Tissue Morphology | 1.43E-04 |  | 22 |
| Tissue Morphology | 1.61E-04 |  | 18 |
| Tissue Morphology | 2.10E-04 |  | 11 |
| Tissue Morphology | 2.59E-04 |  | 19 |
| Tissue Morphology | 2.82E-04 |  | 23 |
| Tissue Morphology | 3.65E-04 |  | 14 |
| Tissue Morphology | 3.68E-04 |  | 3 |
| Tissue Morphology | 3.69E-04 |  | 2 |
| Tissue Morphology | 4.08E-04 |  | 15 |
| Tissue Morphology | 4.09E-04 |  | 16 |
| Tissue Morphology | 5.08E-04 |  | 15 |
| Tissue Morphology | 5.77E-04 |  | 6 |
| Tissue Morphology | 8.93E-04 |  | 4 |
| Tissue Morphology | 1.01E-03 |  | 15 |
| Tissue Morphology | 1.55E-03 |  | 6 |
| Tissue Morphology | 2.10E-03 |  | 6 |
| Tissue Morphology | 3.72E-03 |  | 9 |
| Tissue Morphology | 3.93E-03 |  | 3 |
| Tumor Morphology | 6.22E-04 |  | 4 |
| Tumor Morphology | 1.09E-03 |  | 2 |
| Visual System Development and Function | 3.69E-04 |  | 2 |
| Visual System Development and Function | 1.84E-03 |  | 5 |
| Visual System Development and Function | 2.16E-03 |  | 2 |
| Vitamin and Mineral Metabolism | 2.55E-06 |  | 30 |
| Vitamin and Mineral Metabolism | 8.19E-05 |  | 22 |
| Vitamin and Mineral Metabolism | 3.73E-04 |  | 18 |
| Vitamin and Mineral Metabolism | 6.65E-04 |  | 9 |
