# Supplementary material for: DNA Methylation Signature of Childhood Chronic Physical Aggression in T Cells of Both Men and Women
Source: PLoS One. 2014 Jan 24;9(1):e86822. doi: 10.1371/journal.pone.0086822 (PMC3901708; doi:10.1371/journal.pone.0086822)
Supplement: Table S3 — Canonical pathways enriched with genes whose methylation is associated with women aggression from IPA analysis (n = 430 genes). All of the p values were calculated using a right tailed Fisher’s exact test and corrected for multiple comparison with the Benjamini-Hochberg method. Significance threshold were p = 0.05. (DOCX) [file pone.0086822.s005.docx]

**Supplementary Table S3. Canonical pathways enriched with genes whose methylation is associated with women aggression from IPA analysis (n=430 genes).** All of the p values were calculated using a right tailed Fisher's exact test and corrected for multiple comparison with the Benjamini-Hochberg method. Significance threshold were p = 0.05.

| Ingenuity Canonical Pathways | p-value | Ratio | Molecules |
| --- | --- | --- | --- |
| Role of Hypercytokinemia/hyperchemokinemia in the Pathogenesis of Influenza | 1.90546E-05 | 1.59E-01 | CCR1,IL18,IFNA8,IL1RN,IL1F10,CCL3,IFNA14 |
| Role of IL-17A in Psoriasis | 8.31764E-05 | 3.08E-01 | S100A7,S100A9,CCL20,S100A8 |
| CCR5 Signaling in Macrophages | 9.54993E-05 | 8.33E-02 | GNG4,CD3G,GNAS,FCER1G,CCL3,CD3D,FAS,FASLG |
| Granulocyte Adhesion and Diapedesis | 0.000151356 | 6.74E-02 | IL18,C5AR1,CCL23,IL1RN,IL1RL1,PF4,FPR2,MMP10,CCL20,IL1F10,CCL3,CCL1 |
| Agranulocyte Adhesion and Diapedesis | 0.000251189 | 6.35E-02 | IL18,C5AR1,CCL23,IL1RN,PF4,MMP10,CCL20,IL1F10,CXCR1,CCL3,CD34,CCL1 |
| Tec Kinase Signaling | 0.000263027 | 6.04E-02 | BTK,GNG4,TXK,GNAS,TYK2,FCER1G,BMX,RHOH,FGR,FAS,FASLG |
| Graft-versus-Host Disease Signaling | 0.000269153 | 1.2E-01 | IL18,IL1RN,FCER1G,IL1F10,FAS,FASLG |
| FXR/RXR Activation | 0.000524807 | 7.92E-02 | PPARG,IL18,IL1RN,RARA,ABCB11,IL1F10,SLCO1B1,MTTP |
| Gαi Signaling | 0.000707946 | 6.72E-02 | PRKACB,GNG4,P2RY14,GNAS,NPY1R,OPRM1,FPR2,P2RY12,AGTR1 |
| Hepatic Cholestasis | 0.002290868 | 5.14E-02 | PRKACB,IL18,GNAS,IL1RN,IL1RL1,RARA,ABCB11,IL1F10,SLCO1B1 |
| IL-10 Signaling | 0.002630268 | 7.69E-02 | CCR1,IL18,IL1RN,IL1RL1,TYK2,IL1F10 |
| Role of JAK2 in Hormone-like Cytokine Signaling | 0.003981072 | 1.11E-01 | GHR,TYK2,GH1,SIRPA |
| Complement System | 0.003981072 | 1.14E-01 | MBL2,C5AR1,CFH,C8A |
| Role of Cytokines in Mediating Communication between Immune Cells | 0.004073803 | 9.09E-02 | IL18,IFNA8,IL1RN,IL1F10,IFNA14 |
| Altered T Cell and B Cell Signaling in Rheumatoid Arthritis | 0.006309573 | 6.52E-02 | IL18,IL1RN,FCER1G,IL1F10,FAS,FASLG |
| Tumoricidal Function of Hepatic Natural Killer Cells | 0.010715193 | 1.25E-01 | LYVE1,FAS,FASLG |
| LXR/RXR Activation | 0.010964782 | 5.15E-02 | IL18,IL1RN,IL1RL1,ACACA,S100A8,IL1F10,FGA |
| cAMP-mediated signaling | 0.011748976 | 4.42E-02 | PDE8A,PRKACB,P2RY14,GNAS,NPY1R,OPRM1,FPR2,P2RY12,AKAP7,AGTR1 |
| Pathogenesis of Multiple Sclerosis | 0.012022644 | 2.22E-01 | CCR1,CCL3 |
| Systemic Lupus Erythematosus Signaling | 0.012302688 | 4E-02 | CD3G,IL18,IFNA8,IL1RN,FCER1G,IL1F10,C8A,CD3D,IFNA14,PIM2 |
| 1,25-dihydroxyvitamin D3 Biosynthesis | 0.015135612 | 1.82E-01 | NADPH,CYP27B1 |
| Cytotoxic T Lymphocyte-mediated Apoptosis of Target Cells | 0.019952623 | 5.81E-02 | CD3G,FCER1G,CD3D,FAS,FASLG |
| p38 MAPK Signaling | 0.02630268 | 5.08E-02 | IL18,IL1RN,IL1RL1,IL1F10,FAS,FASLG |
| LPS/IL-1 Mediated Inhibition of RXR Function | 0.028840315 | 3.73E-02 | IL18,IL1RN,CYP2A13,IL1RL1,RARA,ACSL5,ABCB11,IL1F10,GSTP1 |
| Communication between Innate and Adaptive Immune Cells | 0.030902954 | 4.59E-02 | IL18,IL1RN,FCER1G,IL1F10,CCL3 |
| PPAR Signaling | 0.035481339 | 4.76E-02 | PPARG,IL18,IL1RN,IL1RL1,IL1F10 |
| NF-κB Signaling | 0.041686938 | 4.02E-02 | PRKACB,IL18,GHR,IL1RN,FCER1G,IL1F10,GH1 |
| T Cell Receptor Signaling | 0.046773514 | 4.59E-02 | BTK,CD3G,TXK,BMX,CD3D |
| Acute Phase Response Signaling | 0.047863009 | 3.89E-02 | IL18,MBL2,APCS,IL1RN,IL1F10,FGA,NR3C1 |
