# Supplementary material for: DNA Methylation Signature of Childhood Chronic Physical Aggression in T Cells of Both Men and Women
Source: PLoS One. 2014 Jan 24;9(1):e86822. doi: 10.1371/journal.pone.0086822 (PMC3901708; doi:10.1371/journal.pone.0086822)
Supplement: Table S4 — Upstream regulators showing a significant overlap with genes whose methylation is associated with aggression from IPA analysis (n = 430 genes). Upstream regulators differentially methylated between chronic and normal aggression are shown in bold. Significance threshold were P = 0.05. (DOCX) [file pone.0086822.s006.docx]

**Supplementary Table S4. Upstream regulators showing a significant overlap with genes whose methylation is associated with aggression from IPA analysis (n=430 genes).** Upstream regulators differentially methylated between chronic and normal aggression are shown in bold. Significance threshold were P = 0.05.

| Upstream Regulator | differential women CPA-NPA | Molecule Type | p-value of overlap | Target molecules in dataset |
| --- | --- | --- | --- | --- |
| TGM2 |  | enzyme | 2.79E-07 | ACSS2,AMICA1,C5AR1,CCL3,CD93,DEFA1 (includes others),DEFA4,FCER1G,MGP,MS4A3,RARA,RUNX2,S100A8,SIGLEC12,SIRPA,SLPI,SP110,VSIG4 |
| Lipo-poly-saccharide |  | chemical drug | 1.24E-06 | ABCB11,AGTR1,ANKRD22,APCS,BTK,C5AR1,CARD18,CCL1,CCL20,CCL3,CCR1,CD37,CPB2,CXCR1,CYP27B1,CYP2A13,DMBT1,FAS,FASLG,FCER1G,FPR2,FYB,GHR,GPR84,GSTP1,HDGF,IGSF6,IL18,IL1RN,IL21R,KLF6,MARCO,MBL2,MMP10,MTTP,NOX1,NR3C1,OPRM1,PF4,PIM2,PLEK,PPARG,PRDM1,RARA,RNASE2,RUNX2,S100A8,S100A9,SIRPA,SLC12A1,SLPI,TNFAIP6,TNIP3,TREM1,TREML2,TRIM21,VNN3,VSIG4 |
| TNF |  | cytokine | 5.49E-06 | ABCB11,ACACA,AGTR1,APCS,ARRDC3,C5AR1,CARD18,CCL1,CCL20,CCL3,CCR1,CXCR1,CYP27B1,DMBT1,EHF,ERG,ESM1,FAS,FASLG,FCAR,FCER1G,FPR2,GHR,GPR84,GSTP1,IL18,IL1RL1,IL1RN,IL21R,KLF6,LYVE1,MBL2,MEOX2,MGP,MMP10,MTTP,NR3C1,OPRM1,PIM2,PPARG,PRDM1,RARA,RNASE2,RUNX2,S100A7,S100A8,S100A9,SLC12A1,SLPI,TGIF1,TM4SF1,TNFAIP6,TREM1,TYK2 |
| **FAS** | -0.522 | transmembrane receptor | 6.16E-06 | BTK,CAST,CCL20,CCL3,CCR1,CD93,FAS,FASLG,FCAR,LILRA1,LUM,MGP,NR3C1,PLCL2,PLEC,RARA,RUNX2,SFTPC,SLPI,TNFAIP6,TYK2 |
| IL1B |  | cytokine | 1.37E-05 | ABCB11,AGTR1,APCS,BMF,CCL1,CCL20,CCL3,CCR1,CPB2,EHF,FAS,FASLG,FGF7,FPR2,GHR,IL18,IL1RL1,IL1RN,MMP10,P2RX7,PCSK1,PPARG,RARA,RNASE7,RUNX2,S100A8,S100A9,SLC12A1,SLCO1B1,SNCA,STMN2,TNFAIP6,TREM1 |
| mir-223 |  | microRNA | 1.53E-05 | CD180,CD34,CD93,CLEC2D,CRHBP,CTSG,EYA1,FBXW7,IL1RN,S100A9 |
| GATA3 |  | transcription regulator | 2.19E-05 | BMF,CCR1,CD3D,IL1RL1,OPRM1,PPARG,S100A8,S100A9,SLPI,TAL1 |
| IL1A |  | cytokine | 4.55E-05 | CCL20,CCL3,CLEC2D,FAS,FGF7,IL18,IL1RN,MMP10,NR3C1,PDZK1IP1,PPARG,S100A7,S100A8,S100A9 |
| dexamethasone |  | chemical drug | 6.06E-05 | ACACA,ACSS2,ASPH,BMX,C5AR1,C8A,C9orf72,CCL1,CCL20,CCL3,CCR1,CD3D,CLEC2D,CPB2,CRHBP,CXCR1,CYP27B1,DMP1,FASLG,FGA,FGF7,FGFBP1,GH1,GHR,GSTP1,IL18,IL1RL1,IL1RN,LMO2,LYVE1,MARCO,MGP,NR3C1,P2RX7,PCSK1,PF4,PIM2,PLCL2,PLEC,PLEK,PMCH,PPARG,RARA,RUNX2,S100A8,S100A9,SLC12A1,SLPI,TNFAIP6,TSFM,TXK |
| HGF |  | growth factor | 6.06E-05 | CD93,CHIA,DOK4,DSG1,EHF,ESM1,FASLG,FGF7,FPR2,GBA,GH1,HDGF,IL5RA,LYVE1,MEOX2,NDC80,PIM2,PMCH,PMP22,RUNX2,S100A9,TNFAIP6,TRDN |
| FOS |  | transcription regulator | 6.46E-05 | AGTR1,CAST,CD244,ESM1,FASLG,FGA,GBA,GRIK2,GSTP1,IL1RL1,KLF6,LTBP1,MMP10,PRDM1,RARA,S100A8,S100A9,SEMA3E,SIRPA,SLPI,TAL1,TPD52,TREM1 |
| HNF1A |  | transcription regulator | 7.01E-05 | ADH6,APCS,C8A,CPB2,F11,FAS,FBXO8,FCAMR,FGA,HAL,MRPL15,MTTP,NDUFS2,PCSK1,RUNX2,SERPINA7,SLCO1B1,SLPI,TACR3,TM4SF4,VNN3 |
| IFNG |  | cytokine | 7.50E-05 | AGTR1,BMF,C5AR1,CCL1,CCL20,CCL23,CCL3,CCR1,CLEC2D,DEGS1,EHF,ESM1,FAS,FASLG,FCER1G,FPR2,IL18,IL1RL1,IL1RN,KLF6,NOX1,P2RY14,PCSK1,PF4,PI4KB,PIM2,PLEK,PPARG,PRDM1,RNASE7,RUNX2,S100A8,S100A9,SIRPA,SLC12A1,SLPI,SP110,TGIF1,TNFAIP6,TREM1,TRIM21,TXK |
| tretinoin |  | chemical - endogenous mammalian | 8.29E-05 | ACACA,ACSS2,AGTR1,AMICA1,C5AR1,CCL20,CCL3,CCR1,CD34,CD93,CHN2,DEFA1 (includes others),DEFA4,DHRS9,DSG1,ETV1,FCAR,FCER1G,FGR,GHR,GPR21,GRIK2,GSTP1,HDGF,HOXD12,KRT5,MGP,MS4A3,NR3C1,PLEK,PPARG,RARA,RPS19,RUNX2,S100A8,S100A9,SFTPC,SIGLEC12,SIRPA,SLPI,SP110,TAL1,TNFAIP6,TPD52,TYK2,VSIG4 |
| IL1 |  | group | 9.20E-05 | ACACA,CCL20,CCL23,CCL3,CD34,FAS,FASLG,FGF7,IL1RL1,IL1RN,IL5RA,MMP10,MTTP,NOX1,PPARG,RUNX2,TNFAIP6 |
| ROCK2 |  | kinase | 2.03E-04 | DSG1,FAS,PPARG,S100A7,S100A8 |
| proteasome inhibitor PSI |  | chemical - protease inhibitor | 2.40E-04 | CCL3,CYP27B1,FAS,FASLG,RUNX2 |
| IL17R |  | complex | 3.26E-04 | CCL20,S100A7,S100A8,S100A9 |
| prostaglandin E2 |  | chemical - endogenous mammalian | 3.34E-04 | CCL20,CCL3,CCR1,CLEC2D,FAS,FASLG,FMOD,IL1RN,PPARG,PRDM1,RUNX2,S100A8,SLC12A1,TNFAIP6,TREM1 |
| PHLDA1 |  | other | 3.65E-04 | FAS,FASLG |
| 2,5-hexanedione |  | chemical toxicant | 3.65E-04 | FAS,FASLG |
| TLR4 |  | transmembrane receptor | 4.34E-04 | CAST,CCL1,CCL3,CD200R1,CYP27B1,FASLG,IFNA14,IFNA8,IL18,MARCO,PLEK,PPARG,PRDM1,TREM1,TRIM21 |
| **CYP27B1** | -0.912 | enzyme | 4.40E-04 | CCL1,CCL20,CYP27B1,IL18,SLC34A1 |
| peptidoglycan |  | chemical - endogenous non-mammalian | 5.11E-04 | CCL20,CCL3,FAS,FPR2,IL18,IL1RN,S100A8,S100A9 |
| TLR3 |  | transmembrane receptor | 5.14E-04 | CAST,CCL1,CCL20,CCL3,FAS,IFNA14,IFNA8,IL1RN,MBL2,S100A8,SLPI,TREM1,TREML2 |
| interferon beta-1a |  | biologic drug | 5.70E-04 | CD3D,FCER1G,FYB,GSTP1,IL1RN,RPS19,S100A9,SNCA,TNFAIP6 |
| IL4 |  | cytokine | 5.88E-04 | C5AR1,CCL1,CCL20,CCL23,CCL3,CXCR1,DSG1,FAS,FASLG,FCER1G,FPR2,IL18,IL1RL1,IL1RN,IL5RA,MARCO,MMP10,NR3C1,OPRM1,PIM2,POU2AF1,PPARG,PRDM1,S100A8,S100A9,SIRPA,TXK |
| SB203580 |  | chemical - kinase inhibitor | 6.24E-04 | CCL20,CCL3,EHF,FAS,FASLG,FPR2,FYB,GSTP1,IL18,IL1RN,LMO2,MMP10,RUNX2,SLPI,TNFAIP6,TREM1 |
| IL13 |  | cytokine | 6.34E-04 | CCL20,CCL23,CCL3,CD37,CHN2,FAS,FPR2,IL18,IL1RN,JAKMIP2,LTBP1,MMP10,OPRM1,PPARG,S100A8,SLPI,SNCA |
| CEBPA |  | transcription regulator | 6.54E-04 | ACACA,ADH6,CCL20,CCR1,CD3G,CPB2,CYP2A13,FCAR,GSTP1,IL1RN,PPARG,RUNX2,S100A8,S100A9,SEMA3E,SFTPC,TNFAIP6 |
| Vegf |  | group | 7.10E-04 | CD93,CHIA,DOK4,EHF,ESM1,FAS,GH1,IL18,IL5RA,LYVE1,MEOX2,MMP10,NDC80,P2RY14,PIM2,PMCH,PMP22,RAMP3 |
| GATA1 |  | transcription regulator | 7.43E-04 | CCR1,FYB,GNAS,IL1RL1,LMO2,PBXIP1,PF4,PIM2,RNASE2,TAL1 |
| MMP7 |  | peptidase | 7.54E-04 | FAS,FASLG,MMP10 |
| CEBPB |  | transcription regulator | 7.61E-04 | APCS,CCL3,CPB2,CYP27B1,CYP2A13,DEGS1,FAS,FCAR,IL1RN,MBL2,MGP,MMP10,PPARG,RUNX2,SEMA3E,TM4SF1,TNFAIP6 |
| IL10 |  | cytokine | 8.86E-04 | APCS,CCL1,CCL20,CCL23,CCL3,CCR1,EHF,FAS,FASLG,FCER1G,IL18,IL1RN,PRDM1,S100A8,TNIP3,TREM1 |
| N-cor |  | group | 9.47E-04 | ACACA,CCR1,CYP27B1,GSTP1,STMN2 |
| **IL18** | -0.958 | cytokine | 1.00E-03 | CCL20,CCL3,CD226,CD244,DEFA1 (includes others),FAS,FASLG,IL18,TXK |
| trinitrobenzenesulfonic acid |  | chemical reagent | 1.06E-03 | CCR1,IL18,LUM,MMP10,PF4,S100A8,S100A9 |
| BRD1 |  | other | 1.08E-03 | LMO2,TAL1 |
| PLA2G6 |  | enzyme | 1.08E-03 | PPARG,RUNX2 |
| mianserin |  | chemical drug | 1.08E-03 | NR3C1,OPRM1 |
| pydrin |  | chemical toxicant | 1.08E-03 | FAS,FASLG |
| phorbol myristate acetate |  | chemical drug | 1.10E-03 | AGTR1,BTK,C5AR1,CCL20,CCL3,CRHBP,CYP27B1,CYP2A13,DMBT1,DSG1,FAM13A,FAS,FASLG,FCER1G,FGFBP1,GSTP1,IL18,LYVE1,NOX1,PF4,PMCH,POU2AF1,PPARG,RARA,RHOH,S100A8,S100A9,SLPI,TNFAIP6,TPM3,TSFM,VSIG4 |
| **TREM1** | -0.650 | transmembrane receptor | 1.12E-03 | ASPH,CCL20,CCL23,CCL3,HTRA4,IL1RL1,LY9,MMP10,PIM2,PPARG,TNIP3 |
| IL17RA |  | transmembrane receptor | 1.21E-03 | CCL20,CCR1,S100A8,S100A9 |
| fluoride |  | chemical - endogenous mammalian | 1.34E-03 | FAS,FASLG,RUNX2 |
| RGS2 |  | other | 1.34E-03 | IL18,MARCO,PPARG |
| mir-196 |  | microRNA | 1.34E-03 | ERG,KRT5,S100A9 |
| apicidin |  | chemical - endogenous non-mammalian | 1.34E-03 | CCR1,FAS,FASLG |
| IL12 (complex) |  | complex | 1.38E-03 | CCL1,CCL20,CCL3,CD226,CD244,FAS,FASLG,IL18,MARCO,RPA2,TXK |
| hemozoin |  | chemical - endogenous non-mammalian | 1.41E-03 | CCL3,CCR1,S100A8,S100A9 |
| RAC2 |  | enzyme | 1.47E-03 | CCL1,CD244,PMP22,RUNX2,SLPI |
| morphine |  | chemical drug | 1.67E-03 | FAS,FASLG,GHR,GNAS,IL18,NR3C1,OPRM1,P2RX7 |
| methotrexate |  | chemical drug | 1.72E-03 | ACAA2,ACSS2,C8A,CCL3,CFH,FAS,FGA,FGF7,FPR2,IL18,NR3C1,SFTPC |
| rituximab |  | biologic drug | 1.72E-03 | FAS,FASLG,PRDM1 |
| ginkgolide B |  | chemical drug | 1.72E-03 | FASLG,PAFAH1B3,PF4 |
| CSF2 |  | cytokine | 2.02E-03 | C5AR1,CCL3,CCR1,CD180,CFH,FAS,FPR2,IL1RL1,IL1RN,IL5RA,MARCO,NOX1,PIM2,PMP22,PPARG,RARA,RHOH,TREM1 |
| ciprofibrate |  | chemical drug | 2.03E-03 | ABCB11,APCS,FGA,GSTP1,HAL,MBL2,PPARG,SLC4A4,TM4SF4 |
| teniposide |  | chemical drug | 2.14E-03 | FAS,FASLG |
| STRA6 |  | other | 2.14E-03 | PPARG,RARA |
| chlorophyll a |  | chemical - endogenous non-mammalian | 2.14E-03 | FAS,PPARG |
| rubitecan |  | chemical drug | 2.14E-03 | FAS,FASLG |
| LIPE |  | enzyme | 2.18E-03 | ACSS2,FMOD,IL1RL1,MEOX2,PDE8A,PPARG,RARA,RUNX2 |
| Ige |  | complex | 2.39E-03 | CCL1,CCL3,FCER1G,PPARG,RNASE3 |
| RGS10 |  | other | 2.39E-03 | CCR1,FAS,IL18,IL5RA,PF4 |
| ascorbic acid |  | chemical - endogenous mammalian | 2.73E-03 | BMF,DMP1,FAS,MGP,PMP22,RUNX2 |
| fluvoxamine |  | chemical drug | 2.76E-03 | CCR1,CD37,CXCR1,DEFA1 (includes others) |
| beta-estradiol |  | chemical - endogenous mammalian | 2.86E-03 | ACAA2,AGTR1,ATP2B2,CAST,CCL20,CCR1,CLEC2D,DHRS9,DLG2,FAS,FASLG,FGF7,FGFBP1,FMOD,GHR,GSTP1,HDGF,IGSF6,IL18,KLF6,KRT5,LTBP1,LUM,MAK,MGP,MTTP,NPY1R,NR3C1,OPRM1,PCDH10,PDZK1IP1,PITPNM2,PMP22,PPARG,RAMP3,RARA,RUNX2,S100A7,SLPI,TACR3,TM4SF1,TNFAIP6,TPD52,TPH2,TREML2,ZBTB18 |
| IL6 |  | cytokine | 2.96E-03 | ABCB11,ANG,APCS,C5AR1,CCL20,CCR1,CPB2,FAS,FASLG,FGA,FPR2,IL1RL1,IL1RN,MMP10,MTTP,OPRM1,PPARG,S100A7,S100A9,SERPINA7,SP110,TAL1,TYK2 |
| Immunoglobulin |  | complex | 3.08E-03 | CCL1,CCL3,CHN2,CXCR1,FAS,FCER1G,HAL,LILRB1,S100A8,S100A9,SIRPA |
| TNFSF12 |  | cytokine | 3.13E-03 | CCL3,CCR1,MMP10,PF4,S100A8,S100A9 |
| KL |  | enzyme | 3.23E-03 | CAST,CYP27B1,SLC34A1 |
| ACLY |  | enzyme | 3.51E-03 | ACACA,ACSS2 |
| cimetidine |  | chemical drug | 3.51E-03 | FAS,FASLG |
| NFIA |  | transcription regulator | 3.87E-03 | CYP2A13,RUNX2,SFTPC |
| 2-mercaptoacetate |  | chemical - endogenous non-mammalian | 3.87E-03 | FASLG,PPARG,TREML2 |
| KLF2 |  | transcription regulator | 3.93E-03 | CCL3,CD93,ECM1,FGR,IL1RL1,PPARG,RNASE2,RUNX2 |
| CLEC11A |  | growth factor | 3.97E-03 | CCL1,CD244,PMP22,RUNX2,SLPI |
| NFATC1 |  | transcription regulator | 4.29E-03 | CD3G,FAS,FASLG,GPC6,PPARG |
| CEBPD |  | transcription regulator | 4.31E-03 | CCL20,CPB2,CYP2A13,NOX1,PPARG,TNFAIP6 |
| IL9 |  | cytokine | 4.32E-03 | CCL20,IL5RA,LTBP1,PIM2 |
| TLR2 |  | transmembrane receptor | 4.50E-03 | CAST,CCR1,CYP27B1,FASLG,FPR2,IL18,IL1RN,TREM1 |
| IL5 |  | cytokine | 4.50E-03 | ACAA2,CCL3,CCR1,FAS,IL1RL1,IL5RA,LUM,PDLIM1,PMP22,PRDM1,RHOH,UCK2 |
| NR1H4 |  | ligand-dependent nuclear receptor | 4.51E-03 | ABCB11,ACACA,MTTP,NR3C1,PPARG,RARA,SLCO1B1 |
| vitamin K2 |  | chemical drug | 4.58E-03 | AGTR1,DSE,MGP |
| zinc |  | chemical drug | 4.58E-03 | ASPG,FAS,MGP,SLC30A8,SLC39A2,TMPRSS11D |
| AMPK |  | complex | 4.58E-03 | ACACA,BMF,FASLG,IL1RN,PPARG,RUNX2 |
| ISL1 |  | transcription regulator | 4.58E-03 | DCC,DLG2,ETV1,EYA1,ISL2,PPP1R1C |
| TNFRSF8 |  | transmembrane receptor | 4.63E-03 | CCL1,CCL3,FAS,FASLG,PIM2 |
| JAG2 |  | growth factor | 4.79E-03 | CCL20,CCR1,CXCR1,IL1RN |
| ZBTB16 |  | transcription regulator | 4.79E-03 | CD34,HOXD12,PF4,RUNX2 |
| NR1H3 |  | ligand-dependent nuclear receptor | 4.87E-03 | ACACA,APCS,IL1RN,PPARG,RARA,SLCO1B1 |
| MKL2 |  | transcription regulator | 5.17E-03 | CTSG,MS4A3,S100A8,S100A9,SLPI,TAL1 |
| Nfat (family) |  | group | 5.17E-03 | CCL3,DHRS9,ESM1,FAS,FASLG,KLF6 |
| **PMP22** | -0.577 | other | 5.20E-03 | P2RX7,PMP22 |
| Gnas |  | other | 5.20E-03 | CYP27B1,SLC34A1 |
| WNT10B |  | other | 5.20E-03 | PPARG,RUNX2 |
| CCR5 |  | G-protein coupled receptor | 5.29E-03 | CCL3,FAS,FASLG,SLPI |
| NFkB (complex) |  | complex | 5.33E-03 | CCL1,CCL20,CCL3,DMBT1,EHF,FAS,FASLG,FPR2,HDGF,IFNA14,IFNA8,IL18,IL1RN,MBL2,OPRM1,PCSK1,PIM2,PPARG,SLPI,TNFAIP6 |
| Salmonella enterica serotype abortus equi lipopolysaccharide |  | chemical toxicant | 5.56E-03 | CCL20,CCL23,CCL3,IL18,MMP10,PIM2,RHOH,TNIP3,TPD52 |
| NR0B2 |  | ligand-dependent nuclear receptor | 5.76E-03 | ABCB11,ACACA,MTTP,PPARG,SLCO1B1 |
| glucocorticoid |  | chemical drug | 5.88E-03 | CYP27B1,FAS,FASLG,GH1,GRIK2,KRT5,NR3C1,PPARG,SERPINA7,TPH2 |
| ACOX1 |  | enzyme | 6.08E-03 | APCS,GSDMC,GSTP1,HAL,KLF6,MBL2,SLC4A4,TM4SF4 |
| PLAU |  | peptidase | 6.18E-03 | ANG,C5AR1,S100A8,S100A9,SLPI |
| beta-glycerophosphoric acid |  | chemical - endogenous mammalian | 6.22E-03 | DMP1,MGP,RUNX2 |
| mir-21 |  | microRNA | 6.60E-03 | CCL1,CD180,CD226,FASLG,IL18,MARCO,SLPI,TRDN |
| NR1I2 |  | ligand-dependent nuclear receptor | 6.60E-03 | ANG,GSTP1,LTBP1,MGP,MTTP,PPARG,S100A8,SLCO1B1 |
| mifepristone |  | chemical drug | 6.77E-03 | ABCB11,CLEC3B,FAS,FASLG,NPY1R,NR3C1,OPRM1,P2RX7,RAMP3,RARA,RUNX2,TNFAIP6 |
| MEF2 |  | group | 7.15E-03 | IL1RN,MEOX2,PCDH10 |
| TSC22D3 |  | transcription regulator | 7.15E-03 | FASLG,PPARG,RUNX2 |
| RNASE1 |  | enzyme | 7.15E-03 | CCL1,CCL23,CCL3 |
| Rhox4b (includes others) |  | transcription regulator | 7.19E-03 | CD34,TAL1 |
| HLA-G |  | other | 7.19E-03 | FAS,LILRB1 |
| CASQ2 |  | other | 7.19E-03 | ASPH,TRDN |
| CAMP |  | other | 7.56E-03 | C5AR1,CCL20,CCL3,FPR2,IL18 |
| cycloheximide |  | chemical reagent | 7.83E-03 | AGTR1,BTK,CCR1,ERG,FAS,FASLG,IL1RL1,IL1RN,NR3C1,PPARG,PRDM1,RUNX2,S100A8,TNFAIP6 |
| E. coli lipopolysaccharide |  | chemical - endogenous non-mammalian | 8.07E-03 | CCL3,IL18,S100A8,S100A9,TREM1 |
| Collagen type I |  | complex | 8.07E-03 | CPB2,F11,HDGF,MMP10,RUNX2 |
| MST1 |  | growth factor | 8.17E-03 | FAS,IL18,IL1RN |
| **FBXW7** | 0.379 | transcription regulator | 8.17E-03 | FGFBP1,NR3C1,PPARG |
| **MTTP** | 0.627 | transporter | 8.17E-03 | MTTP,PPARG,RUNX2 |
| MED13 |  | transcription regulator | 8.17E-03 | ACACA,CYP2A13,PPARG |
| 9-cis-retinoic acid |  | chemical drug | 8.69E-03 | ABCB11,ACACA,CCL20,DHRS9,FASLG,MTTP,PPARG,RARA |
| CD3E |  | transmembrane receptor | 9.04E-03 | CD3D,CD3G,FAS,FASLG |
| PD 0325901 |  | chemical drug | 9.47E-03 | CYP27B1,SLC34A1 |
| HOXA2 |  | transcription regulator | 9.47E-03 | PHOX2B,RUNX2 |
| LPA |  | other | 9.47E-03 | GHR,NR3C1 |
| DLX5 |  | transcription regulator | 9.47E-03 | FGF7,RUNX2 |
| thymidine |  | chemical - endogenous mammalian | 9.47E-03 | FAS,MUS81 |
| TLR8 |  | transmembrane receptor | 9.80E-03 | CCL1,CYP27B1,IFNA14,IFNA8 |
| H89 |  | chemical - kinase inhibitor | 9.90E-03 | CCL3,CYP27B1,GSTP1,POU2AF1,S100A8,TNFAIP6,TREM1 |
| BCL6 |  | transcription regulator | 1.03E-02 | CCL3,FAS,FMOD,IL18,IL21R,MGP,PRDM1 |
| ELF1 |  | transcription regulator | 1.04E-02 | FCAR,FCER1G,PF4 |
| LY294002 |  | chemical - kinase inhibitor | 1.06E-02 | ACACA,ACSS2,ATP2B2,BTK,CCR1,DLG2,FAS,FGF7,FXYD2,GSTP1,IL1RN,MTTP,NDC80,PPARG,RAMP3,RUNX2,TREM1 |
| IL8 |  | cytokine | 1.09E-02 | CXCR1,FAS,FASLG,NOX1,NR3C1 |
| TLR9 |  | transmembrane receptor | 1.16E-02 | CAST,CCL3,FAS,IFNA14,IFNA8,IL18,PRDM1,S100A8 |
| **CCL3** | -0.796 | cytokine | 1.17E-02 | CCL20,CCL3,CCR1 |
| FCGR2A |  | transmembrane receptor | 1.17E-02 | CCL1,CCL3,FAS |
| RUNX1T1 |  | transcription regulator | 1.20E-02 | CCL3,CD34 |
| miR-135a-5p (and other miRNAs w/seed AUGGCUU) |  | mature microRNA | 1.20E-02 | PPARG,RUNX2 |
| CD70 |  | cytokine | 1.20E-02 | FAS,PRDM1 |
| DUSP14 |  | phosphatase | 1.20E-02 | IL18,MARCO |
| CSF1 |  | cytokine | 1.21E-02 | CCL3,FAS,FASLG,FCER1G,IL18,IL1RL1,MARCO,PPARG |
| forskolin |  | chemical toxicant | 1.25E-02 | AGTR1,CRHBP,CYP27B1,ESM1,FAS,FASLG,GH1,GNAS,GSTP1,NPY1R,OPRM1,PCSK1,PDC,PDLIM3,RUNX2,S100A8,SLC24A5,TM4SF1,TNFAIP6 |
| PTPRC |  | phosphatase | 1.30E-02 | CCR1,CXCR1,FASLG |
| ADAMTS12 |  | peptidase | 1.30E-02 | MGP,S100A8,S100A9 |
| EGR2 |  | transcription regulator | 1.31E-02 | CAST,DMBT1,FASLG,GHR,GOLGB1,PIP,PMP22 |
| MKL1 |  | transcription regulator | 1.37E-02 | CTSG,MS4A3,S100A8,S100A9,SLPI,TAL1 |
| C5 |  | cytokine | 1.37E-02 | APCS,C5AR1,CCL3,FASLG,MBL2,RNASE3 |
| SFTPA1 |  | transporter | 1.37E-02 | CCL3,ECM1,ESM1,FRMD6,IL1RL1 |
| GATA2 |  | transcription regulator | 1.37E-02 | CD34,IL1RL1,LMO2,PPARG,RNASE2 |
| INSIG1 |  | other | 1.43E-02 | ACACA,ACSS2,MARCO,PPARG,S100A8,S100A9 |
| STAT5a/b |  | group | 1.45E-02 | FCER1G,OPRM1,PIM2,PRDM1,RARA |
| HOXA7 |  | transcription regulator | 1.45E-02 | CD34,CD93,ERG |
| FGF23 |  | growth factor | 1.48E-02 | CYP27B1,SLC34A1 |
| HIST1H4A (includes others) |  | other | 1.48E-02 | GSTP1,PHOX2B |
| TNNI3 |  | transporter | 1.48E-02 | CCL1,CCR1 |
| CXCR7 |  | G-protein coupled receptor | 1.48E-02 | CFH,FMOD |
| SOCS2 |  | other | 1.48E-02 | GHR,PPARG |
| carmustine |  | chemical drug | 1.48E-02 | FAS,FASLG |
| bile salt |  | chemical - endogenous non-mammalian | 1.48E-02 | ABCB11,SLCO1B1 |
| CD40LG |  | cytokine | 1.59E-02 | CAST,CCL1,CCL20,CCL3,CCR1,FAS,FASLG,IL21R,MMP10,PLEK,PRDM1,RNASE3,TGIF1,TNFAIP6 |
| CASP1 |  | peptidase | 1.60E-02 | ACACA,IL18,PPARG |
| S100A6 |  | transporter | 1.60E-02 | CCL20,DHRS9,PCDHB2 |
| Sos |  | group | 1.61E-02 | CAST,ESM1,GBA,KLF6,MMP10,SEMA3E,SIRPA,SLPI,TPD52 |
| HOXA9 |  | transcription regulator | 1.63E-02 | CCL3,CD34,CD93,CPB2,CTSG,ERG,FPR2,S100A8 |
| cyclophosphamide |  | chemical drug | 1.69E-02 | CCL1,FAS,FGA,IL18,TGIF1 |
| ONECUT1 |  | transcription regulator | 1.73E-02 | ABCB11,AGTR1,APCS,CCT8,EHF,F11,FAS,LRRC59,PI4KB,STMN2,TM4SF4 |
| CD5 |  | transmembrane receptor | 1.74E-02 | CCL3,IL5RA,PIM2,RARA |
| APOB |  | transporter | 1.76E-02 | ACACA,PPARG,RUNX2 |
| sterol |  | chemical - endogenous mammalian | 1.76E-02 | ACACA,ACSS2,MTTP |
| FOXA1 |  | transcription regulator | 1.77E-02 | DSE,MGP,SFTPC,SLC12A1,SLPI,THBS3 |
| TCR |  | complex | 1.77E-02 | ARPP21,CCL1,CCL3,CCR1,FAS,FASLG,NR3C1,P2RX7,PRDM1,PRKACB,TXK |
| teriflunomide |  | chemical drug | 1.79E-02 | FAS,FASLG |
| miR-196a-5p (and other miRNAs w/seed AGGUAGU) |  | mature microRNA | 1.79E-02 | KRT5,S100A9 |
| MTA3 |  | other | 1.79E-02 | CCL3,PRDM1 |
| monensin |  | chemical - endogenous non-mammalian | 1.79E-02 | OPRM1,SLC4A4 |
| 1,10-phenanthroline |  | chemical toxicant | 1.79E-02 | CXCR1,FASLG |
| SCAP |  | other | 1.85E-02 | ACAA2,ACACA,ACSL5,ACSS2 |
| 15-deoxy-delta-12,14 -PGJ 2 |  | chemical - endogenous non-mammalian | 1.91E-02 | AGTR1,CCL3,FASLG,GSTP1,NOX1,PPARG,RUNX2,TGIF1 |
| thymine |  | chemical - endogenous mammalian | 1.91E-02 | FASLG |
| cortivazol |  | chemical reagent | 1.91E-02 | NR3C1 |
| 12 lipoxygenase |  | group | 1.91E-02 | NOX1 |
| Hrf1 |  | other | 1.91E-02 | MEOX2 |
| Ces1b/Ces1c |  | enzyme | 1.91E-02 | GHR |
| **FMOD** | -0.403 | other | 1.91E-02 | LUM |
| KLHL17 |  | other | 1.91E-02 | GRIK2 |
| FNDC3B |  | other | 1.91E-02 | RUNX2 |
| RDH11 |  | enzyme | 1.91E-02 | NR3C1 |
| MTX2 |  | transporter | 1.91E-02 | MTX1 |
| Myosin2 |  | complex | 1.91E-02 | PPARG |
| SRSF9 |  | enzyme | 1.91E-02 | NR3C1 |
| DNAJC14 |  | other | 1.91E-02 | AGTR1 |
| SFTPB |  | other | 1.91E-02 | SFTPC |
| CCNB1 |  | kinase | 1.91E-02 | FASLG |
| TRD |  | other | 1.91E-02 | FCER1G |
| sodium metavanadate |  | chemical reagent | 1.91E-02 | FASLG |
| mir-338 |  | microRNA | 1.91E-02 | RUNX2 |
| mir-217 |  | microRNA | 1.91E-02 | RUNX2 |
| mir-346 |  | microRNA | 1.91E-02 | BTK |
| KAT6A |  | enzyme | 1.91E-02 | GSTP1 |
| Scd2 |  | enzyme | 1.91E-02 | PPARG |
| ORM1 |  | other | 1.91E-02 | IL1RN |
| LINC00568 |  | other | 1.91E-02 | ECM1 |
| COL4A2 |  | other | 1.91E-02 | FAS |
| PSMA1 |  | peptidase | 1.91E-02 | GSTP1 |
| Zfp54 |  | other | 1.91E-02 | PPARG |
| 20-hydroxy-prostaglandin E2 |  | chemical - endogenous mammalian | 1.91E-02 | PPARG |
| Neb |  | other | 1.91E-02 | SLN |
| Zfp55 |  | other | 1.91E-02 | PPARG |
| Cyp2g1 |  | enzyme | 1.91E-02 | CYP2A13 |
| ZNF184 |  | other | 1.91E-02 | S100A9 |
| Creb5 |  | transcription regulator | 1.91E-02 | PPARG |
| PAGR1 |  | other | 1.91E-02 | NR3C1 |
| NOXA1 |  | other | 1.91E-02 | NOX1 |
| ICAM3 |  | transmembrane receptor | 1.91E-02 | PPARG |
| BTG1 |  | transcription regulator | 1.91E-02 | NR3C1 |
| RPN2 |  | enzyme | 1.91E-02 | GSTP1 |
| DUSP5 |  | phosphatase | 1.91E-02 | PRDM1 |
| CHRND |  | transmembrane receptor | 1.91E-02 | GH1 |
| SLC25A10 |  | transporter | 1.91E-02 | ACACA |
| 3-mercaptopicolinic acid |  | chemical - kinase inhibitor | 1.91E-02 | NR3C1 |
| diprenorphine |  | chemical drug | 1.91E-02 | OPRM1 |
| pomalidomide |  | chemical drug | 1.91E-02 | CCL3 |
| clorgyline |  | chemical drug | 1.91E-02 | GNAS |
| FR 49175 |  | chemical toxicant | 1.91E-02 | PAFAH1B3 |
| 1,4-phenylenebis(methylene)selenocyanate |  | chemical toxicant | 1.91E-02 | FASLG |
| lead nitrate |  | chemical toxicant | 1.91E-02 | GSTP1 |
| oxymorphone |  | chemical drug | 1.91E-02 | OPRM1 |
| beta-funaltrexamine |  | chemical reagent | 1.91E-02 | OPRM1 |
| nalorphine |  | chemical drug | 1.91E-02 | OPRM1 |
| carbon |  | chemical reagent | 1.91E-02 | GSTP1 |
| arachidic acid |  | chemical - endogenous non-mammalian | 1.91E-02 | SNCA |
| Dihydroxy-acetone |  | chemical - endogenous mammalian | 1.91E-02 | NR3C1 |
| POU4F1 |  | transcription regulator | 1.92E-02 | DCC,DLG2,ETV1,EYA1,ISL2,PPP1R1C |
| semaxinib |  | chemical drug | 1.92E-02 | DEFA1 (includes others),DEFA4,SLPI |
| OSM |  | cytokine | 1.96E-02 | CCL20,DEGS1,ECM1,FGA,IL18,LCE2C (includes others),MMP10,PDZK1IP1,PPARG,PRDM1,S100A7,S100A8,S100A9,SLPI,TAL1,TM4SF1,ZBTB18 |
| CD3 |  | complex | 1.99E-02 | ANG,CCL1,CCL20,CCL3,CCR1,CD226,DEFA1 (includes others),FAS,FASLG,FCER1G,FYB,GNAS,GOLGB1,IL21R,LTBP1,PAFAH1B3,PLEC,PLEK,RHOH,SLPI |
| diethylstilbestrol |  | chemical drug | 2.07E-02 | CYP2A13,FAS,FASLG,FGFBP1,FMOD,IGSF6,NR3C1,S100A8,SLPI |
| KDR |  | kinase | 2.10E-02 | BTK,NDC80,TAL1 |
| **FASLG** | 0.559 | cytokine | 2.10E-02 | FAS,FASLG,RUNX2 |
| E. coli B5 lipopoly-saccharide |  | chemical - endogenous non-mammalian | 2.10E-02 | CCL1,CCL20,CCR1,IL18,IL21R,IL5RA,PF4,PRDM1,TGIF1,TREM1 |
| camptothecin |  | chemical toxicant | 2.11E-02 | BTK,CAST,CCL20,CCL3,CCR1,CD93,FAS,FASLG,FCAR,IER5,LILRA1,NR3C1,PLCL2,PLEC,RARA,SFTPC,SLPI,TNFAIP6,TYK2 |
| glycerol |  | chemical - endogenous mammalian | 2.12E-02 | NR3C1,PPARG |
| PLA2G10 |  | enzyme | 2.12E-02 | FAS,FASLG |
| miR-9-5p (and other miRNAs w/seed CUUUGGU) |  | mature microRNA | 2.12E-02 | PMP22,PRDM1 |
| PCSK2 |  | peptidase | 2.12E-02 | PCSK1,PMCH |
| SMURF2 |  | enzyme | 2.12E-02 | RUNX2,TGIF1 |
| SR 144528 |  | chemical reagent | 2.12E-02 | CCR1,IL1RN |
| DL-fructose |  | chemical - endogenous mammalian | 2.12E-02 | ACACA,NR3C1 |
| IL3 |  | cytokine | 2.18E-02 | CD3D,CD3G,FAS,GPR21,HDGF,HOXD12,IL1RL1,IL5RA,PIM2,RPS19,TPD52,TYK2 |
| dihydrotestosterone |  | chemical - endogenous mammalian | 2.24E-02 | ADTRP,AGTR1,ETV1,FGF7,GHR,GSTP1,KRT5,LTBP1,MAK,NR3C1,PIP,PPARG,RUNX2,SFTPC,SLC4A4,SLCO1B1 |
| rifampin |  | chemical drug | 2.24E-02 | AGTR1,C9orf72,DSE,GSTP1,MGP,PLEC,SLCO1B1 |
| GAPDH |  | enzyme | 2.29E-02 | CCL20,CCL3,FCER1G |
| PML |  | transcription regulator | 2.29E-02 | FAS,MMP10,NR3C1 |
| PPARA |  | ligand-dependent nuclear receptor | 2.34E-02 | ACAA2,ACACA,ACSL5,ACSS2,ADTRP,APCS,C8A,CFH,CLEC2D,FGA,GSTP1,MBL2,MTTP,PPARG |
| CFTR |  | ion channel | 2.36E-02 | ACAA2,CFH,DMBT1,FAS,S100A8 |
| valproic acid |  | chemical drug | 2.37E-02 | ACACA,CCR1,CYP2A13,FAS,FASLG,FRMD6,IL1RL1,MTTP,NR3C1,PDZK1IP1,PPARG,RARA |
| PTH |  | other | 2.47E-02 | CYP27B1,DMP1,GOLGB1,MGP,RUNX2,SLC12A1,SLC34A1 |
| JINK1/2 |  | group | 2.48E-02 | FAS,FASLG |
| MMP3 |  | peptidase | 2.48E-02 | FGF7,MMP10 |
| isopentenyl diphosphate |  | chemical - endogenous mammalian | 2.48E-02 | CCL3,CCR1 |
| clenbuterol |  | chemical drug | 2.48E-02 | CAST,IL1RN |
| GTF2B |  | transcription regulator | 2.48E-02 | FASLG,GHR,PPARG |
| FOXP3 |  | transcription regulator | 2.49E-02 | FASLG,IL1RL1,PIM2,PRDM1 |
| CRH |  | cytokine | 2.49E-02 | FASLG,IL18,IL1RN,OPRM1 |
| ERBB2 |  | kinase | 2.52E-02 | ACAA2,ANG,CCL20,CD34,CLEC3B,EHF,ETV1,FAM134B,FGF7,GHR,LUM,NDC80,PDE8A,PHLDB1,PPARG,SIRPA,SLPI,TGIF1,TPD52 |
| TAZ |  | enzyme | 2.63E-02 | ESM1,KRT5,LUM,SFTPC |
| IL2 |  | cytokine | 2.64E-02 | AMICA1,CCL3,CD244,CD3D,CD3G,CXCR1,FAS,FASLG,FCER1G,FGF7,IL18,IL1RL1,KLF6,PIM2,PRDM1,UCK2 |
| 3M-011 |  | chemical reagent | 2.68E-02 | CCL3,IFNA14,IFNA8 |
| NOD2 |  | other | 2.68E-02 | CCL3,DMBT1,IL18 |
| CpG oligonucleotide |  | chemical drug | 2.70E-02 | CCR1,FAS,FASLG,GPR84,IL18,MARCO |
| MYD88 |  | other | 2.76E-02 | APCS,CD200R1,FPR2,IFNA14,IFNA8,IL18,MARCO,MMP10,S100A8 |
| RUNX1 |  | transcription regulator | 2.81E-02 | CD34,FAS,PIM2,RUNX2,S100A9 |
| Ahr-aryl hydrocarbon-Arnt |  | complex | 2.85E-02 | FAS,FASLG |
| TAF1 |  | transcription regulator | 2.85E-02 | GHR,RUNX2 |
| TFCP2 |  | transcription regulator | 2.85E-02 | CFH,FGA |
| PTPRE |  | phosphatase | 2.85E-02 | IL18,MARCO |
| EREG |  | growth factor | 2.85E-02 | IL18,TNFAIP6 |
| PLAGL1 |  | transcription regulator | 2.85E-02 | KRT20,PPARG |
| DLX3 |  | transcription regulator | 2.85E-02 | KRT28,S100A9 |
| spermine |  | chemical - endogenous mammalian | 2.85E-02 | CCL3,RUNX2 |
| nitric oxide |  | chemical - endogenous mammalian | 2.89E-02 | AGTR1,FAS,FASLG,FGF7,GSTP1,IL18,NR3C1 |
| rosiglitazone |  | chemical drug | 2.92E-02 | ACAA2,ACSS2,APOL3/APOL4,IL18,IL1RN,IL21R,KRT20,NOX1,PPARG,RUNX2,S100A8,S100A9,SLC12A1 |
| TAF4 |  | transcription regulator | 2.93E-02 | EHF,FMOD,IL1RN,LUM,OLFML3 |
| TGFB1 |  | growth factor | 3.04E-02 | ACAA2,B3GALT2,BMF,CCL20,CCL3,CCR1,CD34,CLEC2D,CYP27B1,ECM1,ESM1,FAS,FASLG,FCAR,FCER1G,FGF7,FPR2,GNAS,GPR21,HDGF,IL18,IL1RL1,IL1RN,LTBP1,LYVE1,MGP,MMP10,NDC80,P2RY14,PDZK1IP1,PPARG,RARA,RPA2,RUNX2,SFTPC,SIRPA,SLN,TGIF1,TNFAIP6,TPM3,UCK2 |
| KITLG |  | growth factor | 3.04E-02 | CD3D,CD3G,IL18,IL1RL1,IL1RN,MARCO,NR3C1,PLCL2,TAL1 |
| CTNNB1 |  | transcription regulator | 3.06E-02 | CD34,CYP2A13,ECM1,FAS,FCER1G,FGFBP1,GHR,HDGF,KRT5,LMO2,NOX1,PMCH,PMP22,PRDM1,RUNX2,S100A8,TBX5 |
| IL7 |  | cytokine | 3.10E-02 | CCL3,CD3D,CD3G,FAS,PIM2,RUNX2 |
| Wnt |  | group | 3.11E-02 | CD34,PPARG,RUNX2 |
| **RNASE2** | -0.529 | enzyme | 3.11E-02 | CCL1,CCL23,CCL3 |
| IL24 |  | cytokine | 3.11E-02 | EIF2A,FAS,FASLG |
| ELK1 |  | transcription regulator | 3.11E-02 | CCT8,GH1,RUNX2 |
| bromocriptine |  | chemical drug | 3.11E-02 | FAS,FASLG,PCSK1 |
| TMSB4 |  | group | 3.25E-02 | DMP1,RUNX2 |
| 6-ethylchenodeoxycholic acid |  | chemical drug | 3.25E-02 | NR3C1,PPARG |
| MT1E |  | other | 3.25E-02 | GSTP1,OPRM1 |
| CYP7A1 |  | enzyme | 3.25E-02 | ACACA,MTTP |
| concanamycin A |  | chemical drug | 3.25E-02 | FASLG,PPARG |
| helenalin |  | chemical - endogenous non-mammalian | 3.25E-02 | CCL20,PRDM1 |
| ESR2 |  | ligand-dependent nuclear receptor | 3.32E-02 | GSTP1,KRT20,LTBP1,PLEC,RARA,RUNX2 |
| memantine |  | chemical drug | 3.34E-02 | ATP2B2,GSTP1,SIRPA |
| tacrolimus |  | chemical drug | 3.36E-02 | CHN2,ETV1,FASLG,IL1RL1,NR3C1,PRKACB,RUNX2,SLC17A6 |
| indomethacin |  | chemical drug | 3.51E-02 | CD34,FGA,FGF7,GBA,GH1,IL1RL1,PPARG,S100A8,TGIF1 |
| SPI1 |  | transcription regulator | 3.56E-02 | CD180,CXCR1,FCER1G,IL18,IL1RN,OPRM1,TREM1 |
| 1-alpha, 25-dihydroxy vitamin D3 |  | chemical drug | 3.57E-02 | CCL20,CLEC2D,CYP27B1,FAS,GHR,IL18,IL1RN,LTBP1,NOX1,PPARG,RPA2,S100A8,S100A9,SLC34A1 |
| 3M-001 |  | chemical drug | 3.58E-02 | CCL3,IFNA14,IFNA8 |
| NKX2-5 |  | transcription regulator | 3.58E-02 | ACACA,KCNE1,TBX5 |
| SMO |  | G-protein coupled receptor | 3.58E-02 | FAS,KRT5,PRDM1,S100A8 |
| GC-GCR dimer |  | complex | 3.67E-02 | IL1RN,SLPI |
| TEK |  | kinase | 3.67E-02 | S100A8,S100A9 |
| ACACB |  | enzyme | 3.67E-02 | ACACA,PPARG |
| POU1F1 |  | transcription regulator | 3.67E-02 | GH1,GHR |
| mangiferin |  | chemical - endogenous non-mammalian | 3.67E-02 | ACACA,MTTP |
| TGAL copolymer |  | chemical drug | 3.67E-02 | IL1RN,P2RX7 |
| melatonin |  | chemical - endogenous mammalian | 3.72E-02 | ACACA,FAS,FASLG,NR3C1,RUNX2 |
| IL15 |  | cytokine | 3.72E-02 | CCL3,CCR1,CD226,CD244,DEFA1 (includes others),FAS,FASLG,FYB,GNAS,IL21R,PAFAH1B3,PLEK |
| PPP3CA |  | phosphatase | 3.76E-02 | FASLG,GSTP1,PMP22,RUNX2 |
| STAT5A |  | transcription regulator | 3.78E-02 | CYP27B1,FAS,FCER1G,PIP,PPARG,TPM3 |
| cholestane-3,7,26-triol |  | chemical - endogenous mammalian | 3.79E-02 | ABCB11 |
| 5beta-cholestane-3alpha,7alpha,12alpha,25-tetrol |  | chemical - endogenous mammalian | 3.79E-02 | ABCB11 |
| 3,7,12-trihydroxy-coprostanic acid |  | chemical - endogenous non-mammalian | 3.79E-02 | ABCB11 |
| trans-cinnamaldehyde |  | chemical toxicant | 3.79E-02 | FAS |
| 15-LOX |  | group | 3.79E-02 | PPARG |
| endrin |  | chemical toxicant | 3.79E-02 | PPARG |
| Rbp |  | group | 3.79E-02 | PPARG |
| EGOT |  | other | 3.79E-02 | RNASE2 |
| Hoxd |  | other | 3.79E-02 | HOXD12 |
| SLC27A4 |  | transporter | 3.79E-02 | PPARG |
| SAMM50 |  | other | 3.79E-02 | MTX1 |
| SOCS7 |  | other | 3.79E-02 | PPARG |
| RETSAT |  | enzyme | 3.79E-02 | PPARG |
| BCMO1 |  | enzyme | 3.79E-02 | PPARG |
| STAT3/5 |  | group | 3.79E-02 | PIM2 |
| Par |  | group | 3.79E-02 | CCL20 |
| ETV7 |  | transcription regulator | 3.79E-02 | RARA |
| **TRDN** | -0.452 | other | 3.79E-02 | ASPH |
| PTPRG |  | phosphatase | 3.79E-02 | CD34 |
| LGALS8 |  | other | 3.79E-02 | FASLG |
| miR-615-3p (miRNAs w/seed CCGAGCC) |  | mature microRNA | 3.79E-02 | PPARG |
| ncRNA-a3 |  | other | 3.79E-02 | TAL1 |
| NOXO1 |  | other | 3.79E-02 | NOX1 |
| OSTF1 |  | transcription regulator | 3.79E-02 | RUNX2 |
| MDFI |  | other | 3.79E-02 | FBXW7 |
| SHOX2 |  | transcription regulator | 3.79E-02 | RUNX2 |
| GCDH |  | enzyme | 3.79E-02 | ANG |
| PLK3 |  | kinase | 3.79E-02 | FAS |
| Hbb-ar |  | other | 3.79E-02 | GH1 |
| etorphine |  | chemical drug | 3.79E-02 | OPRM1 |
| thimerosal |  | chemical drug | 3.79E-02 | OPRM1 |
| OPC-31260 |  | chemical drug | 3.79E-02 | SLC12A1 |
| pralnacasan |  | chemical drug | 3.79E-02 | IL18 |
| levorphanol |  | chemical drug | 3.79E-02 | OPRM1 |
| cation |  | chemical - other | 3.79E-02 | GSTP1 |
| gamma tocopherol |  | chemical drug | 3.79E-02 | PPARG |
| emetine |  | chemical toxicant | 3.79E-02 | SLC17A6 |
| 3alpha,7alpha-dihydroxy-5beta-cholestanic acid |  | chemical - endogenous mammalian | 3.79E-02 | ABCB11 |
| aluminum |  | chemical drug | 3.79E-02 | RUNX2 |
| oleylamide |  | chemical - endogenous mammalian | 3.79E-02 | PPARG |
| L-2-oxo-4-thiazolidinecarboxylic acid |  | chemical drug | 3.79E-02 | IL18 |
| glucuronoxylomannan |  | chemical - endogenous non-mammalian | 3.79E-02 | FASLG |
| EPO |  | cytokine | 3.96E-02 | FAS,FASLG,GBA,IL18,IL1RL1,NR3C1,PBXIP1,PLCL2,TAL1,TPH2 |
| Mapk |  | group | 4.00E-02 | MMP10,NOX1,NR3C1,RUNX2,S100A8 |
| corticosteroid |  | chemical drug | 4.08E-02 | IL1RN,PPARG,SLPI |
| TET2 |  | other | 4.08E-02 | AMICA1,HAL,S100A8 |
| TNFAIP3 |  | enzyme | 4.08E-02 | CCL20,FAS,FASLG |
| STAT3 |  | transcription regulator | 4.08E-02 | C5AR1,CCL20,CCR1,FAS,FASLG,FCER1G,FGA,IL1RN,PCSK1,PIM2,PRDM1,S100A7,SP110 |
| LPIN1 |  | phosphatase | 4.11E-02 | ACACA,PPARG |
| BMPER |  | other | 4.11E-02 | MGP,RUNX2 |
| ATG7 |  | enzyme | 4.11E-02 | BMF,CYP2A13 |
| T |  | transcription regulator | 4.11E-02 | FMOD,MGP |
| naltrexone |  | chemical drug | 4.11E-02 | FASLG,OPRM1 |
| IRF5 |  | transcription regulator | 4.13E-02 | CCL3,IFNA14,PRDM1,SP110 |
| Tetrachloro-dibenzodioxin |  | chemical toxicant | 4.14E-02 | CCL1,CYP2A13,FAS,FASLG,MARCO,MBL2,PCSK1,PF4,PRDM1,S100A8,S100A9,SERPINA7 |
| VDR |  | transcription regulator | 4.15E-02 | ABCB11,CYP27B1,IL18,IL1RN,SLC34A1 |
| uric acid |  | chemical - endogenous mammalian | 4.34E-02 | CCL3,IL18,TREM1 |
| T3-TR-RXR |  | complex | 4.34E-02 | ACACA,FGA,GH1 |
| CD40 |  | transmembrane receptor | 4.41E-02 | CCL3,FAS,FASLG,IL18,IL1RN,IL5RA,POU2AF1,PRDM1 |
| CCL5 |  | cytokine | 4.51E-02 | C5AR1,CCL3,CCR1,PLEC |
| fluoxetine |  | chemical drug | 4.51E-02 | FGFBP1,GNAS,NR3C1,TPH2 |
| SREBF2 |  | transcription regulator | 4.51E-02 | ACAA2,ACACA,ACSS2,MTTP |
| PNPLA2 |  | enzyme | 4.56E-02 | ACAA2,MTTP |
| TLX3 |  | transcription regulator | 4.56E-02 | NPY1R,SLC17A6 |
| xanthohumol |  | chemical - endogenous non-mammalian | 4.56E-02 | RUNX2,SLN |
| CCL4 |  | cytokine | 4.56E-02 | CCL3,CCR1 |
| bilirubin |  | chemical - endogenous mammalian | 4.56E-02 | RUNX2,SLCO1B1 |
| naringenin |  | chemical - endogenous non-mammalian | 4.56E-02 | MTTP,NR3C1 |
| MMP2 |  | peptidase | 4.61E-02 | ANG,IL1RN,TYK2 |
| PD98059 |  | chemical - kinase inhibitor | 4.70E-02 | AGTR1,CCL20,CCL3,CCR1,CD37,DSG1,FAM13A,FASLG,FGFBP1,FPR2,IL1RN,MMP10,PLEC,PPARG,S100A8,SIRPA |
| TNFRSF1B |  | transmembrane receptor | 4.71E-02 | APCS,FAS,OPRM1,P2RX7 |
| APOE |  | transporter | 4.84E-02 | C5AR1,ECM1,IL1RN,LTBP1,MGP,PPARG,S100A8,S100A9 |
| LTA |  | cytokine | 4.89E-02 | APCS,CCL20,LYVE1 |
| Tlr |  | group | 4.95E-02 | CCL3,FPR2,IFNA14,IFNA8,TREM1 |
| REST |  | transcription regulator | 4.95E-02 | ARRDC3,OPRM1,PCSK1,STMN2,TPH2 |
| ERBB4 |  | kinase | 8.29E-02 | FGF7,GHR,PIM2,SLPI |
| STAT6 |  | transcription regulator | 1.05E-01 | CCL20,CCL23,FCER1G,IL1RN,OPRM1,PIM2,TXK |
| PKD1 |  | ion channel | 1.09E-01 | ASPH,IL1RL1,JAKMIP2,LTBP1,P2RX7,PI4KB |
| ADIPOQ |  | other | 2.06E-01 | ACACA,ACSL5,CCL20,PPARG |
| NFKBIA |  | transcription regulator | 2.80E-01 | CCL20,CCR1,ESM1,FAS,FASLG,IL1RN,MGP,PDLIM1,S100A8,S100A9 |
| simvastatin |  | chemical drug | 4.61E-01 | CCL20,CCL3,FGA,IL18 |
| gentamicin |  | chemical drug | 1.00E00 | APCS,GHR,KLF6,TTC36 |
