# Supplementary material for: DNA Methylation Signature of Childhood Chronic Physical Aggression in T Cells of Both Men and Women
Source: PLoS One. 2014 Jan 24;9(1):e86822. doi: 10.1371/journal.pone.0086822 (PMC3901708; doi:10.1371/journal.pone.0086822)
Supplement: Table S5 — Biological functions enriched with genes whose methylation is associated with aggression in both sexes from IPA analysis (women n = 430 genes and men n = 448 genes). (DOCX) [file pone.0086822.s007.docx]

**Supplementary Table S5. Biological functions enriched with genes whose methylation is associated with aggression in both sexes from IPA analysis (women n=430 genes and men n=448 genes).**

| Category | Analysis Name | p-value | Molecules |
| --- | --- | --- | --- |
| Cell-To-Cell Signaling and Interaction | women | 1.2E-12-3.55E-03 | LILRA1,APOL3/APOL4,IGSF6,CTSG,CCL20,SP110,EIF2A,GLIPR1L1,DSE,TXK,APCS,PDC,RARA,LUM,GPRC6A,PRDM1,LYVE1,OR4X2,TIMD4,FASLG,FGR,DMBT1,CD200R1,DEFA1 (includes others),GPR111,FMOD,FGFBP1,LILRB1,PCSK1,RAMP3,FGA,SNCA,ANG,CD34,ECM1,IL1RL1,IL21R,MEOX2,AKAP7,GPR21,S100A7,DTNA,MBL2,OR52H1,GRIK2,RNASE2,CCL1,GNG4,CCR1,NADPH,CLEC2D,C5AR1,TREML2,CCL23,P2RX7,GH1,LTBP1,S100A9,CD244,FCER1G,P2RY12,FCAR,IFNA8,POU2AF1,OR4B1,SLPI,OR2A2,OR8B4,OR52E2,CXCR1,IFNA14,NOX1,MTTP,F11,RUNX2,SLC12A1,ACSL5,CD226,CPB2,CAST,MARCO,TBX5,IL5RA,OR4C13,OR4S2,TNFAIP6,OPRM1,PF4,TYK2,CD93,BMX,FYB,CD3G,CRHBP,IL18,CD180,IL1RN,PLEK,AGTR1,SIRPA,TREM1,OR8B8,RASA4,OR51G1,GALNT1,CCL3,NR3C1,RHOH,FAS,RNASE3,S100A8,CFH,PCDH8,PI4KB,PPARG,GNAS,NPY1R,VANGL1,FPR2,OR4M1,CD3D,ERG,BTK,ESM1,DEFB116,FGF20,FCAMR |
| Cell-To-Cell Signaling and Interaction | men | 5.18E-04-2.6E-02 | FYN,PCDHGA12,PCDHB2,SYNE1,HTR1D,DICER1,PCDHGA3,AGTPBP1,SLC6A3,PCDHB8,LIFR,DTNA,DRD1,TLR1,AMICA1,PCDH8,IL17F,HIPK2,PPARG,ADORA3,PCDHGA11,MGST1,SELE,SCN9A,DCN,UTS2,MAPK8,PLCG1,AFP,GPR176,IL1R1,PDE4D,ALS2,PCDHGA5,ATP2B2,PLCZ1,IL33,GRM5,PLCB4,NRCAM,IL1RN,DPP6,SYT1,VDAC1,VGF,NEDD9,LRP1 |
| Hematological System Development and Function | women | 1.2E-12-4.29E-03 | LMO2,CTSG,CCL20,CD37,TXK,APCS,RARA,LUM,PRDM1,AMICA1,TIMD4,FASLG,FGR,DMBT1,CD200R1,PLEC,DEFA1 (includes others),PLCL2,LILRB1,PCSK1,FGA,CD34,ECM1,ANG,SNCA,GSTP1,SLC4A4,IL1RL1,IL21R,S100A7,MBL2,GBA,PBXIP1,GRIK2,FGF7,CCL1,RNASE2,CCR1,CLEC2D,C5AR1,TREML2,CCL23,GPR84,CDX4,RPS19,P2RX7,GH1,LTBP1,LY9,S100A9,BMF,CD244,FCER1G,S100A7A,P2RY12,FCAR,IFNA8,UBASH3A,POU2AF1,TAL1,SLPI,FBXW7,CXCR1,IFNA14,F11,RUNX2,CD226,CPB2,CAST,CYP27B1,MARCO,IL5RA,OPRM1,PF4,TYK2,CD93,FYB,CD3G,IL18,CD180,IL1RN,PLEK,SIRPA,SFTPC,TREM1,RASA4,GALNT1,CCL3,NR3C1,RHOH,FAS,S100A8,CFH,PPARG,GNAS,NPY1R,FPR2,VSIG4,CD3D,ERG,BTK,DEFB116,PIM2,FCAMR |
| Hematological System Development and Function | men | 1.06E-03-2.48E-02 | FYN,GAB2,MMP14,DICER1,IL20,TLR1,CLOCK,AMICA1,HIPK2,IL17F,NLRC4,PPARG,ADORA3,MGST1,DLL1,SELE,TIAM1,IRF4,UTS2,MAPK8,PLCG1,IL1R1,PDE4D,LTBP1,IL33,RCAN1,GNAI3,AQP9,IL1RN,VAV3,PIK3CB,NEDD9 |
| Immune Cell Trafficking | women | 1.2E-12-3.56E-03 | FCAR,IFNA8,POU2AF1,TAL1,CTSG,SLPI,CCL20,CXCR1,IFNA14,TXK,APCS,RARA,LUM,CD226,CPB2,PRDM1,AMICA1,CAST,TIMD4,FGR,FASLG,MARCO,DMBT1,IL5RA,OPRM1,CD200R1,PF4,TYK2,CD93,PLEC,DEFA1 (includes others),FYB,LILRB1,CD3G,IL18,CD180,IL1RN,FGA,SNCA,CD34,SIRPA,SFTPC,TREM1,IL1RL1,IL21R,GALNT1,CCL3,RHOH,NR3C1,FAS,S100A7,GBA,S100A8,CFH,CCL1,RNASE2,PPARG,CCR1,GNAS,C5AR1,TREML2,NPY1R,CCL23,FPR2,RPS19,P2RX7,GH1,CD3D,LTBP1,ERG,BTK,S100A9,CD244,DEFB116,S100A7A,FCER1G |
| Immune Cell Trafficking | men | 7.03E-03-2.48E-02 | PPARG,ADORA3,FYN,MGST1,SELE,TIAM1,MMP14,UTS2,MAPK8,IL1R1,PDE4D,IL33,GNAI3,AQP9,IL20,IL1RN,AMICA1,PIK3CB,IL17F,NEDD9 |
| Inflammatory Response | women | 1.2E-12-4.06E-03 | APOL3/APOL4,CTSG,CCL20,TXK,APCS,RARA,LUM,AMICA1,PRDM1,TIMD4,FGR,FASLG,DMBT1,CD200R1,PLEC,DEFA1 (includes others),TPM3,FMOD,LILRB1,PDE8A,PCSK1,FGA,CD34,SNCA,GSTP1,IL1RL1,IL21R,C8A,S100A7,MBL2,GBA,FGF7,CCL1,RNASE2,CCR1,C5AR1,TREML2,CCL23,RPS19,MMP10,KRT5,P2RX7,GH1,LTBP1,S100A9,CD244,S100A7A,FCER1G,P2RY12,FCAR,IFNA8,UBASH3A,POU2AF1,TAL1,SLPI,CXCR1,IFNA14,NOX1,MTTP,F11,ATP11C,CD226,ABCB11,CPB2,CAST,MUCL1,MARCO,IL5RA,TNFAIP6,OPRM1,PF4,CD93,TYK2,BMX,CD3G,IL18,CRHBP,CD180,IL1RN,PDLIM1,SLCO1B1,SIRPA,AGTR1,SFTPC,TREM1,RASA4,CCL3,NR3C1,RHOH,FAS,RNASE3,S100A8,CFH,PPARG,GNAS,NPY1R,FPR2,CD3D,ERG,BTK,DEFB116,FCAMR |
| Inflammatory Response | men | 1.06E-03-2.48E-02 | GAB2,FYN,MMP14,CIITA,IL31,DICER1,PRG4,IL20,TLR1,AMICA1,IL17F,NLRC4,PPARG,ADORA3,MGST1,SELE,TIAM1,SCN9A,DCN,UTS2,MAPK8,ACVR1,IL1R1,PDE4D,IL24,LTBP1,IL33,GNAI3,AQP9,IL17RB,IL1RN,VAV3,PIK3CB,ITGB6,NEDD9,AGTR1,PDX1 |
| Cellular Movement | women | 1.08E-11-3.56E-03 | FCAR,POU2AF1,CTSG,SLPI,FBXW7,CCL20,CXCR1,NOX1,DSE,TXK,SLC12A1,RUNX2,APCS,RARA,LUM,MGP,DMP1,CD226,CPB2,AMICA1,PRDM1,CAST,LYVE1,FGR,FASLG,DMBT1,TBX5,OPRM1,TM4SF4,PF4,TYK2,CD93,PLEC,TPM3,DEFA1 (includes others),BMX,mir-193,FMOD,FYB,FGFBP1,IL18,IL1RN,RAMP3,FGA,MAK,CD34,AGTR1,SIRPA,PHOX2B,SFTPC,TREM1,IL1RL1,IL21R,MEOX2,GALNT1,CCL3,FAS,HDGF,S100A7,ARRDC3,GBA,PCDH10,CHN2,DCC,S100A8,CFH,FGF7,CCL1,RNASE2,CCR1,PPARG,SEMA3E,C5AR1,GNAS,TREML2,CCL23,PMCH,FPR2,RPS19,MMP10,P2RX7,GH1,ERG,BTK,PMP22,S100A9,DEFB116,FCER1G,S100A7A,TM4SF1 |
| Cellular Movement | men | 2.34E-03-2.6E-02 | GAB2,ST6GAL1,POU3F2,CLDN7,DICER1,NEXN,DSE,LIFR,mir-154,FOXC2,CTNNA2,FKRP,MGP,ANGPT4,AMICA1,IL17F,HIPK2,ARHGAP24,ZBTB16,ADORA3,DLL1,TIAM1,SELE,DCN,SCN9A,UTS2,JUNB,mir-515,PTP4A1,IL24,PLD1,IL33,AQP9,IL17RB,NRCAM,IL1RN,ITGB6,AGTR1,HTN1,FYN,MMP14,CIITA,SLC16A4,AKAP11,mir-296,DRD1,CHDH,IL20,CTBP2,UNC5C,PPARG,ST8SIA1,ARHGEF12,ARTN,MAPK8,ACVR1,PLCG1,IL1R1,PDE4D,GNAI3,RCAN1,VAV3,ADD1,PIK3CB,NEDD9,LRP1 |
| Tissue Morphology | women | 3.18E-10-3.93E-03 | UBASH3A,POU2AF1,LMO2,TAL1,CLEC3B,CCL20,FBXW7,NOX1,MTTP,F11,TXK,RUNX2,APCS,RARA,LUM,MGP,CPB2,PRDM1,CYP27B1,MUS81,TIMD4,FASLG,IL5RA,OPRM1,PF4,CD200R1,TYK2,PLCL2,FMOD,FYB,ZBTB18,CD3G,IL18,PCSK1,CD180,IL1RN,PLEK,ISL2,CD34,SNCA,GSTP1,AGTR1,SIRPA,SLC4A4,SFTPC,IL1RL1,IL21R,GALNT1,CCL3,NR3C1,RHOH,FAS,HDGF,MBL2,GBA,DCC,S100A8,CFH,FGF7,ATN1,CCR1,PPARG,SLC34A1,C5AR1,CLEC2D,NPY1R,FPR2,RPS19,VSIG4,P2RX7,GH1,CD3D,ATP2B2,LTBP1,BTK,mir-124,GHR,S100A9,BMF,CD244,FCER1G,FGF20,EYA1,PIM2,PAFAH1B3 |
| Tissue Morphology | men | 3.32E-03-2.6E-02 | GAB2,SP6,MMP14,POU3F2,SYNE1,DICER1,TGIF1,SLC6A3,DSE,FOXC2,DTNA,PRG4,ALDH1A3,MGP,LTBP4,PPARG,TIAM1,ST8SIA1,ARTN,DCN,JUNB,IL1R1,TCF7L1,IL24,ALS2,ATP2B2,RCAN1,IL1RN,VAV3,CREM,ALDH1A2,ADD1,HIST1H1D,CLCN7,VGF,PPARGC1A |
| Cancer | women | 2.47E-09-3.72E-03 | CTSG,FRMD6,TMEM225,PCDHB8,DSE,KRT28,APCS,LUM,MGP,TIMD4,DACT3,TPM3,GPR111,MLIP,FMOD,FGFBP1,LILRB1,DSG1,NRXN1,LRRN3,OR52I1,ACSM1,PLAC1L,TBC1D5,CD34,ECM1,SCN11A,MAGEA12,IL1RL1,ZNF285,C8A,DTNA,ARRDC3,PCDH10,ASPH,GPC6,ATN1,RNASE2,CCR1,FERD3L,TREML2,OLFML3,RPS19,DUSP21,LY9,S100A7A,EYA1,PRKACB,DNAH1,NLRP14,KLF6,OR8B4,ASPG,ETV1,CAPS2,ATP11C,RUNX2,TIGD2,WDPCP,ABCB11,MUCL1,CYP27B1,CAST,MUS81,MARCO,TBX5,IL5RA,OR4C13,PF4,TYK2,BMX,TACR3,FYB,SLITRK6,CRHBP,NDC80,MAK,KCNE1,AGTR1,EHF,MRPL15,CCL3,RHOH,HDGF,RNASE3,PSKH2,PCDH8,CFH,KRT20,PITPNM2,PPARG,FCRLA,GNAS,NPY1R,mir-506,GOLGB1,FPR2,ERG,RPA2,BTK,PMP22,P2RY14,GHR,ARPP21,PIM2,LILRA1,C9orf72,LMO2,DLG2,SP110,MUC7,ZMAT1,SCD5,TXK,RARA,DMP1,GPRC6A,PRDM1,LYVE1,CHAMP1,FGR,FASLG,DMBT1,TM4SF4,RABL3,CD200R1,PLEC,DEFA1 (includes others),mir-193,SCN10A,TPD52,ANKRD26,PDE8A,ZBTB18,SRGAP3,SUCO,PCSK1,DHRS9,C1orf105,FGA,ANG,GSTP1,SLC4A4,ACTBL2,SLC30A8,MEOX2,PDZK1IP1,THBS3,EFCAB11,PIP,S100A7,MBL2,GBA,CHN2,TMEM71,DCC,CSN3,FGF7,SLC34A1,CLEC2D,MMP10,KRT5,P2RX7,GH1,LTBP1,ATP2B2,mir-124,S100A9,BMF,CD244,UBN1,FCRL3,TM4SF1,FCAR,TAL1,SLPI,CLEC3B,FBXW7,GPR148,CXCR1,SIGLEC12,MTTP,SLC12A1,ACSL5,CD226,C20orf26,FRMPD2,IGSF22,OR4S2,OPRM1,UBQLNL,GLYAT,SP140,CASC5,IL18,IL1RN,C2orf71,JAKMIP2,DCHS2,SLCO1B1,PDLIM3,PHOX2B,C4orf17,STMN2,TNIP3,SLC17A6,OR8B8,RASA4,IL1F10,NR3C1,TGM5,FAS,LMOD1,NEK7,S100A8,PI4KB,CA10,VANGL1,FAM134B,VSIG4,CD3D,C2orf88,DEFB116,ESM1,FAM13A |
| Cancer | men | 5.09E-08-2.74E-02 | SNTG1,WHSC1,SYTL2,PCBP1,GDAP2,TMEM225,NEXN,PCDHB8,BTBD11,DSE,ADAM7,MGP,HIPK2,SPATA18,SELE,RPL27,OR2AG2,PTPN3,PTP4A1,PLD1,PLAC1L,ZDHHC1,ITGB6,MMAA,SCN11A,SPINK7,DTNA,GLP2R,MFF,TTC23,SNRPD3,ASPH,UNC5C,OR13G1,MKRN3,EPB41L4A,EPHA3,SAA4,ZNF32,FAM107B,OR10A3,NRIP1,NUTF2,PPIP5K2,PPARGC1A,AKR1C3,RASA3,UIMC1,C11orf52,CLDN7,PCDHA5,OR8J1,mir-154,CTNNA2,CC2D2B,ABP1,IL17F,RECQL5,ZFYVE9,OR4K1,MGST1,TIAM1,POLE4,OR10H4,SAP30BP,EDC3,DCAF4L2,TTC14,ALDH1A2,BAZ2B,SYN3,CLDN14,SUV420H1,AGTR1,PDX1,OR10T2,SLC16A4,ANKS4B,PRG4,mir-296,LARP7,HPS4,PCDH8,PCDHA13,PITPNM2,PPARG,SLC25A53,RP1,NUP210L,ALS2,PCDHGA5,LDB2,TANGO6,OR4K2,VAV3,RXFP3,OTUD6A,GLTSCR1L,ISG20,GPR115,VWA8,CROCC,LCE4A,TMEM257,LIFR,NPSR1,ITIH4,ANGPT4,OR13C8,ZBTB16,LAYN,ADORA3,miR-190a-5p (and other miRNAs w/seed GAUAUGU),CAPN6,DFNA5,FCRL4,SCN9A,DCN,JUNB,mir-515,SLFN11,AQP9,NRCAM,MS4A4A,FAM198B,HIST1H3A (includes others),OR1J4,SYT1,FYN,NEB,ADAMTS14,TBL3,COX7A2L,OR5B3,REG4,SLC6A3,AKAP11,ALDH1A3,SORBS2,TPO,HRK,MAPK8,PLCG1,MYPN,ATP2B2,LTBP1,GRM5,mir-30,CALU,OR2T10,UBN1,TRIM48,MBNL1,NEDD9,LRP1,EHBP1,GAB2,TRIM51,SLC9A3,KRTAP9-4,SYNE1,DICER1,B3GALT1,IL1R2,FOXC2,C20orf26,KRT31,PCDHGA11,TNNI3K,DLL1,ZNF783,ICA1,PSMF1,MAP3K13,CLDN18,AFP,RGS6,STX2,IL24,IL33,NBPF15 (includes others),IL1RN,ASB15,DPP6,FPR3,PCDHGA12,MMP14,CIITA,POPDC2,NAT2,GNG11,CALD1,TMPRSS3,LTBP4,GADL1,ARHGEF12,IRF4,PCDHA12,MKNK2,TCF7L1,IL1R1,PDE4D,SLC5A8,PLCB4,ACOT2,PIK3CB,A1CF |
| Tissue Development | women | 6.64E-09-4.11E-03 | FCAR,PRKACB,POU2AF1,LMO2,TAL1,CTSG,SLPI,CLEC3B,CCL20,CXCR1,CD37,APCS,RUNX2,RARA,LUM,CD226,DMP1,MGP,GPRC6A,PRDM1,HOXD12,CAST,LYVE1,CYP27B1,FGR,FASLG,MARCO,DMBT1,TBX5,PF4,CD200R1,FYB,FMOD,CD3G,IL18,IL1RN,FGA,ISL2,CD34,ECM1,SIRPA,SLC4A4,IL1RL1,IL21R,GALNT1,MEOX2,CCL3,NR3C1,THBS3,FAS,S100A8,CCL1,PPARG,CCR1,SEMA3E,SLC34A1,C5AR1,CLEC2D,FPR2,TBX19,KRT5,P2RX7,GH1,CD3D,ERG,BTK,PMP22,EVX2,GHR,S100A9,FCER1G,P2RY12,EYA1,FCAMR,PIM2 |
| Tissue Development | men | 3.57E-04-2.23E-02 | SP6,SYNE1,POU3F2,DICER1,PCDHGA3,AGTPBP1,FOXC2,MYOM1,MGP,AMICA1,HIPK2,IL17F,SELE,PCDHGA11,DLL1,TIAM1,JUNB,PLD1,IL33,NRCAM,IL1RN,ALDH1A2,ITGB6,SUV420H1,ADAMTSL4,PDX1,FYN,NEB,PCDHGA12,MMP14,CIITA,AVPR1A,TGIF1,AKAP11,ALDH1A3,CALD1,PITPNM1,PCDH8,UNC5C,PPARG,ST8SIA1,IRF4,ARHGEF12,ARTN,MAPK8,RP1,TCF7L1,EPHA3,PCDHGA5,ATP2B2,LTBP1,PLCB4,ADD1,NEDD9,LRP1,PPARGC1A |
| Cellular Function and Maintenance | women | 1.37E-08-4.29E-03 | FCAR,IFNA8,POU2AF1,LMO2,TAL1,SLPI,CTSG,CCL20,IFNA14,TXK,APCS,RUNX2,SLC12A1,LUM,CD226,GPRC6A,PRDM1,CAST,TIMD4,FGR,FASLG,MARCO,IL5RA,OPRM1,CD200R1,PF4,CD93,TYK2,PLEC,DEFA1 (includes others),BMX,PLCL2,TPD52,FYB,LILRB1,CD3G,IL18,PCSK1,IL1RN,ANG,ECM1,KCNE1,SNCA,AGTR1,SIRPA,SLC4A4,TREM1,IL1RL1,IL21R,SLC30A8,RASA4,CCL3,NR3C1,RHOH,FAS,MBL2,S100A8,GRIK2,FXYD2,FGF7,CCL1,PPARG,CCR1,SLC34A1,CLEC2D,GNAS,C5AR1,TREML2,NPY1R,CCL23,GPR84,TRDN,PMCH,FPR2,VSIG4,MMP10,P2RX7,GH1,CD3D,ERG,ATP2B2,LTBP1,BTK,GHR,S100A9,BMF,CD244,FCER1G,P2RY12,FCAMR,PIM2 |
| Cellular Function and Maintenance | men | 3.57E-04-2.46E-02 | PPARG,FYN,ADAMTS14,SCN9A,DCN,MAPK8,PLCG1,ATF6,DICER1,IL24,FOXC2,mir-30,SYT1,VGF,VDAC1,HIPK2,NEDD9,LRP1,AGTR1,PPARGC1A |
| Connective Tissue Disorders | women | 1.42E-08-2.16E-03 | CTSG,CCL20,CXCR1,APCS,DEFA4,LUM,CD226,DMP1,PRDM1,CYP27B1,FASLG,TRIM21,IL5RA,TNFAIP6,OPRM1,C17orf59,CD200R1,TYK2,PLEC,DEFA1 (includes others),BMX,SCN10A,FMOD,IL18,CD180,IL1RN,TAGAP,FGA,CD34,SNCA,ANG,SIRPA,SCN11A,IL1RL1,CCL3,NR3C1,CHCHD2,FAS,RNASE3,MBL2,S100A8,RNASE2,CCL1,PPARG,CCR1,CA10,GNAS,C5AR1,CCL23,FPR2,VSIG4,MMP10,KRT5,P2RX7,GH1,CD3D,ERG,GHR,LY9,S100A9,CD244,FCER1G,FCRL3 |
| Connective Tissue Disorders | men | 3.57E-04-1.89E-02 | MMP14,CIITA,DICER1,TGIF1,IFT140,FOXC2,POLR1C,LARP7,LTBP4,HIPK2,ZBTB16,UNC5C,PPARG,SELE,MTRR,SCN9A,MAPK8,IL1R1,PDE4D,GRM5,GNAI3,PLCB4,IL1RN,EDARADD,CLCN7,VGF,DEAF1,SCN11A,AGTR1,PPARGC1A |
| Inflammatory Disease | women | 1.42E-08-3.55E-03 | SLPI,CTSG,CCL20,CXCR1,MTTP,TXK,ATP11C,APCS,DEFA4,RARA,LUM,CD226,ABCB11,PRDM1,MUCL1,FGR,FASLG,DMBT1,TRIM21,IL5RA,TNFAIP6,OPRM1,C17orf59,PF4,CD200R1,TYK2,TPM3,DEFA1 (includes others),BMX,SCN10A,FMOD,PDE8A,CD3G,IL18,CD180,IL1RN,TAGAP,RAMP3,FGA,SLCO1B1,CD34,SNCA,ANG,SIRPA,SCN11A,AGTR1,GSTP1,TREM1,IL1RL1,CCL3,NR3C1,FAS,CHCHD2,RNASE3,S100A7,MBL2,DCC,S100A8,CFH,RNASE2,CCL1,PPARG,CCR1,CA10,C5AR1,CCL23,FPR2,VSIG4,MMP10,KRT5,P2RX7,GH1,CD3D,ERG,GHR,LY9,S100A9,CD244,FCER1G,FCRL3 |
| Inflammatory Disease | men | 3.57E-04-2.04E-02 | PPARG,ADORA3,MMP14,ACVR1,CIITA,IL1R1,JUNB,PDE4D,IL33,PRG4,NPSR1,IL17RB,IL1RN,VAV3,LTBP4,ITGB6,AGTR1,PDX1 |
| Skeletal and Muscular Disorders | women | 1.42E-08-3.55E-03 | CTSG,CCL20,CXCR1,RUNX2,APCS,DEFA4,LUM,CD226,DMP1,HOXD12,PRDM1,CYP27B1,FASLG,TRIM21,IL5RA,TNFAIP6,C17orf59,OPRM1,CD200R1,TYK2,DEFA1 (includes others),BMX,SCN10A,FMOD,IL18,CD180,IL1RN,TAGAP,FGA,CD34,ANG,SNCA,GSTP1,SIRPA,SCN11A,IL1RL1,CCL3,NR3C1,CHCHD2,FAS,RNASE3,MBL2,S100A8,RNASE2,CCL1,PPARG,CCR1,CA10,GNAS,C5AR1,CCL23,FPR2,VSIG4,MMP10,P2RX7,GH1,CD3D,ERG,EVX2,GHR,LY9,S100A9,CD244,FCER1G,FCRL3 |
| Skeletal and Muscular Disorders | men | 3.57E-04-2.08E-02 | SP6,NEB,MMP14,CIITA,SYNE1,DICER1,TGIF1,SLC6A3,LIFR,IFT140,mir-154,POLR1C,FOXC2,PRG4,DRD1,FKRP,LARP7,MGP,ASPH,HIPK2,ZBTB16,MTRR,SCN9A,ACVR1,MAPK8,IL1R1,PDE4D,ALS2,RCAN1,GNAI3,PLCB4,IL1RN,DPP6,CLCN7,EDARADD,MBNL1,DEAF1,SCN11A,AGTR1,PPARGC1A |
| Infectious Disease | women | 2.96E-08-3.55E-03 | POU2AF1,TAL1,CTSG,SLPI,SP110,CXCR1,MTX1,TMPRSS11D,TXK,APCS,DEFA4,LUM,FASLG,FGR,MARCO,IL5RA,CD200R1,CD93,TYK2,DEFA1 (includes others),PDE8A,IL18,PCSK1,IL1RN,FGA,SNCA,SIRPA,TREM1,IL1RL1,IL21R,RASA4,CCL3,NR3C1,FAS,RNASE3,S100A7,MBL2,S100A8,FGF7,RNASE2,CCR1,PPARG,C5AR1,MMP10,VSIG4,P2RX7,BTK,HTATSF1,S100A9,ABO,FCER1G,P2RY12 |
| Infectious Disease | men | 7.03E-03-1.89E-02 | ST6GAL1,GAB2,SELE,IRF4,DCN,CIITA,MAPK8,PLCG1,IL1R1,IL33,AQP9,IL17RB,IL1RN,ZBTB14,TLR1,IL17F,NLRC4,PPARGC1A |
| Hematological Disease | women | 3.46E-08-4.04E-03 | TREM1,STMN2,IL1RL1,SLPI,CCL3,NR3C1,FAS,RNASE3,F11,MBL2,APCS,LUM,CPB2,GRIK2,FASLG,CCL1,RNASE2,PPARG,TNFAIP6,PF4,CD93,RPS19,GH1,ANKRD26,CD3G,IL18,GHR,ABO,S100A9,IL1RN,FCER1G,P2RY12,FGA,SIRPA,GSTP1 |
| Hematological Disease | men | 7.03E-03-1.89E-02 | SELE,MTRR,IL1RN,DOT1L,AFP,IL1R1,IL17F |
| Humoral Immune Response | women | 1.1E-07-3.66E-03 | IFNA8,IL1RL1,UBASH3A,POU2AF1,IL21R,TAL1,SLPI,C8A,GALNT1,NR3C1,CD37,IFNA14,FAS,F11,MBL2,APCS,RUNX2,CPB2,PRDM1,CFH,FGF7,TIMD4,FASLG,MARCO,CCR1,PPARG,C5AR1,IL5RA,NPY1R,TYK2,FPR2,DEFA1 (includes others),PLCL2,FMOD,BTK,IL18,CD180,IL1RN,BMF,CD244,FCER1G,ECM1,SIRPA,GSTP1,FCAMR,PIM2 |
| Humoral Immune Response | men | 1.01E-02-1.89E-02 | PPARG,FYN,DLL1,IRF4,VAV3,MMP14,PLCG1,DICER1,NEDD9 |
| Protein Synthesis | women | 1.1E-07-3.66E-03 | IL1RL1,IL21R,POU2AF1,GALNT1,NR3C1,CD37,FAS,APCS,CYP27B1,FGF7,TIMD4,FASLG,MARCO,PPARG,CCR1,SLC34A1,IL5RA,CLEC2D,NPY1R,FPR2,PLCL2,BTK,CD180,BMF,FCER1G,ECM1,SIRPA |
| Protein Synthesis | men | 3.44E-03-3.44E-03 | IL1RN,JUNB |
| Cellular Development | women | 1.11E-07-4.29E-03 | ERMN,LMO2,CCL20,CD37,TXK,APCS,RARA,DMP1,MGP,PRDM1,TIMD4,FASLG,DMBT1,PLCL2,TPD52,LILRB1,DSG1,ZBTB18,PCSK1,ACACA,ISL2,CD34,ANG,SNCA,GSTP1,IL1RL1,IL21R,PDZK1IP1,ARRDC3,PBXIP1,FGF7,ATN1,CCL1,RNASE2,CCR1,C5AR1,CLEC2D,CCL23,RPS19,TBX19,P2RX7,GH1,LTBP1,ATP2B2,mir-124,LY9,CD244,FCER1G,UBN1,P2RY12,EYA1,PRKACB,IFNA8,UBASH3A,POU2AF1,TAL1,KLF6,SLPI,FBXW7,IFNA14,RUNX2,CD226,CAST,CYP27B1,TBX5,IL5RA,PF4,TYK2,BMX,FYB,DPPA4,CD3G,IL18,CD180,IL1RN,KCNE1,SIRPA,PHOX2B,TREM1,STMN2,CCL3,NR3C1,RHOH,FAS,TGIF1,S100A8,KRT20,PPARG,GNAS,NPY1R,VANGL1,VSIG4,CD3D,ERG,BTK,PMP22,GHR,FGF20,ARPP21,FCAMR,PIM2 |
| Cellular Development | men | 3.15E-05-2.6E-02 | SP6,GAB2,AKR1C3,SLC9A3,ERMN,RASA3,POU3F2,SYNE1,DICER1,IL31,AGTPBP1,LIFR,TLR1,MGP,ZBTB16,ADORA3,DLL1,SELE,DCN,UTS2,JUNB,STX2,RGS6,IL24,IL33,IL1RN,ALDH1A2,AGTR1,PDX1,FYN,MMP14,CIITA,AVPR1A,TGIF1,SPINK7,PRG4,DRD1,IL20,CTBP2,HPS4,LTBP4,NLRC4,UNC5C,PPARG,SRA1,ST8SIA1,IRF4,DOT1L,ACVR1,MAPK8,PLCG1,DEDD,TCF7L1,IL1R1,PDE4D,ZNF32,ARID3A,LTBP1,ATP2B2,GRM5,RCAN1,INSM1,mir-30,VAV3,ADD1,UBN1,PIK3CB,MBNL1,VGF,LRP1,PPARGC1A |
| Cellular Growth and Proliferation | women | 1.11E-07-3.97E-03 | CTSG,CCL20,FRMD6,CD37,SCD5,TXK,RARA,LUM,MGP,DMP1,PRDM1,TIMD4,FASLG,TRIM21,TM4SF4,DACT3,PLEC,DEFA1 (includes others),TPM3,TPD52,PLCL2,mir-193,FMOD,FGFBP1,LILRB1,ACACA,SNCA,ANG,CD34,ECM1,GSTP1,IL1RL1,IL21R,NAA35,PDZK1IP1,S100A7,ARRDC3,MBL2,PBXIP1,CHN2,DCC,GRIK2,ASPH,FXYD2,FGF7,CCR1,GNG4,C5AR1,CLEC2D,CCL23,CDX4,RPS19,TBX19,MMP10,P2RX7,GH1,LTBP1,mir-124,LY9,S100A9,ABO,BMF,CD244,FCER1G,EYA1,PRKACB,IFNA8,UBASH3A,POU2AF1,TAL1,KLF6,SLPI,FBXW7,ETV1,CXCR1,IFNA14,NOX1,MTTP,RUNX2,ACSL5,CD226,CPB2,PPP1R1C,CYP27B1,CAST,MUS81,TBX5,IL5RA,TNFAIP6,OPRM1,PF4,TYK2,BMX,FYB,DPPA4,CD3G,IL18,CD180,IL1RN,SIRPA,AGTR1,PHOX2B,EHF,CCL3,NR3C1,RHOH,TGIF1,FAS,HDGF,RNASE3,S100A8,CFH,PPARG,GNAS,NPY1R,VANGL1,mir-506,FPR2,VSIG4,DEGS1,ERG,BTK,PMP22,GHR,ESM1,FGF20,PIM2 |
| Cellular Growth and Proliferation | men | 3.15E-05-2.08E-02 | PPARG,FYN,GAB2,DLL1,IRF4,SCN9A,DCN,MAPK8,POU3F2,IL1R1,DICER1,IL24,LTBP1,SPINK7,RCAN1,IL1RN,TLR1,AGTR1 |
| Immunological Disease | women | 3.38E-07-3.36E-03 | UBASH3A,SLPI,CTSG,CCL20,CXCR1,TXK,HAL,DEFA4,APCS,PRDM1,MUCL1,FGR,FASLG,TRIM21,TNFAIP6,OPRM1,C17orf59,TYK2,DEFA1 (includes others),TPM3,SCN10A,PDE8A,CD3G,IL18,IL1RN,TAGAP,RAMP3,ANG,SNCA,ECM1,AGTR1,SIRPA,IL1RL1,IL21R,CCL3,NR3C1,FAS,CHCHD2,RNASE3,S100A7,MBL2,S100A8,CFH,RNASE2,CCL1,CCR1,PPARG,CA10,CLEC2D,CCL23,NPY1R,FPR2,VSIG4,KRT5,P2RX7,GH1,CD3D,GHR,S100A9,LY9,CD244,FCER1G,FCRL3 |
| Immunological Disease | men | 3.44E-03-1.89E-02 | PPARG,FOXC2,SELE,IL1RN,CIITA,IL1R1,IL31,IL17F |
| Lymphoid Tissue Structure and Development | women | 1.62E-06-4.29E-03 | IFNA8,UBASH3A,POU2AF1,LMO2,TAL1,CCL20,CD37,IFNA14,F11,TXK,APCS,RUNX2,RARA,CD226,PRDM1,CYP27B1,CAST,LYVE1,TIMD4,FASLG,MARCO,IL5RA,OPRM1,PF4,TYK2,mir-193,CD3G,IL18,PCSK1,CD180,IL1RN,CD34,SIRPA,SLC4A4,IL1RL1,IL21R,GALNT1,CCL3,RHOH,FAS,MBL2,GBA,CCL1,PPARG,CCR1,C5AR1,NPY1R,FPR2,P2RX7,CD3D,LTBP1,BTK,GHR,BMF,CD244,FCER1G,EYA1,PIM2,FCAMR |
| Lymphoid Tissue Structure and Development | men | 1.01E-02-2.42E-02 | PPARG,FYN,FOXC2,DLL1,MMP14,VAV3,MGP,PLCG1,DICER1 |
| Molecular Transport | women | 1.64E-06-3.72E-03 | CCL20,BEST3,CXCR1,NOX1,TXK,SLC12A1,RARA,GPRC6A,CYP27B1,SLC39A2,CAST,FASLG,FGR,TRIM21,OPRM1,DEFA1 (includes others),BMX,TACR3,PLCL2,SCN10A,LILRB1,IL18,CD180,IL1RN,RAMP3,SLCO1B1,KCNE1,SNCA,SIRPA,AGTR1,SCN11A,SLC4A4,TREM1,IL1RL1,SLC17A6,SLC30A8,CCL3,NR3C1,AKAP7,FAS,SLN,GBA,S100A8,FXYD2,FGF7,CCL1,CCR1,PPARG,SLC34A1,C5AR1,GNAS,CLEC2D,NPY1R,CCL23,TRDN,ATP6V1E2,PMCH,FPR2,P2RX7,GH1,LTBP1,ATP2B2,BTK,mir-124,PMP22,GHR,S100A9,CD244,FCER1G,P2RY12,FCRL3 |
| Molecular Transport | men | 3.45E-04-2.6E-02 | PPARG,FYN,SLC9A3,PLCG1,IL1R1,CLDN7,AVPR1A,SLC6A3,ATP2B2,AQP9,DRD1,IL1RN,VAV3,CLOCK,SYT1,NRIP1,VDAC1,VGF,NUTF2,PPARGC1A |
| Cell Signaling | women | 2.55E-06-2E-03 | CCL20,CXCR1,TXK,SLC12A1,RARA,CAST,CYP27B1,FGR,TRIM21,OPRM1,PF4,DEFA1 (includes others),BMX,TACR3,PLCL2,LILRB1,IL18,CD180,CD34,AGTR1,SIRPA,TREM1,IL1RL1,CCL3,NR3C1,FAS,S100A8,GRIK2,FGF7,CCL1,CCR1,SLC34A1,C5AR1,GNAS,CCL23,NPY1R,TRDN,FPR2,PMCH,P2RX7,GH1,ATP2B2,LTBP1,BTK,mir-124,PMP22,GHR,S100A9,CD244,FCER1G,FCRL3 |
| Cell Signaling | men | 1.27E-02-1.89E-02 | SELE,DRD1,VDAC1,AVPR1A,AGTR1 |
| Vitamin and Mineral Metabolism | women | 2.55E-06-6.65E-04 | TREM1,IL1RL1,CCL20,CXCR1,CCL3,NR3C1,FAS,TXK,SLC12A1,S100A8,CYP27B1,CAST,GRIK2,FGF7,FGR,CCL1,CCR1,SLC34A1,TRIM21,GNAS,C5AR1,NPY1R,OPRM1,CCL23,PF4,TRDN,PMCH,FPR2,DEFA1 (includes others),BMX,TACR3,P2RX7,PLCL2,GH1,LILRB1,LTBP1,ATP2B2,BTK,mir-124,PMP22,IL18,CD180,S100A9,CD244,FCER1G,FCRL3,SIRPA,AGTR1 |
| Vitamin and Mineral Metabolism | men | 9.26E-03-1.89E-02 | MTRR,AKR1C3,ALDH1A3,ALDH1A2,VDAC1 |
| Embryonic Development | women | 4.5E-06-2.81E-03 | PRKACB,POU2AF1,LMO2,TAL1,CLEC3B,CD37,RUNX2,RARA,LUM,MGP,DMP1,GPRC6A,HOXD12,PRDM1,CYP27B1,CAST,LYVE1,FASLG,TBX5,DPPA4,FMOD,CD3G,IL18,IL1RN,ECM1,SNCA,SIRPA,SLC4A4,IL1RL1,IL21R,MEOX2,THBS3,NR3C1,FAS,S100A8,CCL1,PPARG,SEMA3E,CCR1,SLC34A1,CLEC2D,C5AR1,KRT5,P2RX7,CD3D,BTK,GHR,EVX2,FCER1G,FGF20,EYA1,FCAMR |
| Embryonic Development | men | 3.57E-04-2.6E-02 | FYN,SP6,MMP14,CIITA,POU3F2,SYNE1,DICER1,AGTPBP1,TGIF1,LIFR,FOXC2,ALDH1A3,MGP,UNC5C,PPARG,DLL1,SELE,DOT1L,MAPK8,JUNB,EPHA3,TCF7L1,LTBP1,ATP2B2,PLCB4,NRCAM,ALDH1A2,SUV420H1,ITGB6,PDX1,PPARGC1A |
| Hematopoiesis | women | 4.5E-06-4.29E-03 | IFNA8,POU2AF1,LMO2,TAL1,CCL20,IFNA14,F11,TXK,RUNX2,APCS,RARA,CD226,PRDM1,CAST,FASLG,IL5RA,OPRM1,PF4,TYK2,FYB,CD3G,IL18,PCSK1,CD180,IL1RN,CD34,GSTP1,SIRPA,SLC4A4,TREM1,IL1RL1,IL21R,CCL3,NR3C1,RHOH,FAS,PBXIP1,RNASE2,CCL1,PPARG,CCR1,C5AR1,NPY1R,CCL23,CDX4,RPS19,CD3D,ERG,LTBP1,BTK,BMF,CD244,FCER1G,FCAMR,PIM2 |
| Hematopoiesis | men | 1.01E-02-1.89E-02 | IL33,PPARG,GAB2,FYN,DLL1,IRF4,VAV3,MMP14,TLR1,PLCG1,DICER1,NLRC4 |
| Organ Development | women | 4.5E-06-2.81E-03 | PRKACB,POU2AF1,LMO2,TAL1,CLEC3B,CD37,MTTP,RUNX2,RARA,LUM,MGP,DMP1,GPRC6A,ABCB11,PRDM1,HOXD12,LYVE1,CYP27B1,CAST,FASLG,TBX5,OPRM1,PF4,FMOD,PDE8A,CD3G,IL18,IL1RN,SLCO1B1,ECM1,SIRPA,GSTP1,AGTR1,SLC4A4,IL1RL1,IL21R,MEOX2,THBS3,NR3C1,FAS,S100A8,CCL1,PPARG,SEMA3E,CCR1,SLC34A1,C5AR1,CLEC2D,KRT5,P2RX7,CD3D,BTK,EVX2,GHR,FCER1G,FGF20,EYA1,FCAMR |
| Organ Development | men | 4.44E-04-2.23E-02 | GAB2,SP6,FYN,MMP14,CIITA,POU3F2,SYNE1,DICER1,AGTPBP1,TGIF1,FOXC2,GLP2R,ALDH1A3,MGP,UNC5C,PPARG,ADORA3,SELE,MAPK8,IL1R1,EPHA3,ATP2B2,IL33,PLCB4,NRCAM,IL1RN,ALDH1A2,CLCN7,SUV420H1,ITGB6,PDX1,PPARGC1A |
| Organismal Development | women | 4.5E-06-2.81E-03 | PRKACB,LMO2,POU2AF1,KLF6,TAL1,CLEC3B,ETV1,FBXW7,CD37,NOX1,DSE,RUNX2,RARA,TPH2,LUM,DMP1,MGP,CPB2,ABCB11,GPRC6A,HOXD12,PRDM1,CYP27B1,CAST,LYVE1,MUS81,FASLG,TBX5,PLEC,FMOD,ANKRD26,CD3G,SLITRK6,IL18,PCSK1,IL1RN,RAMP3,SNCA,ECM1,SIRPA,AGTR1,SLC4A4,SLC17A6,IL1RL1,IL21R,MEOX2,THBS3,NR3C1,TGIF1,FAS,S100A8,GRIK2,CFH,ATN1,CCL1,PPARG,SEMA3E,CCR1,SLC34A1,C5AR1,CLEC2D,NPY1R,PMCH,KRT5,P2RX7,GH1,CD3D,ATP2B2,LTBP1,BTK,GHR,EVX2,BMF,FCER1G,FGF20,EYA1,PIM2,FCAMR |
| Organismal Development | men | 4.44E-04-2.42E-02 | FYN,SP6,MMP14,CIITA,POU3F2,SYNE1,CLDN7,DICER1,AVPR1A,AGTPBP1,TGIF1,SLC6A3,DSE,FOXC2,DRD1,ALDH1A3,MGP,IL17F,UNC5C,PPARG,SLC25A25,DLL1,SELE,DCN,MAPK8,DEDD,IL1R1,EPHA3,PDE4D,ARID3A,ATP2B2,RCAN1,PLCB4,NRCAM,IL1RN,REV1,VAV3,ALDH1A2,ADD1,HIST1H1D,CLCN7,NRIP1,VGF,SUV420H1,ITGB6,AGTR1,PDX1,PPARGC1A |
| Respiratory Disease | women | 5.27E-06-3.55E-03 | LILRA1,C9orf72,LMO2,CTSG,DLG2,MTX1,MUC7,PCDHB8,ZMAT1,KRT28,SCD5,TXK,RARA,LUM,DMP1,GPRC6A,CHAMP1,TIMD4,FASLG,DMBT1,RABL3,CD200R1,PLEC,DEFA1 (includes others),GPR111,mir-193,MLIP,LILRB1,DSG1,NRXN1,ANKRD26,SUCO,PCSK1,DHRS9,OR52I1,ACSM1,PLAC1L,C1orf105,TBC1D5,GSTP1,SCN11A,SLC4A4,MAGEA12,IL1RL1,SLC30A8,C8A,ZNF285,S100A7,MBL2,PCDH10,DCC,ASPH,CSN3,GPC6,ATN1,RNASE2,CCR1,SLC34A1,TREML2,FERD3L,MMP10,KRT5,P2RX7,GH1,DUSP21,LTBP1,ATP2B2,S100A9,LY9,CD244,UBN1,EYA1,NLRP14,DNAH1,TAL1,KLF6,SLPI,FBXW7,OR8B4,CAPS2,CXCR1,GPR148,SIGLEC12,MTTP,ATP11C,DEFA4,SLC12A1,WDPCP,TIGD2,ACSL5,FRMPD2,C20orf26,MARCO,TBX5,IGSF22,OR4C13,OR4S2,OPRM1,GLYAT,SP140,TACR3,CASC5,IL18,SLITRK6,C2orf71,JAKMIP2,DCHS2,SLCO1B1,KCNE1,MAK,AGTR1,PHOX2B,TREM1,C4orf17,SLC17A6,TNIP3,OR8B8,IL1F10,NR3C1,FAS,TGM5,PSKH2,PCDH8,CFH,PI4KB,PPARG,CA10,FCRLA,GOLGB1,FPR2,ERG,BTK,ESM1,DEFB116,FAM13A,ARPP21 |
| Respiratory Disease | men | 5.09E-08-2.04E-02 | GPR115,SNTG1,SYTL2,GDAP2,LCE4A,BTBD11,TMEM257,PCDHB8,NPSR1,ADAM7,ANGPT4,HIPK2,OR13C8,SPATA18,ZBTB16,ADORA3,DFNA5,CAPN6,SELE,RPL27,SCN9A,FCRL4,OR2AG2,mir-515,SLFN11,AQP9,IL17RB,NRCAM,FAM198B,MS4A4A,HIST1H3A (includes others),PLAC1L,OR1J4,SYT1,ITGB6,MMAA,SCN11A,NEB,ADAMTS14,COX7A2L,TBL3,OR5B3,REG4,SLC6A3,AKAP11,SPINK7,GLP2R,TTC23,TPO,ASPH,OR13G1,MKRN3,EPB41L4A,PLCG1,MYPN,EPHA3,SAA4,LTBP1,ATP2B2,GRM5,CALU,OR2T10,FAM107B,UBN1,TRIM48,OR10A3,NEDD9,LRP1,PPIP5K2,AKR1C3,RASA3,SLC9A3,TRIM51,KRTAP9-4,SYNE1,DICER1,B3GALT1,OR8J1,IL1R2,CTNNA2,CC2D2B,C20orf26,ABP1,KRT31,OR4K1,ZFYVE9,POLE4,TNNI3K,TIAM1,PCDHGA11,ZNF783,MAP3K13,OR10H4,SAP30BP,DCAF4L2,RGS6,IL33,NBPF15 (includes others),TTC14,IL1RN,BAZ2B,ASB15,DPP6,CLDN14,SYN3,AGTR1,FPR3,OR10T2,PCDHGA12,MMP14,ANKS4B,PRG4,HPS4,TMPRSS3,LTBP4,PCDH8,PCDHA13,GADL1,PPARG,SLC25A53,RP1,PCDHA12,IL1R1,PDE4D,NUP210L,ALS2,SLC5A8,PCDHGA5,TANGO6,VAV3,PIK3CB,RXFP3,OTUD6A,GLTSCR1L,A1CF |
| Cell Death and Survival | women | 8.87E-06-4.35E-03 | LMO2,SH3BGRL3,CTSG,FRMD6,SP110,CD37,EIF2A,TXK,APCS,RARA,LUM,MGP,DMP1,PRDM1,TIMD4,FGR,FASLG,TRIM21,PLEC,TPM3,DEFA1 (includes others),TPD52,FMOD,LILRB1,DSG1,ZBTB18,ACACA,FGA,ANG,SNCA,GSTP1,GALNT5,IL1RL1,IL21R,NAA35,MEOX2,C8A,MBL2,GBA,DCC,GRIK2,FGF7,ATN1,RNASE2,NADPH,C5AR1,CLEC2D,RPS19,MMP10,P2RX7,GH1,LTBP1,ATP2B2,S100A9,ABO,LY9,BMF,CD244,FCER1G,EYA1,FCAR,UBASH3A,POU2AF1,KLF6,TAL1,SLPI,FBXW7,CXCR1,NOX1,CCT8,F11,ATP11C,RUNX2,ACSL5,CD226,CPB2,CAST,MUS81,TBX5,IL5RA,PCDHGA6,OPRM1,PF4,TYK2,BMX,DPPA4,CD3G,IL18,IL1RN,NDC80,KCNE1,AGTR1,SIRPA,PHOX2B,SFTPC,TREM1,EHF,RASA4,CCL3,RHOH,NR3C1,FAS,ACAA2,HDGF,RNASE3,NEK7,S100A8,CFH,PI4KB,PPARG,GNAS,FAM134B,mir-506,ERG,BTK,PMP22,GHR,PIM2,PAFAH1B3 |
| Cell Death and Survival | men | 2.09E-03-2.42E-02 | FYN,GAB2,ST6GAL1,PCDHGA12,MMP14,ATF6,DICER1,PCDHGA3,SLC6A3,SPINK7,mir-154,CLOCK,HIPK2,HRK,ZBTB16,NLRC4,PPARG,ADORA3,SELE,DLL1,PCDHGA11,ST8SIA1,ARTN,DCN,MAPK8,AFP,JUNB,IL1R1,IL24,ALS2,PCDHGA5,LTBP1,GRM5,RCAN1,INSM1,IL17RB,NRCAM,IL1RN,CREM,ALDH1A2,CLCN7,VGF,LRP1,PDX1,PPARGC1A |
| Dermatological Diseases and Conditions | women | 9.67E-06-3.19E-03 | IFNA8,CTSG,SLPI,CLEC3B,CCL20,CXCR1,IFNA14,MTX1,VNN3,MUC7,TXK,HAL,RARA,MUCL1,FGR,FASLG,MARCO,TNFAIP6,OPRM1,TYK2,PLEC,DEFA1 (includes others),TPM3,BMX,FGFBP1,LILRB1,DSG1,IL18,IL1RN,LCE2C (includes others),RAMP3,SIRPA,GSTP1,CCL3,PDZK1IP1,NR3C1,FAS,RNASE3,S100A7,GBA,S100A8,FGF7,CCR1,PPARG,C5AR1,CCL23,KRT5,S100A9,FCER1G,S100A7A |
| Dermatological Diseases and Conditions | men | 3.44E-03-1.89E-02 | GRM5,FYN,FOXC2,BLOC1S3,IL1RN,SCN9A,HPS4,EDARADD,LTBP4,IL1R1,IL17F |
| Cellular Compromise | women | 1.91E-05-4.06E-03 | FCAR,TREM1,CCL3,NOX1,FAS,MTTP,GBA,CD226,FGR,FASLG,DMBT1,CCR1,NADPH,C5AR1,TREML2,PF4,CD200R1,PLEC,P2RX7,LTBP1,BTK,IL18,PCSK1,S100A9,CD244,FCER1G,SNCA,SIRPA,AGTR1 |
| Cellular Compromise | men | 2.09E-03-2.42E-02 | PPARG,SELE,MAPK8,ATF6,IL1R1,DICER1,ALS2,IL24,IL33,IL17RB,IL1RN,VAV3,AMICA1,HIPK2,PPARGC1A |
| Organismal Injury and Abnormalities | women | 6.86E-05-3.89E-03 | SLC4A4,TREM1,CTSG,SLPI,CXCR1,CCL3,NR3C1,FAS,F11,DTNA,APCS,RUNX2,TPH2,LUM,MGP,ABCB11,CFH,MUS81,FGR,FASLG,MARCO,PPARG,CCR1,C5AR1,NPY1R,OPRM1,TM4SF4,PLEC,KRT5,SCN10A,P2RX7,FMOD,GH1,PDE8A,IL18,PCSK1,S100A9,IL1RN,P2RY12,FGA,KCNE1,SCN11A,GSTP1,AGTR1 |
| Organismal Injury and Abnormalities | men | 1.31E-03-2.08E-02 | MMP14,HTR1D,DICER1,TGIF1,SLC6A3,FOXC2,DRD1,FKRP,HPS4,MGP,LTBP4,SNRPD3,IL17F,PPARG,SELE,BLOC1S3,DCN,SCN9A,UTS2,MAPK8,PLCG1,IL1R1,JUNB,STX2,PDE4D,ALS2,GRM5,RCAN1,IL1RN,VAV3,CREM,CLCN7,EDARADD,AGTR1,SCN11A,PPARGC1A |
| Connective Tissue Development and Function | women | 7.11E-05-3.72E-03 | PRKACB,SLC4A4,IL1RL1,TAL1,CLEC3B,MEOX2,NR3C1,THBS3,MTTP,APCS,RUNX2,RARA,LUM,DMP1,MGP,GPRC6A,S100A8,PRDM1,HOXD12,CYP27B1,CAST,FASLG,CCR1,PPARG,TBX5,SLC34A1,C5AR1,CLEC2D,NPY1R,KRT5,PLCL2,P2RX7,GH1,FMOD,ATP2B2,BTK,IL18,GHR,EVX2,IL1RN,FCER1G,FGF20,ECM1,EYA1,SIRPA |
| Connective Tissue Development and Function | men | 2.09E-03-2.6E-02 | PPARG,GAB2,DCN,MMP14,MAPK8,CIITA,DICER1,IL1R1,JUNB,ATP2B2,IL33,FOXC2,GLP2R,IL1RN,VAV3,CTBP2,MGP,CLCN7,LTBP4,HIPK2,TXLNG |
| Organ Morphology | women | 7.11E-05-3.93E-03 | PRKACB,SLC4A4,CLEC3B,MEOX2,NR3C1,RHOH,THBS3,FAS,F11,RUNX2,APCS,RARA,LUM,DMP1,MGP,HOXD12,CYP27B1,FASLG,MARCO,TBX5,SLC34A1,IL5RA,CLEC2D,NPY1R,FPR2,KRT5,FMOD,BTK,CD3G,PCSK1,GHR,EVX2,IL1RN,BMF,FCER1G,EYA1 |
| Organ Morphology | men | 2.09E-03-2.25E-02 | SP6,FYN,GAB2,MMP14,SYNE1,DICER1,CLDN7,FOXC2,CTNNA2,DRD1,MGP,PPARG,SELE,DCN,MAPK8,EPHA3,ATP2B2,PLCB4,NRCAM,IL1RN,ALDH1A2,KCNJ1,SUV420H1,ITGB6,VGF,AGTR1,PPARGC1A,PDX1 |
| Skeletal and Muscular System Development and Function | women | 7.11E-05-2.71E-03 | SLC4A4,PRKACB,IL1RL1,TAL1,CLEC3B,MEOX2,THBS3,RUNX2,RARA,LUM,MGP,DMP1,GPRC6A,S100A8,PRDM1,HOXD12,CYP27B1,CAST,FASLG,CCR1,TBX5,SLC34A1,C5AR1,CLEC2D,KRT5,PLCL2,P2RX7,FMOD,BTK,IL18,EVX2,GHR,IL1RN,FCER1G,ECM1,EYA1,SIRPA |
| Skeletal and Muscular System Development and Function | men | 3.57E-04-2.6E-02 | PPARG,GAB2,MMP14,DCN,CIITA,ACVR1,MAPK8,IL1R1,TCF7L1,DICER1,IL33,RCAN1,FOXC2,GLP2R,IL1RN,CALD1,MGP,CLCN7,VDAC1,ZBTB16,AGTR1,PPARGC1A,TXLNG |
| Cell Morphology | women | 1.26E-04-3.97E-03 | SLC4A4,FCAR,IL1RL1,POU2AF1,TAL1,FBXW7,MEOX2,CCL3,NR3C1,RHOH,FAS,F11,GBA,RUNX2,DMP1,MGP,PRDM1,CAST,FASLG,FGR,PPARG,CCR1,GNAS,C5AR1,IL5RA,OPRM1,TYK2,PLEC,TPM3,P2RX7,GH1,ATP2B2,BTK,CD3G,IL18,GHR,PCSK1,CD180,LY9,IL1RN,BMF,FCER1G,P2RY12,ACACA,SNCA,CD34,SIRPA,PIM2 |
| Cell Morphology | men | 8.57E-04-2.6E-02 | FYN,SP6,MMP14,SYNE1,DICER1,FOXC2,DTNA,MGP,PPARG,ADORA3,SELE,TIAM1,IRF4,MAPK8,IL24,ALS2,PLD1,LTBP1,IL33,GNAI3,RCAN1,AQP9,PLCB4,mir-30,NRCAM,IL1RN,VAV3,CREM,ITGB6,AGTR1,TXLNG,PDX1,PPARGC1A |
| Gastrointestinal Disease | women | 1.38E-04-3.55E-03 | TREM1,SLPI,NR3C1,FAS,MTTP,F11,ATP11C,DCC,ABCB11,S100A8,CFH,FGR,FASLG,DMBT1,PPARG,TM4SF4,OPRM1,PF4,TYK2,P2RX7,GH1,CD3D,PDE8A,CD3G,IL18,GHR,PCSK1,IL1RN,FGA,SLCO1B1,GSTP1,AGTR1 |
| Gastrointestinal Disease | men | 1.45E-02-1.89E-02 | PPARG,FOXC2,ADORA3,IL1RN,LTBP4,EDARADD,ASPH,STX2,PDX1 |
| Developmental Disorder | women | 1.63E-04-3.55E-03 | SLC34A1,GNAS,PLEC,KRT5,GH1,FAS,GHR,EVX2,RUNX2,GPRC6A,HOXD12,CYP27B1,FASLG |
| Developmental Disorder | men | 3.57E-04-1.89E-02 | SP6,NEB,MMP14,CIITA,SYNE1,DICER1,TGIF1,SLC6A3,IFT140,mir-154,POLR1C,FOXC2,FKRP,LARP7,HPS4,LTBP4,ASPH,HIPK2,ZBTB16,ADORA3,MTRR,BLOC1S3,TRHR,DCN,MAPK8,PDE4D,ALS2,IL33,RCAN1,GNAI3,PLCB4,mir-30,VAV3,CREM,CLCN7,EDARADD,MBNL1,ADAMTSL4,MMAA,DEAF1,AGTR1,PPARGC1A |
| Hereditary Disorder | women | 1.63E-04-3.55E-03 | CCR1,SLC34A1,GNAS,PLEC,KRT5,CCL3,GH1,FAS,GHR,EVX2,IL1RN,RUNX2,GPRC6A,HOXD12,CYP27B1,FASLG |
| Hereditary Disorder | men | 3.57E-04-1.89E-02 | GPR115,SYNE1,POU3F2,DICER1,mir-154,IFT140,LIFR,FOXC2,TAS2R38,FKRP,miR-544-3p (and other miRNAs w/seed UUCUGCA),MGP,IL17F,ZBTB16,BLOC1S3,SCN9A,DCN,SAP30BP,AFP,HIBCH,NBPF15 (includes others),IL1RN,DPP6,SYN3,EDARADD,CLCN7,CLDN14,SYT1,ADAMTSL4,MMAA,VDAC1,AGTR1,PDX1,NEB,MMP14,CIITA,HTR1D,TGIF1,SLC6A3,AKAP11,DTNA,POLR1C,PRG4,DRD1,SLC12A9,LARP7,HPS4,PITPNM1,CLOCK,LTBP4,TPO,PCDH8,PPARG,MTRR,TRHR,ACVR1,IL1R1,PDE4D,ALS2,TAAR6,ATP2B2,GRM5,GNAI3,PLCB4,KCNJ1,MBNL1,VGF,EHBP1 |
| Metabolic Disease | women | 1.63E-04-3.55E-03 | PPARG,SFTPC,SLC34A1,GNAS,TBX19,DMP1,GPRC6A,CYP27B1,NR3C1,ECM1,FASLG,FAS |
| Metabolic Disease | men | 1.2E-03-2.42E-02 | PPARG,SELE,MTRR,BLOC1S3,TRHR,MAPK8,HTR1D,IL1R1,GRM5,HIBCH,FOXC2,DRD1,IL1RN,KCNJ1,HPS4,LTBP4,TPO,MMAA,VGF,AGTR1,PDX1,PPARGC1A,UNC5C |
| Cardiovascular System Development and Function | women | 2.1E-04-6.57E-04 | LMO2,TAL1,SLPI,FBXW7,MEOX2,NOX1,FAS,F11,RUNX2,RARA,LUM,MGP,PRDM1,CFH,MUS81,FGF7,FGR,FASLG,CCL1,SEMA3E,PPARG,PF4,DEFA1 (includes others),BMX,P2RX7,GH1,FMOD,ERG,LTBP1,IL18,GHR,IL1RN,ECM1,ANG,EYA1,TM4SF1,AGTR1 |
| Cardiovascular System Development and Function | men | 1.06E-03-2.47E-02 | MMP14,SYNE1,DICER1,AVPR1A,FOXC2,DRD1,IL20,CTBP2,TLR1,MGP,ANGPT4,ASPH,IL17F,HIPK2,ARHGAP24,PPARG,ADORA3,SELE,ARHGEF12,DCN,UTS2,MAPK8,ACVR1,JUNB,IL1R1,EPHA3,RGS6,IL24,PLD1,LTBP1,RCAN1,IL1RN,VAV3,ALDH1A2,CREM,ADD1,AGTR1,PPARGC1A |
| Hepatic System Disease | women | 2.38E-04-2.81E-03 | PPARG,TM4SF4,OPRM1,PF4,P2RX7,GH1,NR3C1,FAS,MTTP,F11,PDE8A,IL18,IL1RN,ABCB11,CFH,FGA,SLCO1B1,FGR,AGTR1,FASLG,GSTP1 |
| Hepatic System Disease | men | 1.45E-02-1.45E-02 | ADORA3,PDX1 |
| Cardiovascular Disease | women | 2.63E-04-4.04E-03 | STMN2,IL1RL1,KLF6,DLG2,SP110,NR3C1,EIF2A,FAS,F11,APCS,RARA,MGP,CPB2,S100A8,GRIK2,MUS81,FASLG,RNASE2,TBX5,CCR1,PPARG,TRIM21,PF4,SCN10A,GH1,LTBP1,PDE8A,IL18,GHR,S100A9,ABO,IL1RN,P2RY12,FGA,EYA1,AGTR1,GSTP1 |
| Cardiovascular Disease | men | 3.16E-03-2.41E-02 | GAB2,MMP14,DICER1,NEXN,TGIF1,SLC6A3,FOXC2,DTNA,ASIC5,DRD1,MGP,LTBP4,ASPH,PPARG,ADORA3,SELE,SCN9A,UTS2,MAPK8,JUNB,EPHA3,IL1R1,RGS6,PDE4D,LTBP1,IL33,RCAN1,IL17RB,mir-30,IL1RN,VAV3,FAM107B,CREM,DPP6,LRP1,AGTR1,PPARGC1A |
| Reproductive System Disease | women | 3.13E-04-3.55E-03 | KLF6,ETV1,ZNF285,NR3C1,PCDHB8,RUNX2,S100A8,ASPH,CFH,PPARG,TBX19,BMX,TACR3,GH1,LILRB1,ERG,RPA2,PCSK1,GHR,S100A9,FAM13A,JAKMIP2,CD34,AGTR1,GSTP1,SCN11A |
| Reproductive System Disease | men | 2.45E-03-1.89E-02 | PPARG,SELE,MGST1,CAPN6,DCN,MMP14,AFP,JUNB,IL1R1,PDE4D,PTP4A1,ATP2B2,SLC6A3,IL1R2,PLCB4,mir-30,ALDH1A3,VAV3,NRIP1,ZBTB16,NEDD9,EHBP1,ISG20 |
| Digestive System Development and Function | women | 3.37E-04-3.14E-03 | NR3C1,FAS,MTTP,GBA,RUNX2,TPH2,DCC,ABCB11,CSN3,ATN1,FASLG,PPARG,SLC34A1,OPRM1,NPY1R,PF4,PMCH,TACR3,ANKRD26,PDE8A,IL18,CRHBP,GHR,IL1RN,FGF20,SLCO1B1,GSTP1,AGTR1 |
| Digestive System Development and Function | men | 9.89E-04-1.89E-02 | SP6,ADORA3,MMP14,ASPH,IL1R1,PDX1 |
| Hepatic System Development and Function | women | 3.37E-04-1.76E-03 | PPARG,OPRM1,PF4,NR3C1,FAS,MTTP,PDE8A,IL18,IL1RN,ABCB11,SLCO1B1,AGTR1,FASLG,GSTP1 |
| Hepatic System Development and Function | men | 1.45E-02-1.89E-02 | ADORA3,TLR1,PDX1 |
| Lipid Metabolism | women | 3.61E-04-3.28E-03 | SLC4A4,ETV1,CXCR1,NR3C1,ACAA2,FAS,NOX1,MTTP,SCD5,GBA,RUNX2,RARA,ACSS2,ACSL5,DCC,CYP27B1,FGF7,FGR,FASLG,PI4KB,CCR1,PPARG,NADPH,C5AR1,GNAS,NPY1R,OPRM1,PMCH,P2RX7,DEGS1,PDE8A,BTK,IL18,GHR,DHRS9,TMEM55A,IL1RN,FCER1G,ACSM1,ACACA,SLCO1B1,ANG,SNCA,KCNE1,AGTR1,PAFAH1B3 |
| Lipid Metabolism | men | 3.45E-04-1.89E-02 | PPARG,FYN,ST8SIA1,TRHR,AKR1C3,SLC9A3,PLCG1,IL1R1,CLDN7,B3GALT1,AVPR1A,PLD1,DRD1,IL1RN,ALDH1A3,VAV3,ALDH1A2,PITPNM1,CLOCK,PIK3CB,NRIP1,VGF,PPARGC1A |
| Small Molecule Biochemistry | women | 3.61E-04-3.28E-03 | ETV1,CXCR1,NOX1,MTTP,DSE,SCD5,SLC12A1,RUNX2,RARA,ACSL5,GPRC6A,CPB2,CYP27B1,FGR,FASLG,TNFAIP6,OPRM1,PF4,PLCL2,PDE8A,IL18,DHRS9,TMEM55A,IL1RN,ACSM1,ACACA,SLCO1B1,ANG,KCNE1,SNCA,AGTR1,SLC4A4,GALNT5,SLC30A8,CCL3,NR3C1,ACAA2,FAS,GBA,ACSS2,DCC,GRIK2,FGF7,PI4KB,CCL1,CCR1,PPARG,NADPH,SLC34A1,C5AR1,GNAS,CLEC2D,CCL23,NPY1R,TRDN,PMCH,P2RX7,DEGS1,ATP2B2,BTK,GHR,FCER1G,PAFAH1B3 |
| Small Molecule Biochemistry | men | 3.45E-04-2.6E-02 | FYN,AKR1C3,SLC9A3,CLDN7,B3GALT1,AVPR1A,AGTPBP1,SLC6A3,CHDH,DRD1,ALDH1A3,CLOCK,PITPNM1,ABP1,GADL1,PPARG,MTRR,ST8SIA1,TRHR,PLCG1,IL1R1,PLD1,ATP2B2,AQP9,REV1,IL1RN,VAV3,ALDH1A2,PIK3CB,NRIP1,VDAC1,VGF,PPARGC1A |
| Amino Acid Metabolism | women | 3.69E-04-3.17E-03 | IL1RN,CPB2,P2RX7,SLCO1B1,NR3C1,SNCA |
| Amino Acid Metabolism | men | 1.89E-02-1.89E-02 | GADL1 |
| Cellular Assembly and Organization | women | 3.69E-04-3.55E-03 | PLEC,FASLG,FAS |
| Cellular Assembly and Organization | men | 1.3E-03-2.46E-02 | FYN,NEB,ADAMTS14,PCDHGA12,DICER1,SLC16A4,PCDHGA3,AVPR1A,AKAP11,FOXC2,DTNA,MYOM1,CALD1,PITPNM1,SORBS2,HIPK2,PPARG,SELE,TIAM1,PCDHGA11,ARHGEF12,DCN,RP1,MAPK8,JUNB,STX2,PCDHGA5,PLD1,GNAI3,VAV3,ADD1,SYT1,ADAMTSL4,VGF,LRP1 |
| Endocrine System Disorders | women | 3.69E-04-3.55E-03 | PCSK1,GNAS,GHR,TBX19,TACR3,GH1,NR3C1 |
| Endocrine System Disorders | men | 1.45E-02-2.08E-02 | PPARG,SELE,TRHR,HTR1D,DICER1,IL1R1,SLC5A8,SLC6A3,FOXC2,DRD1,IL1RN,KCNJ1,CREM,TPO,AGTR1,PDX1,PPARGC1A |
| Visual System Development and Function | women | 3.69E-04-2.16E-03 | SEMA3E,LUM,LYVE1,FMOD,NR3C1,CCL1 |
| Visual System Development and Function | men | 1.18E-02-1.18E-02 | ALDH1A3,ALDH1A2 |
| Tumor Morphology | women | 6.22E-04-1.09E-03 | LY9,RARA,CD244,CD226,FASLG,FAS |
| Tumor Morphology | men | 3.15E-05-1.89E-02 | PPARG,GAB2,SELE,DCN,POU3F2,IL1R1,DICER1,PDE4D,IL24,SPINK7,RCAN1,IL1RN,AMICA1,PIK3CB,ZBTB16 |
| Carbohydrate Metabolism | women | 6.97E-04-4.37E-03 | CCR1,GNAS,TNFAIP6,GALNT5,NPY1R,PF4,CTSG,CXCR1,P2RX7,DSE,BTK,IL18,TMEM55A,APCS,IL1RN,DCC,CHIA,FGF7,PI4KB |
| Carbohydrate Metabolism | men | 7.22E-03-2.42E-02 | PPARG,ADORA3,MTRR,ST8SIA1,TRHR,PLCG1,ATF6,DICER1,AVPR1A,B3GALT1,PLD1,DSE,GRM5,IL1R2,DRD1,IL1RN,CTBP2,PITPNM1,PIK3CB,AGTR1,PPARGC1A |
| Hypersensitivity Response | women | 7.66E-04-1.04E-03 | FCAR,IL5RA,IL1RL1,CCL3,SIRPA,FAS |
| Hypersensitivity Response | men | 7.03E-03-7.03E-03 | IL33,MAPK8 |
| Behavior | women | 8.62E-04-2.16E-03 | PPARG,SLC34A1,OPRM1,NPY1R,PMCH,TACR3,ANKRD26,SRGAP3,GHR,IL1RN,TPH2,DCC,CSN3,SNCA,TIMD4,ATN1 |
| Behavior | men | 2.75E-04-2.54E-02 | FYN,TRHR,SYNE1,IL1R1,DICER1,ALS2,AVPR1A,SLC6A3,GRM5,CTNNA2,PLCB4,NPSR1,DRD1,TAS2R38,IL1RN,CDC14B,CREM,CLOCK,VDAC1,VGF,PPARGC1A |
| Respiratory System Development and Function | women | 8.93E-04-3.55E-03 | SFTPC,RARA,FGF7,NR3C1,FAS |
| Respiratory System Development and Function | men | 2.64E-03-2.49E-02 | SP6,SELE,MMP14,ACVR1,MAPK8,SYNE1,DICER1,EPHA3,TGIF1,ALDH1A3,ALDH1A2,MGP,ASPH,ITGB6,SUV420H1,PPARGC1A |
| Neurological Disease | women | 1.09E-03-3.55E-03 | CCR1,DTNA,S100A9,IL1RN,OPRM1,DLG2,CCL3,NR3C1,SNCA,GSTP1,AGTR1,EIF2A,RNASE2 |
| Neurological Disease | men | 7.26E-05-2.61E-02 | GPR115,GAB2,POU3F2,SYNE1,DICER1,LIFR,IFT140,NAT8,STRN4,ITIH4,miR-544-3p (and other miRNAs w/seed UUCUGCA),FKRP,MGP,ANGPT4,KLRC3,HIPK2,ZBTB16,ADORA3,DLL1,SELE,CAPN6,TIAM1,ICA1,DCN,SCN9A,SAP30BP,PTPN3,JUNB,NBPF15 (includes others),NRCAM,IL1RN,MS4A4A,ALDH1A2,CREM,SYN3,CLDN14,CLCN7,SYT1,VDAC1,MMAA,COX5B,AGTR1,SCN11A,FYN,ADAMTS14,MMP14,HTR1D,NAT2,TGIF1,SLC6A3,AKAP11,DTNA,PRG4,DRD1,SLC12A9,LARP7,CLOCK,PITPNM1,NDUFS2,PCDH8,SNRPD3,HRK,PPARG,ST8SIA1,RP1,PLCG1,EPHA3,IL1R1,TCF7L1,PDE4D,ALS2,ARL3,TAAR6,ATP2B2,GRM5,RCAN1,PLCB4,REV1,VAV3,NRIP1,UCK2,VGF,LRP1,PPARGC1A |
| Ophthalmic Disease | women | 1.09E-03-3.96E-03 | LUM,SCN10A,FMOD,NR3C1 |
| Ophthalmic Disease | men | 1.91E-04-1.89E-02 | IFT140,ADORA3,FOXC2,MGST1,BLOC1S3,DCN,FKRP,HPS4,ADAMTSL4,LGSN,LTBP1 |
| Reproductive System Development and Function | women | 1.21E-03-1.21E-03 | SLC34A1,GHR,GBA,RUNX2,NEK7,DPPA4 |
| Reproductive System Development and Function | men | 5.18E-04-2.6E-02 | PLCZ1,FYN,PLCG1,AFP,ASPH,VGF,PDE4D,AVPR1A |
| Renal and Urological Disease | women | 1.37E-03-3.55E-03 | PPARG,SLC34A1,CLEC3B,CCL3,LILRB1,FAS,CRHBP,IL1RN,GPRC6A,CYP27B1,GRIK2,FXYD2,CD34,AGTR1 |
| Renal and Urological Disease | men | 1.48E-02-2.42E-02 | IFT140,PPARG,FOXC2,DRD1,VAV3,DCN,MMP14,KCNJ1,MMAA,PDE4D,TGIF1,AGTR1 |
| Post-Translational Modification | women | 2E-03-2E-03 | CCR1,GHR,RARA,CD244,FCER1G,FCRL3,GH1,CCL3,FGF7,CD34,SIRPA,FAS |
| Post-Translational Modification | men | 1.45E-02-1.89E-02 | ST6GAL1,ST8SIA1,GADL1 |
| Hair and Skin Development and Function | women | 2.16E-03-2.16E-03 | FASLG,FAS |
| Hair and Skin Development and Function | men | 1.75E-02-1.89E-02 | SP6,TIAM1,DICER1,ITGB6 |
| Nutritional Disease | women | 2.16E-03-2.16E-03 | GHR,GH1 |
| Nutritional Disease | men | 8.51E-03-8.51E-03 | SLC25A25,PPARG,SCN9A,MAPK8,HTR1D,IL1R1,PDE4D,AVPR1A,SLC6A3,FOXC2,IL1RN,CLOCK,NRIP1,VGF,SCN11A,LRP1,AGTR1,PPARGC1A |
| Nervous System Development and Function | women | 2.2E-03-2.2E-03 | PMP22,P2RX7,ISL2 |
| Nervous System Development and Function | men | 4.44E-04-2.6E-02 | FYN,PCDHGA12,PCDHB2,SYNE1,HTR1D,POU3F2,DICER1,PCDHGA3,AGTPBP1,TGIF1,SLC6A3,PCDHB8,DTNA,CTNNA2,DRD1,ALDH1A3,PCDH8,HIPK2,UNC5C,PCDHGA11,ST8SIA1,ARTN,SCN9A,UTS2,MAPK8,GPR176,ALS2,PCDHGA5,ATP2B2,GRM5,PLCB4,NRCAM,IL1RN,ALDH1A2,DPP6,SYT1,VDAC1,VGF,PPARGC1A |
| Endocrine System Development and Function | women | 3.28E-03-3.28E-03 | SLC34A1,CLEC2D,OPRM1,PMCH,CYP27B1,NR3C1 |
| Endocrine System Development and Function | men | 3.45E-04-1.89E-02 | PPARG,AKR1C3,SLC9A3,VAV3,CLOCK,CLDN7,AVPR1A,PDX1 |
| Cell Cycle | women | 3.55E-03-3.55E-03 | BTK,CD180 |
| Cell Cycle | men | 9.26E-03-1.89E-02 | PPARG,VAV3,DCN,CREM,DOT1L,PLCG1,DICER1,NRIP1,HIPK2 |
| Gene Expression | women | 3.55E-03-3.55E-03 | GHR,GH1 |
| Gene Expression | men | 1.89E-02-2.12E-02 | SP6,WHSC1,CIITA,UIMC1,ATF6,ZNF366,POU3F2,DICER1,TGIF1,FOXC2,ZNF263,FUBP3,ZBTB14,CTBP2,CLOCK,IL17F,HIPK2,TFAP2D,ZBTB16,TFEC,PPARG,SRA1,DLL1,IRF4,LRRFIP1,GRHL1,HIST2H3C (includes others),ACVR1,MAPK8,DEDD,JUNB,TCF7L1,LDB2,ARID3A,GRM5,IL33,ETV3,RCAN1,CREM,UBN1,NRIP1,DEAF1,HBG1,PDX1,PPARGC1A,TXLNG |
| Psychological Disorders | women | 3.55E-03-3.55E-03 | SNCA,GSTP1 |
| Psychological Disorders | men | 7.26E-05-1.89E-02 | GPR115,GAB2,ADAMTS14,SYNE1,POU3F2,HTR1D,DICER1,AVPR1A,AKAP11,SLC6A3,NAT8,MYOM1,DRD1,ITIH4,SLC12A9,miR-544-3p (and other miRNAs w/seed UUCUGCA),PITPNM1,CLOCK,PCDH8,PPARG,ADORA3,TIAM1,CAPN6,SCN9A,SAP30BP,PLCG1,IL1R1,TCF7L1,PDE4D,ATP2B2,TAAR6,GRM5,RCAN1,NBPF15 (includes others),MS4A4A,IL1RN,CREM,SYN3,SYT1,MMAA,VDAC1,VGF,DEAF1,LRP1,SCN11A,AGTR1,PPARGC1A |
| Renal and Urological System Development and Function | women | 3.55E-03-3.55E-03 | P2RX7,FAS |
| Renal and Urological System Development and Function | men | 7.69E-03-2.54E-02 | FOXC2,FYN,VAV3,MMP14,KCNJ1,MGP,PLCG1,CLDN7,NRIP1,SLC5A8,AGTR1 |
