# Supplementary material for: DNA Methylation Signature of Childhood Chronic Physical Aggression in T Cells of Both Men and Women
Source: PLoS One. 2014 Jan 24;9(1):e86822. doi: 10.1371/journal.pone.0086822 (PMC3901708; doi:10.1371/journal.pone.0086822)
Supplement: Table S6 — Canonical pathway enriched with genes whose methylation is associated with aggression in both sexes from IPA analysis (women n = 430 genes and men n = 448 genes). (DOCX) [file pone.0086822.s008.docx]

**Supplementary Table S6. Canonical pathway enriched with genes whose methylation is associated with aggression in both sexes from IPA analysis (women n=430 genes and men n=448 genes).**

| **Ingenuity Canonical Pathways** | **Analysis Name** | **P-value** | **Ratio** | **# Molecules** | **Molecules** |
| --- | --- | --- | --- | --- | --- |
| Granulocyte Adhesion and Diapedesis | women | 0.00015136 | 6.74E-02 | 12 | IL18,C5AR1,CCL23,IL1RN,IL1RL1,PF4,FPR2,MMP10,CCL20,IL1F10,CCL3,CCL1 |
|  | men | 0.00051286 | 6.18E-02 | 11 | IL33,IL1R2,FPR3,GNAI3,SELE,IL1RN,MMP14,CLDN18,CLDN14,IL1R1,CLDN7 |
| Agranulocyte Adhesion and Diapedesis | women | 0.00025119 | 6.35E-02 | 12 | IL18,C5AR1,CCL23,IL1RN,PF4,MMP10,CCL20,IL1F10,CXCR1,CCL3,CD34,CCL1 |
|  | men | 0.00870964 | 4.76E-02 | 9 | IL33,GNAI3,SELE,IL1RN,MMP14,CLDN18,CLDN14,IL1R1,CLDN7 |
| FXR/RXR Activation | women | 0.00052481 | 7.92E-02 | 8 | PPARG,IL18,IL1RN,RARA,ABCB11,IL1F10,SLCO1B1,MTTP |
|  | men | 0.00977237 | 5.94E-02 | 6 | IL33,PPARG,IL1RN,FETUB,MAPK8,PPARGC1A |
| IL-10 Signaling | women | 0.00263027 | 7.69E-02 | 6 | CCR1,IL18,IL1RN,IL1RL1,TYK2,IL1F10 |
|  | men | 0.01174898 | 6.41E-02 | 5 | IL33,IL1R2,IL1RN,MAPK8,IL1R1 |
| Role of Cytokines in Mediating Communication between Immune Cells | women | 0.0040738 | 9.09E-02 | 5 | IL18,IFNA8,IL1RN,IL1F10,IFNA14 |
|  | men | 0.00380189 | 9.09E-02 | 5 | IL33,IL20,IL1RN,IL17F,IL24 |
| Tec Kinase Signaling | women | 0.00026303 | 6.04E-02 | 11 | BTK,GNG4,TXK,GNAS,TYK2,FCER1G,BMX,RHOH,FGR,FAS,FASLG |
|  | men | 0.03162278 | 3.85E-02 | 7 | FYN,GNAI3,GNG11,VAV3,MAPK8,PLCG1,PIK3CB |
| p38 MAPK Signaling | women | 0.02630268 | 5.08E-02 | 6 | IL18,IL1RN,IL1RL1,IL1F10,FAS,FASLG |
|  | men | 0.02454709 | 5.08E-02 | 6 | IL33,IL1R2,IL1RN,HIST2H3C (includes others),IL1R1,MKNK2 |
| LPS/IL-1 Mediated Inhibition of RXR Function | women | 0.02884032 | 3.73E-02 | 9 | IL18,IL1RN,CYP2A13,IL1RL1,RARA,ACSL5,ABCB11,IL1F10,GSTP1 |
|  | men | 0.01023293 | 4.15E-02 | 10 | IL33,IL1R2,MGST1,IL1RN,ALDH1A3,ALDH1A2,MAPK8,IL1R1,HS3ST4,PPARGC1A |
| LXR/RXR Activation | women | 0.01096478 | 5.15E-02 | 7 | IL18,IL1RN,IL1RL1,ACACA,S100A8,IL1F10,FGA |
|  | men | 0.03235937 | 4.41E-02 | 6 | IL33,IL1R2,IL1RN,ITIH4,IL1R1,SAA4 |
| PPAR Signaling | women | 0.03548134 | 4.76E-02 | 5 | PPARG,IL18,IL1RN,IL1RL1,IL1F10 |
|  | men | 0.00040738 | 7.62E-02 | 8 | IL33,PPARG,IL1R2,SRA1,IL1RN,IL1R1,NRIP1,PPARGC1A |
| NF-κB Signaling | women | 0.04168694 | 4.02E-02 | 7 | PRKACB,IL18,GHR,IL1RN,FCER1G,IL1F10,GH1 |
|  | men | 0.03890451 | 4.02E-02 | 7 | IL33,IL1R2,IL1RN,TLR1,MAPK8,PIK3CB,IL1R1 |
| T Cell Receptor Signaling | women | 0.04677351 | 4.59E-02 | 5 | BTK,CD3G,TXK,BMX,CD3D |
|  | men | 0.04466836 | 4.59E-02 | 5 | FYN,VAV3,MAPK8,PLCG1,PIK3CB |
| Acute Phase Response Signaling | women | 0.04786301 | 3.89E-02 | 7 | IL18,MBL2,APCS,IL1RN,IL1F10,FGA,NR3C1 |
|  | men | 0.04466836 | 3.89E-02 | 7 | IL33,IL1RN,ITIH4,MAPK8,PIK3CB,IL1R1,SAA4 |
