# Supplementary material for: DNA Methylation Signature of Childhood Chronic Physical Aggression in T Cells of Both Men and Women
Source: PLoS One. 2014 Jan 24;9(1):e86822. doi: 10.1371/journal.pone.0086822 (PMC3901708; doi:10.1371/journal.pone.0086822)
Supplement: Table S7 — Upstream regulators showing a significant overlap with genes whose methylation is associated with aggression in both sexes from IPA analysis (women n = 430 genes and men n = 448 genes). (DOCX) [file pone.0086822.s009.docx]

**Supplementary Table S7. Upstream regulators showing a significant overlap with genes whose methylation is associated with aggression in both sexes from IPA analysis (women n=430 genes and men n=448 genes).**

| Analysis | Upstream Regulator | Molecule Type | p-value of overlap | Target molecules in dataset |
| --- | --- | --- | --- | --- |
| women | 15-LOX | group | 0.0379 | PPARG |
| men | 15-LOX | group | 0.0394 | PPARG |
| women | 20-hydroxyprostaglandin E2 | chemical - endogenous mammalian | 0.0191 | PPARG |
| men | 20-hydroxyprostaglandin E2 | chemical - endogenous mammalian | 0.0199 | PPARG |
| women | 3M-001 | chemical drug | 0.0358 | CCL3,IFNA14,IFNA8 |
| women | 3M-011 | chemical reagent | 0.0268 | CCL3,IFNA14,IFNA8 |
| women | ADAMTS12 | peptidase | 0.013 | MGP,S100A8,S100A9 |
| men | ADAMTS12 | peptidase | 0.0144 | ALDH1A3,IL33,MGP |
| women | BCMO1 | enzyme | 0.0379 | PPARG |
| men | BCMO1 | enzyme | 0.0394 | PPARG |
| women | BMPER | other | 0.0411 | MGP,RUNX2 |
| men | BMPER | other | 0.044 | ACVR1,MGP |
| women | clenbuterol | chemical drug | 0.0248 | CAST,IL1RN |
| men | clenbuterol | chemical drug | 0.0266 | IL1RN,PPARGC1A |
| women | concanamycin A | chemical drug | 0.0325 | FASLG,PPARG |
| men | concanamycin A | chemical drug | 0.0349 | PPARG,SELE |
| women | Creb5 | transcription regulator | 0.0191 | PPARG |
| men | Creb5 | transcription regulator | 0.0199 | PPARG |
| women | DNAJC14 | other | 0.0191 | AGTR1 |
| men | DNAJC14 | other | 0.0199 | AGTR1 |
| women | endrin | chemical toxicant | 0.0379 | PPARG |
| men | endrin | chemical toxicant | 0.0394 | PPARG |
| women | ESR2 | ligand-dependent nuclear receptor | 0.0332 | GSTP1,KRT20,LTBP1,PLEC,RARA,RUNX2 |
| men | ESR2 | ligand-dependent nuclear receptor | 0.0389 | IL20,LTBP1,MMP14,NEDD9,SELE,VAV3 |
| women | FOS | transcription regulator | 0.00006 | AGTR1,CAST,CD244,ESM1,FASLG,FGA,GBA,GRIK2,GSTP1,IL1RL1,KLF6,LTBP1,MMP10,PRDM1,RARA,S100A8,S100A9,SEMA3E,SIRPA,SLPI,TAL1,TPD52,TREM1 |
| men | FOS | transcription regulator | 0.00855 | AGTR1,AKR1C3,ALDH1A3,ARID3A,CALU,DEDD,FETUB,HRK,JUNB,LTBP1,MGST1,NBPF15 (includes others),NRIP1,PIK3CB,PLD1,PTP4A1,SYT1,VAV3 |
| women | gamma tocopherol | chemical drug | 0.0379 | PPARG |
| men | gamma tocopherol | chemical drug | 0.0394 | PPARG |
| women | GC-GCR dimer | complex | 0.0367 | IL1RN,SLPI |
| men | GC-GCR dimer | complex | 0.0394 | IL1R2,IL1RN |
| women | HOXA9 | transcription regulator | 0.0163 | CCL3,CD34,CD93,CPB2,CTSG,ERG,FPR2,S100A8 |
| men | HOXA9 | transcription regulator | 0.02 | ARL3,CLCN7,CREM,HBG1,JUNB,MBNL1,NEB,SELE |
| women | ICAM3 | transmembrane receptor | 0.0191 | PPARG |
| men | ICAM3 | transmembrane receptor | 0.0199 | PPARG |
| women | IL1 | group | 0.000092 | ACACA,CCL20,CCL23,CCL3,CD34,FAS,FASLG,FGF7,IL1RL1,IL1RN,IL5RA,MMP10,MTTP,NOX1,PPARG,RUNX2,TNFAIP6 |
| men | IL1 | group | 0.02 | DCN,IL1R1,IL1R2,IL1RN,IL33,IRF4,LRP1,PLD1,PPARG,PPARGC1A,SAA4,SELE |
| women | IL1B | cytokine | 0.000013 | ABCB11,AGTR1,APCS,BMF,CCL1,CCL20,CCL3,CCR1,CPB2,EHF,FAS,FASLG,FGF7,FPR2,GHR,IL18,IL1RL1,IL1RN,MMP10,P2RX7,PCSK1,PPARG,RARA,RNASE7,RUNX2,S100A8,S100A9,SLC12A1,SLCO1B1,SNCA,STMN2,TNFAIP6,TREM1 |
| men | IL1B | cytokine | 0.00316 | AGTR1,AVPR1A,CIITA,CREM,DCN,HRK,IL17F,IL1R1,IL1R2,IL1RN,IL20,IL24,IL33,JUNB,KCNJ1,LIFR,MMP14,PDX1,PLD1,PPARG,PPARGC1A,PTP4A1,RCAN1,SCN9A,SELE,SLC25A25,SLC9A3 |
| women | IL4 | cytokine | 0.000588 | C5AR1,CCL1,CCL20,CCL23,CCL3,CXCR1,DSG1,FAS,FASLG,FCER1G,FPR2,IL18,IL1RL1,IL1RN,IL5RA,MARCO,MMP10,NR3C1,OPRM1,PIM2,POU2AF1,PPARG,PRDM1,S100A8,S100A9,SIRPA,TXK |
| men | IL4 | cytokine | 0.0464 | ALDH1A2,CIITA,HIPK2,IL17F,IL17RB,IL1R1,IL1R2,IL1RN,IL24,IL31,IL33,IRF4,ISG20,JUNB,LIFR,NEDD9,PLD1,PPARG,PPARGC1A,SELE,TFEC |
| women | miR-615-3p (miRNAs w/seed CCGAGCC) | mature microRNA | 0.0379 | PPARG |
| men | miR-615-3p (miRNAs w/seed CCGAGCC) | mature microRNA | 0.0394 | PPARG |
| women | Myosin2 | complex | 0.0191 | PPARG |
| men | Myosin2 | complex | 0.0199 | PPARG |
| women | oleylamide | chemical - endogenous mammalian | 0.0379 | PPARG |
| men | oleylamide | chemical - endogenous mammalian | 0.0394 | PPARG |
| women | ORM1 | other | 0.0191 | IL1RN |
| men | ORM1 | other | 0.0199 | IL1RN |
| women | OSM | cytokine | 0.0196 | CCL20,DEGS1,ECM1,FGA,IL18,LCE2C (includes others),MMP10,PDZK1IP1,PPARG,PRDM1,S100A7,S100A8,S100A9,SLPI,TAL1,TM4SF1,ZBTB18 |
| men | OSM | cytokine | 0.000606 | ACOT2,AFP,AKR1C3,AQP9,ARHGEF12,DNAH17,FETUB,IL1R2,IL33,ISG20,JUNB,LIFR,LRRFIP1,MKNK2,PLCB4,PPARG,PTP4A1,SELE,ST8SIA1,SYNE1,TPO,ZNF263 |
| women | PPP3CA | phosphatase | 0.0376 | FASLG,GSTP1,PMP22,RUNX2 |
| men | PPP3CA | phosphatase | 0.0423 | HRK,MGST1,PLD1,RCAN1 |
| women | prostaglandin E2 | chemical - endogenous mammalian | 0.000334 | CCL20,CCL3,CCR1,CLEC2D,FAS,FASLG,FMOD,IL1RN,PPARG,PRDM1,RUNX2,S100A8,SLC12A1,TNFAIP6,TREM1 |
| men | prostaglandin E2 | chemical - endogenous mammalian | 0.0103 | CREM,DCN,ENPP3,IL17F,IL1R1,IL1R2,IL1RN,IRF4,MMP14,NEDD9,PPARG,SCN9A |
| women | Rbp | group | 0.0379 | PPARG |
| men | Rbp | group | 0.0394 | PPARG |
| women | RETSAT | enzyme | 0.0379 | PPARG |
| men | RETSAT | enzyme | 0.0394 | PPARG |
| women | Scd2 | enzyme | 0.0191 | PPARG |
| men | Scd2 | enzyme | 0.0199 | PPARG |
| women | SLC27A4 | transporter | 0.0379 | PPARG |
| men | SLC27A4 | transporter | 0.0394 | PPARG |
| women | SMURF2 | enzyme | 0.0212 | RUNX2,TGIF1 |
| men | SMURF2 | enzyme | 0.0228 | NEDD9,TGIF1 |
| women | SOCS7 | other | 0.0379 | PPARG |
| men | SOCS7 | other | 0.0394 | PPARG |
| women | Sos | group | 0.0161 | CAST,ESM1,GBA,KLF6,MMP10,SEMA3E,SIRPA,SLPI,TPD52 |
| men | Sos | group | 0.0201 | AKR1C3,ALDH1A3,ARID3A,DEDD,FETUB,MGST1,NRIP1,PIK3CB,PLD1 |
| women | SPI1 | transcription regulator | 0.0356 | CD180,CXCR1,FCER1G,IL18,IL1RN,OPRM1,TREM1 |
| men | SPI1 | transcription regulator | 0.0423 | CIITA,IL1R2,IL1RN,IL24,IRF4,SP6,TFEC |
| women | TNF | cytokine | 0.00000549 | ABCB11,ACACA,AGTR1,APCS,ARRDC3,C5AR1,CARD18,CCL1,CCL20,CCL3,CCR1,CXCR1,CYP27B1,DMBT1,EHF,ERG,ESM1,FAS,FASLG,FCAR,FCER1G,FPR2,GHR,GPR84,GSTP1,IL18,IL1RL1,IL1RN,IL21R,KLF6,LYVE1,MBL2,MEOX2,MGP,MMP10,MTTP,NR3C1,OPRM1,PIM2,PPARG,PRDM1,RARA,RNASE2,RUNX2,S100A7,S100A8,S100A9,SLC12A1,SLPI,TGIF1,TM4SF1,TNFAIP6,TREM1,TYK2 |
| men | TNF | cytokine | 0.00661 | AGTR1,ALDH1A3,AQP9,AVPR1A,CIITA,CREM,DCN,ENPP3,FOXC2,GNAI3,GPR176,GPR84,HIPK2,HRK,IL17RB,IL1R1,IL1R2,IL1RN,IL20,IL24,IL33,IRF4,ITGB6,JUNB,KCNJ1,LIFR,MGP,MGST1,MMP14,NEDD9,NRIP1,PDX1,PIK3CB,PPARG,PPARGC1A,RCAN1,SCN9A,SELE,SLC5A8,SLC9A3,ST6GAL1,TGIF1,TPO,WHSC1 |
| women | TRDN | other | 0.0379 | ASPH |
| men | TRDN | other | 0.0394 | ASPH |
| women | vitamin K2 | chemical drug | 0.00458 | AGTR1,DSE,MGP |
| men | vitamin K2 | chemical drug | 0.0051 | AGTR1,DSE,MGP |
| women | Zfp54 | other | 0.0191 | PPARG |
| men | Zfp54 | other | 0.0199 | PPARG |
| women | Zfp55 | other | 0.0191 | PPARG |
| men | Zfp55 | other | 0.0199 | PPARG |
