# Supplementary material for: DNA Methylation Signature of Childhood Chronic Physical Aggression in T Cells of Both Men and Women
Source: PLoS One. 2014 Jan 24;9(1):e86822. doi: 10.1371/journal.pone.0086822 (PMC3901708; doi:10.1371/journal.pone.0086822)
Supplement: Table S8 — Gene promoters with significant changes in methylation between women CPA and NPA groups from both MeDIP-microarrays and Illumina 450k arrays analysis. (DOCX) [file pone.0086822.s010.docx]

**Supplementary Table S8. Gene promoters with significant changes in methylation between women CPA and NPA groups from both MeDIP-microarrays and Illumina 450k arrays analysis.**

ANKRD22

ARPP21

ATP8B4

BMF

C17orf59

C20orf26

CCL1

CCR1

CD244

CD3D

CD3G

CLEC2D

CPB2

CTAGE5

CTSG

DEGS1

DHRS9

DTNA

ECM1

ERG

FAM134B

FAS

FASLG

FCAMR

FCAR

FCER1G

FGR

FXYD2

FYB

GALNT1

GNAS

HAL

HTRA4

IGSF6

IL1RL1

IL1RN

IL21R

KCNE1

LMO2

LOC644248

LTBP1

LY9

MARCO

MGP

MS4A3

NDUFS2

OTUB2

P2RY12

PAFAH1B3

PLCL2

PLEKHM3

POU2AF1

PRDM1

RHOH

RNASE11

RNASE2

RNASE7

S100A8

SLN

SLPI

SMIM3

TBC1D5

THBS3

TIGD2

TMEM71

TNFAIP6

TREM1

TXK

UBASH3A

ZNF366
