# Supplementary material for: DNA Methylation Signature of Childhood Chronic Physical Aggression in T Cells of Both Men and Women
Source: PLoS One. 2014 Jan 24;9(1):e86822. doi: 10.1371/journal.pone.0086822 (PMC3901708; doi:10.1371/journal.pone.0086822)
Supplement: Methods S1 — (DOCX) [file pone.0086822.s011.docx]

**Supplementary Methods.**

**Assessment of subjects’ familial adversity, behavior problems, psychiatric diagnoses and criminal records.**

**Familial adversity.** The index of family adversity is a composite score of the degree of adversity in families ranging from 0 to 1, which has been used regularly with this cohort. The index includes parent’s level of education, type of employment, age at the birth of their first child and marital status when the subjects were age 6 [16,95].

**Physical aggression and other behavior problems.** In the course of the longitudinal studies, teachers annually rated the frequency of girls’ physical aggression, opposition, hyperactivity, inattention and anxiety from kindergarten to secondary school with the Social Behavior Questionnaire [95]. The physical aggression ratings were used to trace the developmental trajectories and create the CPA group and the control group (see [6] and [11] for details of the trajectory analyses).

**Criminal record.** Canadian youth between 13 and 17 years who commit delinquent acts are referred to the juvenile courts. Subjects who were found guilty by a juvenile court were identified from official records. Using official records we also identified subjects in each group who had been convicted of a criminal offence between 18 and 24 years.

**Mental disorders.** When the subjects were 15 years, structured psychiatric interviews using a French translation of the Diagnostic Interview Schedule for Children-2.25 (DISC-2.25) [96] were used with the mothers and the subjects to estimate the prevalence of DSM-III-R diagnoses such as: simple phobia, anxiety of separation, generalized anxiety, hyper anxiety, major depression, dysthymia, oppositional disorder and conduct disorder, over the previous 6 months [87]. Subjects were also asked whether they had a psychiatric record during the interview at 21 years.
